# Challenges to fracture service availability and readiness provided by allopathic and traditional health providers: national surveys across The Gambia and Zimbabwe

**DOI:** 10.7189/jogh.15.04082

**Published:** 2025-03-14

**Authors:** Anya Burton, Tadios Manyanga, Hannah Wilson, Landing Jarjou, Matthew L Costa, Simon Graham, James Masters, Momodou K Jallow, Samuel Hawley, Momodou T Nyassi, Prudance Mushayavanhu, Munyaradzi Ndekwere, Rashida A Ferrand, Kate A Ward, Kebba S Marenah, Celia L Gregson

**Affiliations:** 1 Musculoskeletal Research Unit, University of Bristol, Bristol, England, UK; 2 The Health Research Unit Zimbabwe, Biomedical Research and Training Institute, Harare, Harare Province, Zimbabwe; 3 Medical Research Council Unit The Gambia, London School of Hygiene and Tropical Medicine, Banjul, The Gambia; 4 Oxford Trauma and Emergency Care, Nuffield Department of Orthopaedics, Rheumatology and Musculoskeletal Science, University of Oxford, Oxford, England, UK; 5 Ministry of Health, The Gambia Government, Banjul, The Gambia; 6 Department of Surgery, Sally Mugabe Central Hospital, Harare, Zimbabwe; 7 Department of Surgery, Midlands State University, Gweru, Midlands Province, Zimbabwe; 8 Clinical Research Department, London School of Hygiene and Tropical Medicine, London, England, UK; 9 Medical Research Council Lifecourse Epidemiology Centre, University of Southampton, Southampton, England, UK; 10 Department of Orthopaedics & Trauma, Edward Francis Small Teaching Hospital, Banjul, The Gambia

## Abstract

**Background:**

Populations in Africa are ageing, hence the number of age-related fragility fractures, including hip fractures, is rising. Hip fractures are an indicator condition for older adult health provision, as they require a multifaceted pathway of care. To enable health service planning, detailed national-level understanding of current fracture service provision is needed.

**Methods:**

The WHO Service Availability & Readiness Assessment survey was modified to evaluate fracture service availability, and readiness. All health care facilities to which a patient with a hip fracture could present in The Gambia and Zimbabwe were invited to participate between October 2021 and January 2023. A further traditional bone-setter (TBS)-specific survey assessed TBS care in The Gambia. Availability of services per 100 000 adults ≥ 18 years, and general, fracture-specific, and hip fracture-specific care readiness were determined.

**Results:**

All invited facilities in Zimbabwe (n = 186), 98% in The Gambia (n = 150), and 35 of 42 (83%) TBS participated in the survey. General availability of hospital facilities was low in both Zimbabwe and The Gambia and many facilities lacked regular electricity, reliable oxygen supplies, and sharp/infectious waste disposal. In The Gambia, 78.6% public hospitals and 53.8% other facility types (*e.g.* NGO/mission) had no doctors. Fracture care readiness: < 1 orthopaedic surgeon was available for 100 000 adults in both countries. Orthopaedic trained nurses, physiotherapists, and occupational therapists were few. Only 10 (6.7%) facilities in The Gambia and 56 (30.1%) in Zimbabwe had functioning X-ray facilities. Equipment for fracture immobilisation was widely unavailable. No public facility had a dual-energy X-ray absorptiometry scanner; antiresorptive treatment access was limited to < 5% facilities. Hip fracture readiness: only four facilities in The Gambia and 17 in Zimbabwe could offer surgery. Inpatient delays for surgery were long, especially in Zimbabwe. Non-operative management was common in Zimbabwe and in those visiting TBS in The Gambia. Over half TBS (51.4%) reported being able to set a hip fracture, management included traditional medicines (57.1%), splinting (20.0%), manipulation (14.3%) and traction (5.7%). Only 14.3% TBS referred hip fractures to hospital.

**Conclusions:**

Findings highlight multiple important modifiable gaps in care which warrant urgent focus, with recommendations made, given expected increases in fragility fractures and need for universal health coverage.

A decade ago, musculoskeletal conditions including fractures were already accounting for 21% of years lived with disability worldwide [1]. Fractures are often caused by high impact trauma, such as road traffic accidents; however, as longevity increases the contribution from fragility fractures, caused by low impact falls (from standing height or less) is growing. Between 2015 and 2050, the number of older adults is expected to increase more rapidly in sub-Saharan Africa than in any other region, in part due to increasing life expectancy [2,3]. As such the number of fragility fractures are expected to increase across Africa, including hip fractures which convey high rates of morbidity and mortality [4–6]. Health services, which have largely evolved to provide episodic infectious disease, paediatric, and maternal care, are now needing to provide orthopaedic trauma care, often to older people with multimorbidity. In general, fracture services usually include provision of:

1. emergency trauma assessment and management

2. emergency radiographic imaging to diagnose fracture(s)

3. pain management and immobilisation of a fracture (in the case of hip fractures this includes use of traction)

4. operative management for fracture fixation where needed

5. post fracture rehabilitation

6. assessment and management of further fragility fracture risk/osteoporosis

7. patient education.

To enable planning of health services, there is a need to understand current fracture services. In West Africa, these services are frequently provided by traditional bone-setters (TBS), usually prior to, but sometimes following, or in combination with, allopathic fracture care [7].

The World Health Organization (WHO) developed the Service Availability and Readiness Assessment (SARA) tool to quantify and monitor health services, to generate objective and reliable evidence to support the planning and management of healthcare systems [8]. There are three main focus areas of SARA:

1. service availability refers to the presence and distribution of facilities, inpatient beds, and healthcare workers

2. general service readiness refers to the overall capacity of health facilities to provide general healthcare services (*e.g.* family planning, obstetric care), and is defined by the availability of components required to provide services, such as essential medicines, basic amenities, infection control precautions, and diagnostic capacity

3. service-specific readiness refers to the ability of health facilities to provide a specific service, it considers the quality of a service and is measured through consideration of tracer items that include trained staff, clinical guidelines, equipment, diagnostic capacity *etc*. [8].

Using a modified WHO SARA survey framework, this study aimed to quantify service availability and general service readiness for fracture care, and hip fracture service-specific readiness in Zimbabwe and The Gambia. These countries reflect two diverse, economically challenged healthcare services in different geographical regions within Africa. The modified SARA assessment included assessment of fracture care provision by TBS in The Gambia.

## METHODS

### Study setting

The SARA survey, modified to focus on fracture service availability and readiness, was conducted in facilities across two African countries, The Gambia in the West, and Zimbabwe in the South [8]. The Gambia, one of the most densely populated countries in Africa, is a small country, stretching 450 km along the Gambia River, and is surrounded by Senegal except for its Western Atlantic Ocean coastline. The population is 2.7 million (36% rural dwelling), growing 2.5% annually, and life expectancy at birth is now 62 years [9]. Gross domestic product (GDP) is low at 840 USD per capita, with 2.6% of GDP spent on health [9].

Zimbabwe has a population of 16.3 million, 68% rural dwelling, and life expectancy at birth is 59 years [9]. Gross domestic product is higher at 1267 USD per capita, with 3.4% spent on healthcare [9]. As of 2020, The Gambia has the 6th lowest, and Zimbabwe the 19th lowest percent GDP expenditure on health globally. In contrast to the mix of TBS and allopathic care delivered in The Gambia, Zimbabwean fracture care is mainly allopathic and delivered in hospitals and health centres.

### Principal facility list

A principal facility list (PFL) [10] was created for each country from pre-complied lists held by the WHO and Department of Health directorates, and supplemented with local knowledge. All sectors (public, private, faith-based organisations, non-government organisations (NGOs)) were included, and the level of service provision (*e.g.* community health centre, district hospital, provincial hospital) recorded.

The Ministry of Health Regional Health Directorates hold lists of registered TBS in The Gambia, though not all TBS are registered. Therefore creating a PFL required a different approach. It was constructed through the West Coast and Kanifing Regional Health Directorate lists, knowledge from clinical, academic, and social networks, and by using a ‘snowballing’ approach through word-of-mouth from each TBS.

### Data collection

The survey constituted two questionnaires: a hospital service questionnaire and a fracture service questionnaire; the latter focused on provision of diagnostic imaging and treatment for hip fractures. Hip fractures were chosen as, of all osteoporotic fragility fractures, they place the greatest demands on fracture services [11,12]. Questionnaire completion was designed to be flexible to local preferences and national travel restrictions, so it could be completed either directly by the hospital team online or by a study researcher in-person, via video call, or over the phone [13]. Data were collected between October 2021 and January 2023. The GPS location of each facility was recorded either by the study researcher or the respondent. All surveys were checked by local researchers, data managers, and the central study data team. Incomplete or ambiguous responses were clarified with the relevant hospital team. In The Gambia, TBS were given verbal and written study information, and asked to complete a consent form, before proceeding to complete a TBS-specific interviewer-administered questionnaire. All operational facilities on the PFL where a patient with a hip fracture could present for clinical assessment, including for stabilisation and onward referral, were eligible for inclusion and invited to participate, other than the numerous small primary care level community health centres (CHCs), sometimes known as rural health centres, in Zimbabwe. Facilities being constructed or closed were excluded.

### Availability of facilities, beds and staff

Facilities were classified by level of provision and managing authority across both countries:

1. publicly funded health centres and clinics

2. publicly funded rural and district hospitals

3. publicly funded regional and provincial hospitals

4. publicly funded central hospitals

5. privately funded facilities

6. all other facilities (faith-based, NGOs, research institutions, and service (*e.g.* clinics in army barracks)).

Facilities’ availability was quantified to calculate total inpatient beds, adult trauma and orthopaedic (T&O) beds, and facility density per 100 000 of the adult population (*i.e.* aged ≥ 18 years). Healthcare workforce quantity and density per 100 000 adults were calculated, counting part-time staff as 0.5 full time equivalent, as per SARA guidelines [8]. Global Burden of Disease population projections to 2019 [14], extrapolated to 2022 assuming linear growth, were used for density calculations as the latest census data were only available for 2013 in The Gambia and 2012 in Zimbabwe.

### General service readiness to provide fracture care in Zimbabwe and The Gambia

General service readiness was assessed through availability of tracer items across four domains:

1. basic amenities, seven-items (*e.g.* electricity, telecommunication equipment, patient transport, water and toilets)

2. basic equipment, five-items (*e.g.* weight scales, thermometers)

3. infection control precautions, eight-items (*e.g.* waste disposal, gloves)

4. diagnostic capacity, two-items (haemoglobin and HIV testing).

Each tracer item was marked as available (score of one) or absent (score of zero) and, for each facility, the number of tracer items that were present in each domain was summed and the percent of available tracer items per domain derived (*i.e.* the domain score, adapted from SARA [8]). A general readiness score was generated for each facility as the mean of the four domain scores (basic amenity domain score + basic equipment domain score + infection control precautions domain score + diagnostic capacity domain score/4), therefore each domain contributed 25% to the general readiness score. Selected items regarding surgical and blood transfusion readiness were also assessed and availability quantified.

General fracture service readiness was evaluated through quantification of staff involved in fracture care, *e.g.* orthopaedic-trained surgical and nursing staff and physiotherapists, availability of radiographic imaging facilities and trained staff, and provision of basic fracture care. Availability of walking aids and osteoporosis management were assessed.

### Hip fracture-specific service readiness in Zimbabwe and The Gambia

Hip fracture-specific service readiness was evaluated through quantification of staff involved in hip fracture care, *e.g.* in provision of hip fracture care, and volume of patient attendances. The quality of hip fracture care pathway was assessed, aiming to understand the initial management of suspected hip fractures, timing of surgery, availability of surgical equipment (including implants), clinical reasoning around operative choices and non-operative decision making, inpatient complications, physiotherapy provision and practice, lengths of hospital stay, and use of guidelines supporting hip fracture care. Facilities were asked to provide annual/monthly hip fracture presentation numbers, either from records (if available) or estimated.

### Traditional bone-setters in The Gambia

Traditional bone-setter services were assessed through reporting of practice, including duration of TBS work, training received, location of work, workload, and injury-management approaches (*e.g.* manual manipulation, splinting, herbal remedies).

### Data management and statistical analysis

Data were collected and managed using Research Electronic Data Capture (REDCap) tools, hosted by the University of Bristol. Research Electronic Data Capture is a secure, web-based software platform designed to support data capture for research studies [15,16]. It provides an interface for validated data capture, audit trails for tracking data manipulation, and automated export procedures for data downloads. The REDCap Mobile Application allowed data capture offline in areas with limited connectivity [17]. In inbuilt data validation and quality checks were performed regularly. Data were exported to Stata v18.0 (StatCorp, Texas, USA, 2023) for analyses.

To describe service availability and readiness, indicator numbers (counts) and percentages were used for categorical data, and median (MDN) or mean (x̄) with range (minimum-maximum) for continuous data. As many staff work across both public and private sectors, data are presented by sector. To assess repeatability of data collection at facilities, 10% of health facilities were randomly selected and revisited face-to-face to re-collect data. Agreement, beyond that expected by chance alone, was calculated for selected key variables using Kappa statistics [18].

### Ethical and governance approvals

Ethical and governance approvals for study protocols were obtained from the following Institutional Review Boards:

• The Gambia: The Gambia Government/MRC Unit The Gambia@LSHTM Scientific Coordinating Committee and Ethics committee (22/04/2021 ref. 22975); Ministry of Health (20/08/2021 ref. DDHS/AD/2021/08(MTN27))

• Zimbabwe: The Medical Research Council of Zimbabwe (14/07/2021 ref. MRCZ/A/2706); The Biomedical Research and Training Institute (19/02/2021 ref. AP161/2021); Sally Mugabe Central Hospital (29/01/2021 ref. HCHEC/ 250121/06); The University of Zimbabwe College of Health Sciences and the Parirenyatwa group of hospitals (25/02/2021); Harare City Health (27/01/2021).

## RESULTS

### Health facilities

**Figure 1:**
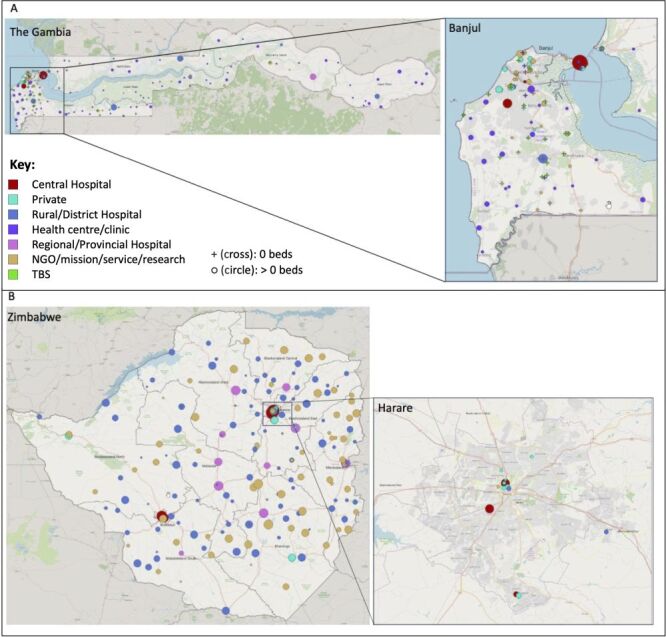
Facilities and TBS surveyed in The Gambia (**Panel A**) and facilities surveyed in Zimbabwe (**Panel** **B**). Larger circles indicate more beds; 1–10 beds, 11–30 beds, 31–50 beds, 51–100 beds, 101–200 beds, 201–400 beds, 401–800 beds, 801–1200 beds. Created using QGIS [19], DIVA-GIS [20] and OpenStreetMap [21]. TBS – traditional bone-setter.

In The Gambia, of an initial 188 medical facilities identified, 35 were excluded as they were either non-operational, duplicates or not existing, or were a small facility to which a hip fracture would not be expected to present, leaving a total of 153 in the final PFL (Figure S1 in the **Online Supplementary Document**). Three of these (2%) declined to participate. All data were collected face-to-face by researcher administered questionnaire. Of 150 participating facilities, 98 (65.3%) were public, 8.7% private and 26.0% other types (*i.e.* NGO, faith-based, research, and service facilities) (**Figure 1**). Of the public facilities, three were central hospitals (including one tertiary referral equivalent), three were regional/provincial hospitals, eight were rural or district hospitals, and 84 were health centres or clinics; 32.7% of facilities served urban areas and 66.7% rural. Central hospitals provided for urban populations, whilst other facility types provided for both urban and rural populations; 11 of 13 private facilities provided for urban populations (**Table 1**).

**Table 1:** Health facility type, distribution and health service availability in The Gambia and Zimbabwe

Variables		The Gambia							Zimbabwe						
		**Health centre/clinic**	**Rural/district hospital**	**Regional/provincial hospital**	**Central hospital**	**Private**	Other*	**Total**	**Rural/district hospital**	**Regional/provincial hospital**	**Central hospital**	**Private**	**Faith-based**	**Total**
**Facilities**	N	84	8	3	3	13	39	150	101	10	5	10	60	186
	%	56.0	5.3	2.0	2.0	8.7	26.0	100.0	54.3	5.4	2.7	5.4	32.3	100.0
**Catchment**														
Urban/semi-urban	N	12	2	1	3	11	20	49	12	5	0	7	4	28
	%	14.3	25.0	33.3	100.0	84.6	51.3	32.7	11.9	50.0	0.0	70.0	6.7	15.1
Rural/semi-rural	N	72	6	2	0	1	19	100	69	0	0	0	48	117
	%	85.7	75.0	66.7	0.0	7.7	48.7	66.7	68.3	0.0	0.0	0.0	80.0	62.9
Mixed	N	0	0	0	0	1	0	1	20	5	5	3	8	41	
	%	0.0	0.0	0.0	0.0	7.7	0.0	0.7	19.8	50.0	100.0	30.0	13.3	22.0	
**Health infrastructure**														
Facilities	N	84	8	3	3	13	39	150	101	10	5	10	60	186
	Density†	6.7	0.6	0.2	0.2	1.0	3.1	12.0	1.2	0.1	0.1	0.1	0.7	2.2
Adult, non-maternity inpatient beds	N	734	445	206	564	187	340	2476	4765	1936	3151	749	4703	15304
	Density†	58.9	35.7	16.5	45.2	15.0	27.3	198.6	56.6	23.0	37.4	8.9	55.9	181.8
Adult T&O inpatient beds	N	46	48	18	28	24	26	190	293	209	313	94	165	1074
	Density†	3.7	3.9	1.4	2.2	1.9	2.1	15.2	3.5	2.5	3.7	1.1	2.0	12.8

T&O – Trauma and orthopaedic

*Non government organisation/faith-based/service/research.

†Density per 100 000 adults.

In Zimbabwe, of 193 medical facilities identified, seven were excluded, leaving 186 in the final PFL (Figure S1 in the **Online Supplementary Document**); all participated in the survey (**Figure 1**). Data were collected online (89.2%), face-to-face (10.2%) and via virtual researcher-administrated questionnaire (0.5%). Overall, 116 (62.4%) facilities were public, 10 (5.4%) were private and 60 (32.3%) were other (all of which were faith-based). Of the public facilities, five were central hospitals, 10 were regional/provincial hospitals, and 101 were rural or district hospitals. Overall, 15.1% of facilities served urban areas, 62.9% rural and 22.0% both. All facility types were available to urban and rural populations (**Table 1**).

### Availability

#### Beds

In the Gambia, in total there were 12.0 facilities per 100 000 adults available for hip fracture presentations, including CHCs (**Table 1**). Overall, 2476 adult inpatient beds (excluding maternity beds) were reported, generating a density of 198.6 per 100 000 adults, of which 1949 (78.7%) were provided by public facilities. Many beds (n = 1179, 47.6%) were in health centres, or rural or district hospitals. However, of all inpatient beds, only 190 (7.7%) were available for T&O care, of which 94 (49.5%) were in health centres, or rural or district hospitals. Most orthopaedic surgery in The Gambia, is conducted in just one of the three central hospitals; this hospital has only 28 dedicated T&O care beds.

In Zimbabwe, there were 2.2 facilities per 100 000 adults available for hip fracture presentations (not including CHCs). Overall, 15 304 inpatient beds were reported, providing a density of 181.8 per 100 000 adults; 64.4% were provided by public, 30.7% faith-based and 4.9% by private facilities. Of all inpatient beds, 1074 (7.0%) were T&O care beds, 75.9% of which were in public sector health facilities.

#### Staffing

**Figure 2:**
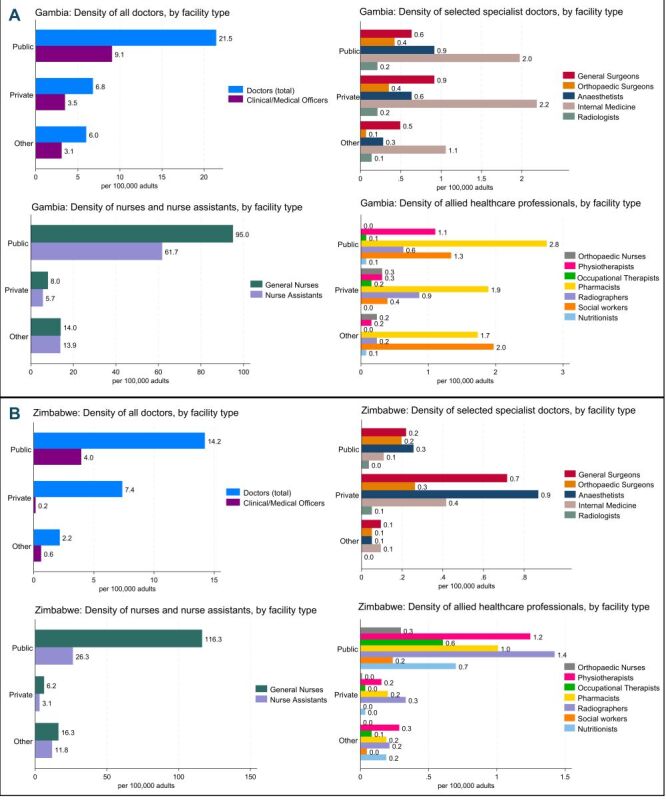
Density of healthcare staff in The Gambia (**Panel A**) and Zimbabwe (**Panel B**). Specialist doctor categories, geriatricians and rheumatologists excluded as there were < 0.1 /100 000.

In total, 427 doctors were enumerated in The Gambia (34.3 per 100 000); 62.6% in the public, 19.8% private, and 17.5% in other sector facilities (**Figure 2**, Panel A; Table S1 in the **Online Supplementary Document**). These included 25 general surgeons (non-orthopaedic surgeons including those in post-graduate training) (2.0 / 100 000), 10.5 (0.8 / 100 000) orthopaedic surgeons, 23 anaesthetists (1.8 / 100 000) but no rheumatologists. General nurses (n = 1460; 117.1 / 100 000), mostly worked in the public sector (81.1%), as did 75.9% of nurse aids/attendants (n = 1013; 81.3 / 100 000). There were few orthopaedic trained nurses (n = 7; 0.6 / 100 000, all working in the private or faith-based sectors), physiotherapists (n = 20; 1.6 / 100 000), pharmacists (n = 80; 6.4 / 100 000) and radiographers (n = 22; 1.7 / 100,000), and even fewer occupational therapists (n = 3; 0.2 / 100 000) or nutritionists (n = 2; 0.2 / 100 000).

In Zimbabwe, 2000 doctors were enumerated (23.8 per 100 000), 59.9% in public, 31.0% in private, and 9.1% in faith-based facilities (**Figure 2**, Panel B; Table S1 in the **Online Supplementary Document**). These included 87 (1.0 / 100 000) general surgeons, 43 (0.5 / 100 000) orthopaedic surgeons (38.6% working in public, 51.4% private and 10.0% in faith-based facilities), 99 (1.2 / 100 000) anaesthetists and 1.2 (< 0.1 / 100 000) rheumatologists. General nurses (n = 11 677; 138.7 / 100 000) mostly worked in public facilities (83.8%). There were fewer healthcare assistants/nurse aids (n = 3467, 41.2 / 100 000). There were few orthopaedic trained nurses (n = 26; 0.3 / 100 000, 96.2% worked in the public sector), physiotherapists (n = 142; 1.7 / 100 000), pharmacists (n = 118, 1.4 / 100 000), occupational therapists (n = 61; 0.7 / 100 000) and nutritionists (n = 78; 0.9 / 100 000). Neither country had a geriatrician. Whilst 166 radiographers (2.0 / 100 000) were identified, 17 facilities (nine public and eight faith-based) had available and functional radiography facilities but no radiographers, instead, X-ray technicians were operating the equipment.

### Readiness

#### General service readiness – basic amenities

Within the basic amenity domain, all seven items were only available in 8% of Gambian facilities. In The Gambia, the mean percent of the seven basic amenity tracer items available per facility was 68.5% (Table S2 in the **Online Supplementary Document**), meaning on average facilities were lacking 31.5% of these items. Rooms with audio and visual privacy, and a computer with internet access, were rare (available in 32 and 38% of facilities, respectively). Only 61% of facilities had reliable electricity, including only one (of the three) central hospital. All five basic equipment items (scale, thermometer, stethoscope, blood pressure apparatus, light source) were available and functional in 83.3% facilities (mean % of tracer items available 96.0%). Only 46% facilities had access to all eight listed infection control precautions; sharps and infectious waste disposal, and disinfectant availability, were limiting factors (mean % of tracer items available 89.3%). Only 14% had haemoglobin testing and 59% had HIV diagnostic capacity. The mean of the facility-level general readiness score (mean % of tracer items in each of the four domains) in The Gambia was 72.5%.

In Zimbabwe, 19.9% of facilities had all basic amenities (mean % of tracer items available 79.9%). Regular electricity supply was limited to just 36% facilities (ranging from availability in 10% of regional/provincial hospitals to 70% of private hospitals). Of all facilities, 83.3% had all basic equipment items available (mean % of tracer items available 96%) and 57.0% had access to all infection control precautions (mean % of tracer items available 94.3%). Again, safe sharps and infectious waste disposal was a limiting factor, being unavailable in > 30% of facilities (including 80% of central hospitals). Almost all facilities had HIV diagnostic capacity (99%) and 53% had haemoglobin testing. The overall mean readiness score in Zimbabwe was 86.6%.

#### General readiness to provide fracture care in The Gambia

In The Gambia, the median number of adult non-maternity beds per facility was eight (range 0–456) and T&O beds was 0 (range 0–30) (**Table 2**; Table S3 in the **Online Supplementary Document**). The number of doctors working at each facility ranged from none to 196. All central hospitals and 92.3% of private hospitals had doctors, but overall, only 21.4% of public facilities and 46.2% of other facilities (*e.g.* NGO/mission,) had a doctor. Overall, 67.3% of facilities serving urban populations had doctors, whereas just 17.0% of facilities serving rural populations did (Table S4 in the **Online Supplementary Document**). Just 6.1% of public, 69.2% of private and 15.4% of other facilities had general, trauma or orthopaedic surgeons (range across facilities 0–7 surgeons) (**Table 2**; Table S3 in the **Online Supplementary Document**). Almost all facilities had nurses (96.7%, range 0–258 per facility). Around a third of regional, central and private hospitals had at least one physiotherapist, but few other types of facility had a physiotherapist. In The Gambia, 31 (20.7%) facilities could provide surgical services (Table S2 in the **Online Supplementary Document**). Of these, 58.1% had guidelines for Integrated management for emergency and surgical care (IMEESC) and 38.7% had IMEESC staff trained.

**Table 2:** Fracture service readiness – all facilities in The Gambia and Zimbabwe

Variables	The Gambia				Zimbabwe			
	Public*	**Private**†	**Other**‡	**Total**	Public*	**Private**†	**Faith-based**	**Total**
**Fracture service-specific indicators**								
**Number of inpatient beds – MDN (range)**								
Adult, non-maternity beds per facility	9 (0–456)	13 (0–40)	6 (0–42)	8 (0–456)	41 (0–1200)	59 (20–146)	59 (4–350)	50 (0-1200)
T&O beds per facility	0 (0–30)	0 (0–12)	0 (0–14)	0 (0–30)	0 (0–92)	7.5 (0–27)	0 (0–30)	0 (0-92)
**Staff§**								
Total doctors per facility – MDN (range)	0 (0–196.1)	4.5 (0–16.9)	0 (0–16.9)	0 (0–196.1)	1.8 (0–285.3)	1.8 (0–315.5)	1.8 (0–79.8)	1.8 (0-315.5)
Facilities with doctors, %	21.4	92.3	46.2	34.0	62.1	60.0	78.3	67.2
General or T&O Surgeons per facility – MDN (range)	0 (0–7.0)	1.3 (0–2.6)	0 (0–1.8)	0 (0–7.0)	0 (0–4.9)	3.7 (0–43.1)	0 (0–10.5)	0 (0-43.1)
Facilities with general or T&O surgeons, %	6.1	69.2	15.4	14.0	15.5	40.0	5.0	13.4
General or T&O Nurses per facility – MDN (range)	2.9 (0–257.5)	4.9 (0–27.5)	2.9 (0–24.6)	2.9 (0–257.5)	35.5 (0–1137.9)	44 (1–147.9)	17.5 (2–90.9)	27 (0–1137.9)
Facilities with general or T&O nurses, %	96.9	92.3	97.4	96.7	99.1	100.0	100.0	99.5
Physiotherapists per facility – MDN (range)	0 (0–8.8)	0 (0–1.0)	0 (0–1.0)	0 (0–8.8)	0 (0–18.0)	1 (0–6.0)	0 (0–9.0)	0 (0-18.0)
Facilities with physiotherapists, %	4.1	30.8	5.1	6.7	28.4	60.0	20.0	27.4
**Imaging¶, n (%)**								
Digital/non-digital radiography	3 (3.1)	5 (38.5)	2 (5.1)	10 (6.7)	32 (27.6)	6 (60.0)	18 (30.0)	56 (30.1)
MRI (magnetic resonance imaging)	1 (1.0)	0 (0.0)	0 (0.0)	1 (0.7)	0 (0.0)	1 (10.0)	1 (1.7)	2 (1.1)
CT scan (computer tomography)	0 (0.0)	0 (0.0)	0 (0.0)	0 (0.0)	4 (3.4)	4 (40.0)	1 (1.7)	9 (4.8)
**Fracture treatment, n (%)**								
Splints (for limb fractures)	22 (22.4)	8 (61.5)	8 (20.5)	38 (25.3)	69 (59.5)	9 (90.0)	39 (65.0)	117 (62.9)
Slings (for arm fractures)	33 (33.7)	7 (53.8)	11 (28.2)	51 (34.0)	73 (62.9)	10 (100.0)	44 (73.3)	127 (68.3)
Plaster of Paris	15 (15.3)	8 (61.5)	6 (15.4)	29 (19.3)	59 (50.9)	10 (100.0)	43 (71.7)	112 (60.2)
Lower limb traction	7 (7.1)	3 (23.1)	2 (5.1)	12 (8.0)	1 (0.9)	0 (0.0)	1 (1.7)	2 (1.1)
Walking aids (*e.g.* crutches)	22 (22.4)	8 (61.5)	20 (51.3)	50 (33.3)	60 (51.7)	10 (100.0)	42 (70.0)	112 (60.2)
None of those available	36 (36.7)	2 (15.4)	13 (33.3)	51 (34.0)	23 (19.8)	0 (0.0)	7 (11.7)	30 (16.1)
**Medicines║, n (%)**								
Paracetamol	97 (99.0)	13 (100.0)	38 (97.4)	148 (98.7)	85 (73.3)	10 (100.0)	55 (91.7)	150 (80.6)
Codeine	6 (6.1)	6 (46.2)	10 (25.6)	22 (14.7)	13 (11.2)	9 (90.0)	9 (15.0)	31 (16.7)
Morphine	11 (11.2)	5 (38.5)	8 (20.5)	24 (16.0)	31 (26.7)	10 (100.0)	13 (21.7)	54 (29.0)
Oral alendronate	5 (5.1)	1 (7.7)	1 (2.6)	7 (4.7)	3 (2.6)	5 (50.0)	1 (1.7)	9 (4.8)
IV zoledronate	2 (2.0)	0 (0.0)	2 (5.1)	4 (2.7)	2 (1.7)	4 (40.0)	0 (0.0)	6 (3.2)
Calcium supplement tablets	19 (19.4)	8 (61.5)	10 (25.6)	37 (24.7)	12 (10.3)	8 (80.0)	8 (13.3)	28 (15.1)
Vitamin D supplements	18 (18.4)	8 (61.5)	13 (33.3)	39 (26.0)	14 (12.1)	8 (80.0)	8 (13.3)	30 (16.1)
Normal saline IV solution	90 (91.8)	12 (92.3)	34 (87.2)	136 (90.7)	107 (92.2)	10 (100.0)	57 (95.0)	174 (93.5)
Ringers lactate/Hartmann's IV solution	61 (62.2)	10 (76.9)	29 (74.4)	100 (66.7)	109 (94.0)	10 (100.0)	59 (98.3)	178 (95.7)
5% dextrose IV solution	76 (77.6)	11 (84.6)	29 (74.4)	116 (77.3)	90 (77.6)	10 (100.0)	46 (76.7)	146 (78.5)
Skin disinfectant	85 (86.7)	12 (92.3)	30 (76.9)	127 (84.7)	111 (95.7)	9 (90.0)	55 (91.7)	175 (94.1)
Gowns	78 (79.6)	12 (92.3)	29 (74.4)	119 (79.3)	115 (99.1)	10 (100.0)	60 (100.0)	185 (99.5)
Eye protection (goggles or face shields)	50 (51.0)	10 (76.9)	19 (48.7)	79 (52.7)	113 (97.4)	10 (100.0)	58 (96.7)	181 (97.3)
Medical (surgical or procedural) masks	79 (80.6)	10 (76.9)	27 (69.2)	116 (77.3)	112 (96.6)	10 (100.0)	60 (100.0)	182 (97.8)
Absorbable suture material	81 (82.7)	11 (84.6)	29 (74.4)	121 (80.7)	102 (87.9)	10 (100.0)	54 (90.0)	166 (89.2)
Non-absorbable suture material	85 (86.7)	12 (92.3)	28 (71.8)	125 (83.3)	95 (81.9)	10 (100.0)	52 (86.7)	157 (84.4)

IV – intravenous, MDN – median, T&O – trauma and orthopaedic

*Governmental.

†For profit.

‡Non-governmental organisation/faith-based/service/research

§Adjusted for part time workers.

¶Available and functional and there is a staff member trained/qualified to use it.

^║^At least one available and in date.

Only 10 facilities (and fewer than half of regional/provincial, central and private hospitals) had functional radiology facilities with staff trained and qualified in their use. One central hospital had a functional magnetic resonance imaging (MRI) scanner and one a functional computed tomography (CT) scanner (**Table 2**; Table S3 in the **Online Supplementary Document**). Sutures, skin disinfectant, and lidocaine were widely available, but anaesthetic drugs (halothane, bupivacaine), epinephrine and ephedrine were only available in approximately 50% of facilities (Table S2 in the **Online Supplementary Document)**; 45.2% had splints and 58.1% plaster cast materials; with no clear differences between facilities providing for urban and rural populations (Table S4 in the **Online Supplementary Document**). Lower limb traction was available in only 8% of facilities (Table S3 in the **Online Supplementary Document**). Overall, splints, slings, plaster of Paris, lower limb traction and walking aids were not widely available (34% had none of these); availability was best in private facilities (**Table 2**).

Only 35.5% reported an uninterrupted oxygen supply over the preceding three months (Table S2 in the **Online Supplementary Document)**. Most facilities (> 75%) had paracetamol, IV saline, IV dextrose, skin disinfectant, and suture material, available and in date (**Table 2**; Table S3 in the **Online Supplementary Document**). Three quarters of facilities has gowns and masks available, and around half had eye protection.Blood transfusion was possible in 14.0% facilities (66.7% central and 61.5% private hospitals) (Table S2 in the **Online Supplementary Document)**. Of these, 57.1% reported an uninterrupted blood supply over the preceding three months, and 95.2% reported blood supply safety (blood obtained only from national or regional blood bank, or blood screened for HIV, syphilis, hepatitis B and C). Codeine and morphine were not commonly stocked (available in approximately 15% facilities) (**Table 2**; Table S3 in the **Online Supplementary Document**). Calcium and vitamin D supplements were available in 25.6% and 33.3% facilities, while oral/IV bisphosphonates were rare (< 5% facilities).

#### General readiness to provide fracture care in Zimbabwe

In Zimbabwe, the median number of adult non-maternity beds per facility was 50 (range 0–1200), most facilities had no T&O beds (range 0–92) (**Table 2**; Table S3 in the **Online Supplementary Document**). Facilities had median 1.8 doctors per facility (range 0–316). All regional/provincial and central facilities had doctors, as did 56.4% rural/district hospitals, 60% private and 78.3% faith-based facilities. Overall, 92.9% of facilities serving urban populations had doctors, whereas 54.7% of facilities serving rural populations did (Table S4 in the **Online Supplementary Document**). Surgeons were available at 100% central, 70% regional/provincial and 40% private facilities (range 0–43 per facility) (**Table 2**; Table S3 in the **Online Supplementary Document**). Almost all facilities had a nurse (99.5%), ranging from none to 1138 per facility. All central, 80% regional/provincial, 60% private, and 20% rural/district and faith-based facilities, had at least one physiotherapist. In Zimbabwe 129 (69.4%) facilities could provide surgical services (100% of regional/provincial, central, and private facilities) (Table S2 in the **Online Supplementary Document**). Of these, 41.1% had IMEESC guidelines and 22.5% IMEESC trained staff.

Overall, 56 facilities (almost all regional/provincial and central, and 60% private hospitals) had functional X-ray facilities; functional X-ray facilities were more common in facilities providing for urban than rural populations (**Table 2**; Table S4 in the **Online Supplementary Document**). One public facility had a functional MRI scanner, as did one private and one faith-based hospital. Nine facilities reported functional CT scanners. Paracetamol, IV fluids, skin disinfectant, suture material, gowns, masks, and eye protection were widely available. Lidocaine, anaesthetic drugs (halothane, bupivacaine) and epinephrine were available in most facilities, (but ephedrine in only 45.7%) (Table S2 in the **Online Supplementary Document**. Splints, slings, plaster of Paris and walking aids were available in > 60% of facilities (69.8% splints, 78.3% plaster materials), with high availability in central, regional/provincial, and private facilities (**Table 2**; Table S3 in the **Online Supplementary Document**); with no clear differences between facilities providing for urban and rural populations (Table S4 in the **Online Supplementary Document**). Lower limb traction was available in 45.7% of facilities (> 90% regional/provincial, central or private facilities).

Overall, half reported an uninterrupted oxygen supply over the preceding three months (100% central and private facilities) (Table S2 in the **Online Supplementary Document)**. Overall, 106 (57.0%) offered blood transfusion (100% of regional/provincial, central, and private facilities), and half reported an uninterrupted blood supply over the preceding three months (all central hospitals and 60% of private facilities). There was almost universal blood supply safety (99.1%). Codeine and morphine were not commonly available (16.7% and 29% of facilities, respectively) (**Table 2**; Table S3 in the **Online Supplementary Document**). Only 15% of facilities had calcium and vitamin D supplements available. As in The Gambia, bisphosphonates were rarely available (alendronate in 4.8% facilities, zoledronate 3.2%).

#### Service-specific readiness to provide hip fracture care in The Gambia

Ten facilities could diagnose hip fractures (five private) and six could treat them (three routinely applied traction and four could offer surgery) (**Table 3****;** Table S5 in the **Online Supplementary Document**). All ten provided a 24/7 service with eight having a general surgeon and six an orthopaedic surgeon on-call. Guidelines were available for pain management in nine facilities, diagnostic investigation in eight, onward referral in seven and osteoporosis management in three. Facilities reported median 6.5 (range 0–100) hip fracture presentations annually (77.5% estimated to be low impact (fragility) fractures), totalling 177 hip fractures across these 10 facilities each year.

**Table 3:** Hip fracture-specific readiness in facilities that can diagnose and/or treat a hip fracture in The Gambia and Zimbabwe*

Variables	The Gambia	Zimbabwe
**Facilities that can diagnose/treat a hip fracture, n (%)**		
Rural/district hospital	1 (12.5) / 0 (0.0)	44 (43.6) / 31 (30.7)
Regional/provincial hospital	0 (0.0) / 0 (0.0)	10 (100.0) / 9 (90.0)
Central hospital	1 (33.3) / 1 (33.3)	5 (100.0) / 5 (100.0)
Private	5 (38.5) / 4 (30.8)	10 (100.0) / 10 (100.0)
Other†	3 (7.7) / 1 (2.6)	38 (63.3) / 26 (43.3)
Total	10 (15.2) / 6 (9.1)	107 (57.5) / 81 (43.5)
**Of those facilities that diagnose or treat a hip fracture:**		
Facility provides surgical treatment, n (%)	4 (40.0)	17 (15.9)
Traction (skin or skeletal) is routinely applied to injured leg, n (%)	3 (30.0)	79 (73.8)
No. hip fracture presentations (annual), per facility, median (range)	6.5 (0–100)	10 (0–580)
Of these, % low impact (fragility) fractures, median (range)	77.5 (0–100)	62.5 (0–100)
Total number of hip fractures across all facilities	171	2235
**Radiological imaging offered, n (%)**		
Anterior-posterior radiograph of the pelvis	9 (90.0)	63 (58.9)
Anterior-posterior radiograph of the hip	6 (60.0)	71 (66.4)
Lateral radiograph of the hip	7 (70.0)	44 (41.1)

*No health centres/clinics were included because they could not diagnose or treat a hip fracture.

†Non-governmental organisation/faith-based/service/research.

Approximately 15% were thought to be referred in from another facility, 60% having engaged a TBS (Table S5 in the **Online Supplementary Document**). Most patients arrived via private or public transport (not ambulance) and took a median of 24 hours (and up to 30 days) to reach the facility. Most (60%) had accessed simple painkillers such as paracetamol before arrival, 80% of facilities offered patients simple oral analgesia on arrival, 80% intramuscular opiates, 40% IV opiates, and 20% oral opiates. Anterior-posterior pelvic radiographs were performed by nine of the 10 facilities when hip fracture was suspected. Waiting times from arrival to admission ranged 0–48 hours (median one hour), and time from arrival to onward referral from 0–6 hours (median 0.5 hours).

Four facilities had orthopaedic surgeons and operating facilities to treat hip fractures surgically (one central, two private and one NGO) (Table S6 in the **Online Supplementary Document**). Data on the quality of hip fracture services were provided by orthopaedic surgeons, head nurses, and clinical administrators. In these four facilities, median 58% (range = 25–75%) patients per facility were estimated to undergo surgery, with median time from presentation to surgery reported as eight days (interquartile range (IQR) = 2.5–30.5; range = 2–48). The most important factors influencing operative decision making (mean score (x̄) out of 10 shown in brackets) were availability of surgical (x̄ = 9.5) and anaesthetic (x̄ = 9.5) expertise and equipment (x̄ = 8.5), as well as patient health (x̄ = 8.8), patient wishes (x̄ = 8.2), family wishes (x̄ = 7.5) and the ability of the patient/ their family to pay for an operation (x̄ = 7.8). Patient age was not considered an important factor by surgeons (x̄ = 5.2). Receipt of alternative treatment was a factor considered by one facility. Spinal anaesthesia was more widely used than general anaesthesia (regional nerve blocks not routinely used), administered by a qualified anaesthetist or nurse anaesthetist. Cannulated screws and sliding hip screws were available in hospital stock in all four facilities, total hip replacement (THR) and hemi-arthroplasties in three facilities, and intramedullary (IM) nails in two. The preferred fixation for an undisplaced intracapsular fracture was cemented hemi-arthroplasty (two facilities) or cannulated screw fixation (one facility), whilst for a displaced intracapsular fracture cemented hemi-arthroplasty was preferred (three facilities). Two facilities routinely offered cemented THR to patients with good pre-fracture mobility. Extracapsular fractures were generally managed by sliding hip screws, with IM nails in some circumstances. Post-surgical complications were reported by surgical teams: median for pressure sores 2.5%, venous thromboembolism (VTE) 8%, wound infection 1.5%, other infections 3.5%. Two facilities routinely assessed cognition (using the 4 A’s Test [22]).

Notably, an estimated mean of only 43.8% patients across facilities were allowed to weight-bear in the first 24 hours after surgery (range = 0–95%), those receiving internal fixation for an intracapsular fracture waited mean 5.8 days (but ranged 0–21 days across facilities), and for an extracapsular fracture patients waited mean 1.2 days (range = 0–2 days). Patients were mobilised by physiotherapists, orthopaedic nurses, general nurses and/or occupational therapists. Walking frames were available in all four facilities, crutches in three, wheelchairs in three and walking sticks in two. Three facilities could provide a walking frame at discharge at no cost to the patient. The mean estimated average length of stay across facilities was 3.2 days in those hrtoperated, and 1.2 days in those not operated. No dual-energy X-ray absorptiometry (DXA) scanner was available at any facility for clinical purposes. Patients with osteoporosis could be discharged with calcium and vitamin D supplements, but not bisphosphonates, hormone replacement therapy (HRT), or other osteoporosis medication. Only one facility reported auditing patients post-discharge (for readmission and reoperation).

#### Service-specific readiness to provide hip fracture care in Zimbabwe

In Zimbabwe, 107 facilities reported being able to diagnose a hip fracture, including all central, regional/provincial, and private hospitals (**Table 3****;** Table S5 in the **Online Supplementary Document**), whilst 81 (43.5%) could treat hip fractures (90% of regional/provincial and all central and private hospitals) – 73.8% routinely applied traction, whilst only 15.9% could provide surgery. Most provided a 24 / 7 service, with 38.3% having a general surgeon and 19.6% an orthopaedic surgeon on-call. Guidelines were available for pain management in 39.3%, diagnostic investigation in 37.4%, onward referral in 46.7% and osteoporosis management in 24.3%. Facilities reported median 10 (range = 0–580) hip fracture presentations annually (62.5% estimated to be fragility fractures), totalling 2235 across these 107 facilities each year. Around 40% were thought to be referred in from another facility (Table S5 in the **Online Supplementary Document**). Patients arrived via ambulance, private and public transport and animal drawn vehicles, and the estimated median time to facility from injury was six hours (range = 0–168 hours), during which 88.8% were likely to have accessed simple painkillers, and fewer than 10% opiates. At 79.4% facilities, patients were offered simple oral analgesia, 69.2% intramuscular opiates, 35.5% oral opiates and 15.9% IV opiates. Antero-posterior pelvic radiographs were performed by 58.9% of facilities when hip fracture was suspected. Waiting times from arrival to admission ranged 0–12 hours (MDN = 1 hour), time for onward referrals ranged from 0–24 hours (MDN = 2 hours).

In total 17 facilities had access to orthopaedic surgeons and operating facilities to treat hip fractures surgically (nine public, seven private and one faith-based facility – the latter grouped with private in Table S6 in the **Online Supplementary Document**). Data on the quality of hip fracture services were largely provided by orthopaedic surgeons, as well as district medical officers and medical superintendents, head matrons and orthopaedic nurses. On average, 75% (range = 4–100%) patients in public facilities, and 99% (range = 90–100%) in private facilities were estimated to undergo surgery, with time from presentation to surgery reported as MDN = 14 days (IQR = 7–21; range = 0–60) in the nine public and two days (IQR = 1–3; range = 1–3) in the eight private facilities. In public facilities, the most important factors in deciding whether to operate (mean scores out of 10 shown) were: availability of equipment (x̄ = 9.7), surgical (x̄ = 10) and anaesthetic expertise (x̄ = 10.0), patient’s health (x̄ = 9.6) and wishes (x̄ = 8.7). Patient age (x̄ = 9.2), family wishes (x̄ = 9.0), and the patient’s ability to pay for surgery (x̄ = 10.0) were also considered important in private facilities. Spinal anaesthesia was most commonly used (median 70%), then general anaesthesia (median 30%) (regional nerve blocks not routinely used), given by a qualified anaesthetist, or rarely an anaesthetist in training or a nurse anaesthetist. Two-thrids (66.7%) of public facilities stocked hemi-arthroplasties and 44.4% IM nails. Cannulated screws, sliding hip screws and THRs were not widely stocked. Most private facilities (62.5%) did not stock any surgical implants. There was a wide variety in the preferred surgical option for intracapsular fracture fixation (both displaced and undisplaced) across facilities. Some reported patient age and health influence surgical preference. Most facilities offered THR to patients with good mobility before hip fracture. Estimates of post-operative complication rates varied widely in public facilities, but were, on average, higher than private facilities (*e.g.* estimated VTE in 14% of patients in public and 2% in private facilities, pressure sores in 10% of public patients, *vs.* 1% in private patients, and post-operative wound infections in an estimated 5% of public facility patients). Cognition was routinely assessed in fewer than half of facilities (commonly by an abbreviated mental test).

Across both public and private facilities, a mean of only 33% of patients were permitted to weight-bear in the first 24 hours post-surgery, though practice varied greatly. Notably, post-operative non-weight-bearing periods were 2–3 times longer in public than private facilities, irrespective of surgical fixation. Public facilities reported routinely mobilising on average six days after surgery, as opposed to 1.1 days in private facilities. Physiotherapists, orthopaedic nurses, general nurses and/or occupational therapists helped mobilise patients. Most facilities had crutches, walking frames and wheelchairs available. Only one private facility had a hoist. None provided free mobility aids at discharge. Crutches, walking frames, walking sticks, and wheelchairs were sometimes available at a cost to the patient. The mean estimated length of stay across public facilities was 23.6 days (range = 5–60) in those operated, and 35.8 days (range = 7–90) in those not operated. In private facilities, estimates were 5.2 days (range = 3–7) in those operated, and 17.5 days (range = 0–42) in those not operated.

No public facility reported access to DXA for clinical use. Some reported using The Fracture Risk Assessment Tool and/or bone turnover markers. In private facilities, most (62.5%) reported using DXA, though only one facility had a DXA available. Patients with osteoporosis would mainly be discharged with calcium and vitamin D supplements. Two public facilities reported prescribing alendronate, one zoledronate and two HRT. Four private facilities reported prescribing zoledronate, and one HRT. Approximately 41% of facilities collected patient data following discharge.

#### Traditional bone-setters in The Gambia

**Figure 3:**
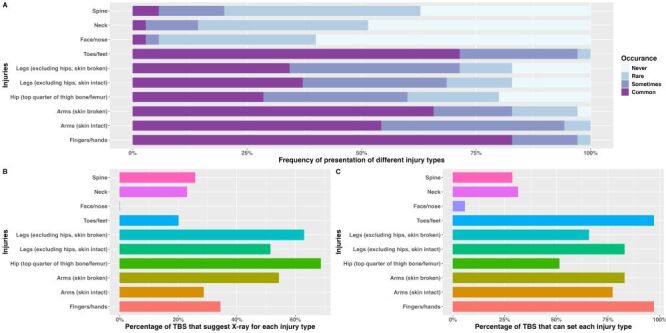
Traditional bone-setter care by type of injury. **Panel A.** Frequency of presentation of different injury types. **Panel B.** Percentage of TBS that suggested X-ray for each injury type. **Panel C.** Percentage of TBS that can set each injury type. TBS – traditional bone-setter.

Initially 53 TBS were potentially identified using a register obtained from regional health teams and by word-of-mouth, of whom 11 either did not exist, were duplicates or non-operational (Figure S1 in the **Online Supplementary Document**). Of the 42 who were confirmed to exist, one (2.4%) registered urban-based TBS declined due to cultural reasons and six (14.3%) could not be contacted (all non-registered, three were travelling TBS from other countries, three rural TBS could not be contacted), therefore 35 (83.3%) TBS participated. Most (91.4%) data were collected face-to-face, the remainder by phone. Most (74.3%) TBS surveyed practiced in the West Coast region, 65.7% serving urban/peri-urban populations (Table S7 in the **Online Supplementary Document**). Many (91.4%) were trained by a TBS in their family, although 5.7% trained themselves; one was trained by another local TBS. The TBS had worked for on average 26.1 years (range = 2–71 years), they worked with mean 1.7 (range = 0–3) other persons (mainly assistants, but some were other TBS or trainee TBS). Traditional bone-setters often worked full time (68.6%), 91.4% working from home, 28.6% had overnight beds available for patients to stay. Injuries of fingers and hands, toes and feet and arms were considered common, injuries to legs and hips less so (**Figure 3**, Panels A–C). Neck and spine injuries were rarely seen. Over half of TBS recommend getting a radiograph for a suspected arm (if open), hip, or leg fracture. Of all injury types, 97.1% said they could set fingers, hands, toes and feet, most said they could set arm or leg fractures, 51.4% said they could set hip fractures, and 31.4% said they could set a neck or spine fracture. Traditional medication or herbs were widely used as analgesia. Paracetamol and non-steroidal anti-inflammatories were sometimes used. For fractures the TBS are unable to set, 51.4% refer to a clinical facility and 37.1% to another TBS. Most TBS (88.6%) did not keep records of fractures managed.

For hip fractures, the most common treatment was herbal or traditional medicine (used by 57.1% TBS), followed by splinting (20.0%), manipulation (14.3%) and traction (5.7%). Overall, 31.4% said they would not treat a hip fracture and 14.3% would refer to a hospital. The 35 TBS estimated they had seen, in the last year, between 0–350 fractures (MDN = 25), and between 0–30 hip fractures (MDN = 0). In the previous year, most TBS (77.1%) had not referred any hip fracture cases for X-ray nor to a hospital or clinic. Of the TBS, 31.4% reported having splints available for fracture treatment, 28.6% had plaster of Paris, 22.9% had walking sticks, 20.0% slings, 5.7% traction and 5.7% crutches. One TBS had a wheelchair. Three quarters of TBS facilities had electricity, 88.6% had a light source available, 97.1% a mobile phone (no landlines), only one had internet for email (most communicate with patients and families via WhatsApp). Only a quarter had clean running water, 8.6% had a flush toilet. After a hip injury, patients usually took > two days to present to TBS; 71.4% TBS visit patients (by motorbike, taxi, and bicycle). Medical waste disposal was variable, commonly by storage or burning. Processing of equipment for reuse (*e.g.* washing in boiling water) was not possible in 80% of facilities. Handwashing soap was available in 34.4%, no facilities had alcohol hand rub, latex gloves, a pedal bin with liners, a sharps container or environmental disinfectant.

#### Repeatability and data collection

Inter-rater agreement was good across multiple key variables; 86.1–97.2% for medical facility data (n = 18 facilities in The Gambia; n = 19 in Zimbabwe), 80–100% for TBS data (n = 5 TBS) (Table S8 in the **Online Supplementary Document**). Although repeatability scores were high on the variables assessed, extensive internal consistency checks indicated some questions had not been well understood. In these cases, verification was sought first from the fieldworker, and then from the facility if the fieldworker was uncertain. These facility queries were largely conducted face-to-face in The Gambia and over the phone in Zimbabwe.

## DISCUSSION

This study represents the first service availability and readiness assessment specifically of fracture services in Africa, spanning 186 facilities in Zimbabwe and 150 in The Gambia, where 35 TBSs were also assessed. In both countries most facilities were serving rural populations, and these lacked surgical capacity. Similarly low adult inpatient bed availability was identified in both Zimbabwe and in The Gambia, with approximately 7% dedicated to orthopaedic trauma.

General availability of hospital facilities was low in both Zimbabwe and The Gambia where 2.2 and 12 facilities per 100 000 adults were identified respectively [8]. Many facilities lacked regular electricity and reliable oxygen supplies, and sharp and infectious waste disposal. In The Gambia, most public hospitals and more than half of other facility types (*e.g.* NGO/mission facilities) had no doctors of any type. This inadequate level of provision aligns with a 2016 SARA assessment of general surgical capacity (rather than orthopaedic trauma care) across eight African countries (not including Zimbabwe or The Gambia) [8]. The well-documented and chronic ‘brain drain’ of medical professionals from African countries to the global north will be an exacerbating factor [23]. Perhaps surprisingly, despite the recent COVID-19 pandemic, basic items for infection prevention and control measures were widely unavailable. It is notable that in Zimbabwe, where HIV care is well-funded by external donors and NGOs, that HIV diagnostic capacity was available in 99% of facilities.

In terms of readiness to provide fracture care, fewer than one orthopaedic surgeon was available for 100 000 adults in both countries; even lower than the 1.63 per 100 000 identified in South Africa in 2017 [24]. Orthopaedic trained nurses, physiotherapists and occupational therapists were equally few. Only 10 facilities in The Gambia and 56 in Zimbabwe had functioning X-ray facilities. Equipment for fracture immobilisation was not widely available in either setting, and most lacked access to lower limb traction. In both countries DXA scanning was unavailable in public facilities, and access to antiresorptive treatment, to reduce future fracture risk, was limited to fewer than 5% of facilities.

Focusing on hip fracture readiness specifically, this current assessment highlighted very low provision for all cadres of healthcare staff that constitute a hip fracture multidisciplinary team. Non-operative management was common in Zimbabwe and those visiting TBS in The Gambia. In those undergoing an operation, inpatient delays waiting for surgery were long, especially in Zimbabwe at an estimated two weeks, potentially reflecting a patient’s ability to pay. Surgical practice was variable, for example post-operative weight-bearing was frequently restricted, particularly in Zimbabwean public hospitals, where, in addition, access to walking aids was severely limited. Potentially linked was the reported 14% VTE complication rate and 10% pressure sore rate in public hospitals in Zimbabwe; estimates that require validation. Formal linkage of these service-level organisational factors with patient-level fracture outcomes was not possible as patient-level data were not collected, and routinely collected health statistics are not available in either country. However, in an ongoing study in both settings, patient outcomes by private *vs*. public facility care provision will be able to be determined [13]. Fracture care was highly pluralistic in The Gambia, where most TBS felt they could ‘set’ hip fractures. Notably, infection prevention and control measures were absent from TBS practice.

Pluralistic fracture care is seen in many parts of Africa, especially where allopathic care is costly and geographically remote. This study documented evidence of integration of traditional and allopathic services – 51% of TBS were willing to refer patients to a hospital for fractures they felt unable to manage. Splinting and traction are employed by both traditional and allopathic practitioners; however, whether techniques were equally effective could not be evaluated. This study identified, as other studies have in Tanzania, Ethiopia, Chad and Nigeria [25,26] that some TBS practices raise concern regarding infection, malunion and iatrogenic trauma from manipulation. Yet, as has been suggested in Tanzania, formalising a respectful working relationship between TBS and orthopaedic surgeons, could enable co-development of shared care protocols to optimise access to orthopaedic care to those triaged as particularly needing allopathic care, such as hip fractures [25].

Whilst medical aid is available in Zimbabwe, most citizens do not have cover and hence, when faced with a hip fracture, families need to suddenly access often catastrophic costs to permit operative management [27], potentially contributing to delays and barriers to surgery. Currently, The Gambia is planning to introduce a national health insurance, whether this improves access to fracture services will need to be determined. However, in both settings, the relative lack of orthopaedic surgeons, allied health professionals, inpatient beds, surgical equipment, and prostheses suggests other policy and infrastructural changes are needed. Potential improvements could address supply chains for medical equipment, guideline development and staff training, standardisation of surgical practices, immediate post-operative weight-bearing to aid mobility recovery, task shifting such as nurse-lead mobilisation, and inclusion of low-cost anti-osteoporosis medicines on essential medicines lists.

The limitations to care delivery outlined in these two countries reflect the increasingly recognised challenges to hip fracture care provision seen in low-income settings across the world [28]. The SARA data presented here, as well as similar assessments of care provision in Southeast Asia, go some way to explain the observed challenges in delivering timely care such as prompt surgery and hospital admission [29,30].

Study strengths included a very high response rate such that all facilities across Zimbabwe and 98% across The Gambia were included, maximising generalisability. The survey was highly comprehensive, covering service availability and general, fracture, and hip fracture-specific readiness. It is very unusual to include TBS in a study such as this and provides the first systematic assessment of training and practice in The Gambia. The high level of agreement between initial and repeat data collection indicates good repeatability and therefore reliable results. Study limitations included one central hospital in The Gambia that can diagnose but not treat hip fractures declining to participate. Although facility managers were the preferred responders to the hospital services questionnaire, and doctors with orthopaedic expertise to the fracture service questionnaire, often nurses, and sometimes administrators, completed the latter due time pressures on doctors, potentially reducing accuracy of orthopaedic-specific data. In both countries many orthopaedic surgeons work full time in public and part time in private facilitates. Although we adjusted for part time workers, the number of surgeons may still have been overestimated. Readiness may be optimistically reported with responses reflecting what should be available, rather than temporary local stock outs and further, staffing and beds numbers are often dynamic. The number of facilities that reported being able to diagnose a hip fracture was more than the number with radiography facilitates, potentially reflecting use of clinical diagnostic criteria or external imaging facilities. Such private radiology facilities were not enumerated. Nor were physician assistants, an important provider of care in The Gambia. As many facilities in both countries provide healthcare services to both urban and rural dwelling people, a formal urban-rural analysis was not possible. When drawing direct comparisons between the countries it should be considered that the inclusion criteria varied slightly for each country, as CHCs were not categorised in Zimbabwe. As there is no register or official records documenting TBS, some TBS were likely to have been missed, particularly in rural areas. Particularly in The Gambia, people could attend more than one TBS/facility so hip fracture estimations may include some double counting.

In conclusion, this is the first study to quantity fracture service provision, with a particular focus on hip fracture care, in The Gambia or Zimbabwe, and provides novel insights into traditional health practices. The findings highlight multiple important deficits in care which warrant urgent focus, given the expected rise in fragility fractures as populations in Africa rapidly age. These data underpin a series of evidence-based recommendations (**Box 1**) which are intended to guide improvement in fracture services in Zimbabwe and The Gambia.

Box 1 **Summary of main recommendations applicable to both The Gambia and Zimbabwe**

Concerning the availability of hospital beds and staff

1. Increase public hospital availability of orthopaedic and trauma beds

2. Investment in training orthopaedic surgeons and anaesthetists

3. Train nurses specifically in orthopaedic care

4. Increase availability of rheumatologists, geriatricians, and radiographers in public hospitals

5. Increase availability of physiotherapists, occupational therapists, hospital pharmacists, and hospital nutritionists

Concerning basic amenities

6. Clinic assessment facilities need to afford privacy to patients

7. Establish uninterrupted electricity and oxygen supplies to facilities

8. Establish sharps and infectious waste disposal systems for all facilities

9. Increase availability of services to perform basic blood tests

Concerning readiness to provide fracture care

10. Increase access to surgical expertise for rural-dwelling communities

11. Increase training and availability of radiographers

12. Maintain basic X-ray equipment so that is functional

13. Ensure all facilities have splints, slings, and plaster of Paris available for immediate fracture care

14. Maintain stocks of basic analgesia in all facilities to which a patient might present with a fracture (and acute pain), with a guideline to aid nurse-led administration of pain relief

15. Ensure an uninterrupted blood supply to all facilities providing trauma care*

16. Maintain stocks of basic anti-osteoporosis treatments to reduce re-fracture risk, in all facilities which manage fragility fractures

17. Make available public access to DXA scanning in key facilities within larger urban centres

Concerning readiness to provide hip fracture care

18. Introduce national guidelines for (i) hip fracture care and (ii) the assessment and management of fall and fracture risk

19. Ensure all facilities to which a patient with hip fracture could present have access to lower limb traction and nurses are trained in its use

20. Hospitals that provide orthopaedic surgery should purchase a stock of basic surgical implants *e.g.* hemiarthroplasties, sliding hip screws, and keep these in stock with a system for cost recovery charging

21. Patients with a fragility fracture should be allowed unrestricted weight bearing on the day of or the day after surgery, with inpatient mobilisation until fit for discharge

22. Make available walking aids in all facilities providing orthopaedic care, to enable early patient mobilisation

Traditional Bone Setters in The Gambia

• Training could be provided to TBS regarding infection prevention and control, and safe disposal of medical waste

• Pathways of care could be co-developed with TBS to streamline hip fracture referrals to hospitals for surgical fixation

*Gambia only, as this is already in place in Zimbabwe

## Additional material


Online Supplementary Document
