# Supplementary material for: Challenges to fracture service availability and readiness provided by allopathic and traditional health providers: national surveys across The Gambia and Zimbabwe
Source: J Glob Health. 2025 Mar 14;15:04082. doi: 10.7189/jogh.15.04082 (PMC11907378; doi:10.7189/jogh.15.04082)
Supplement: Online Supplementary Document [file jogh-15-04082-s001.pdf]

Supplement to: Burton A, Manyanga T, Wilson H, Jarjou L, Costa ML, Graham S, Masters J, Jallow MK, Hawley S, Nyassi MT, Mushayavanhu P, Ndekwere M, Ferrand RA, Ward KA, Marenah KS, Gregson CL. Challenges to fracture service availability and readiness provided by allopathic and traditional health providers: national surveys across The Gambia and Zimbabwe. J Glob Health. 2025;15:04082.

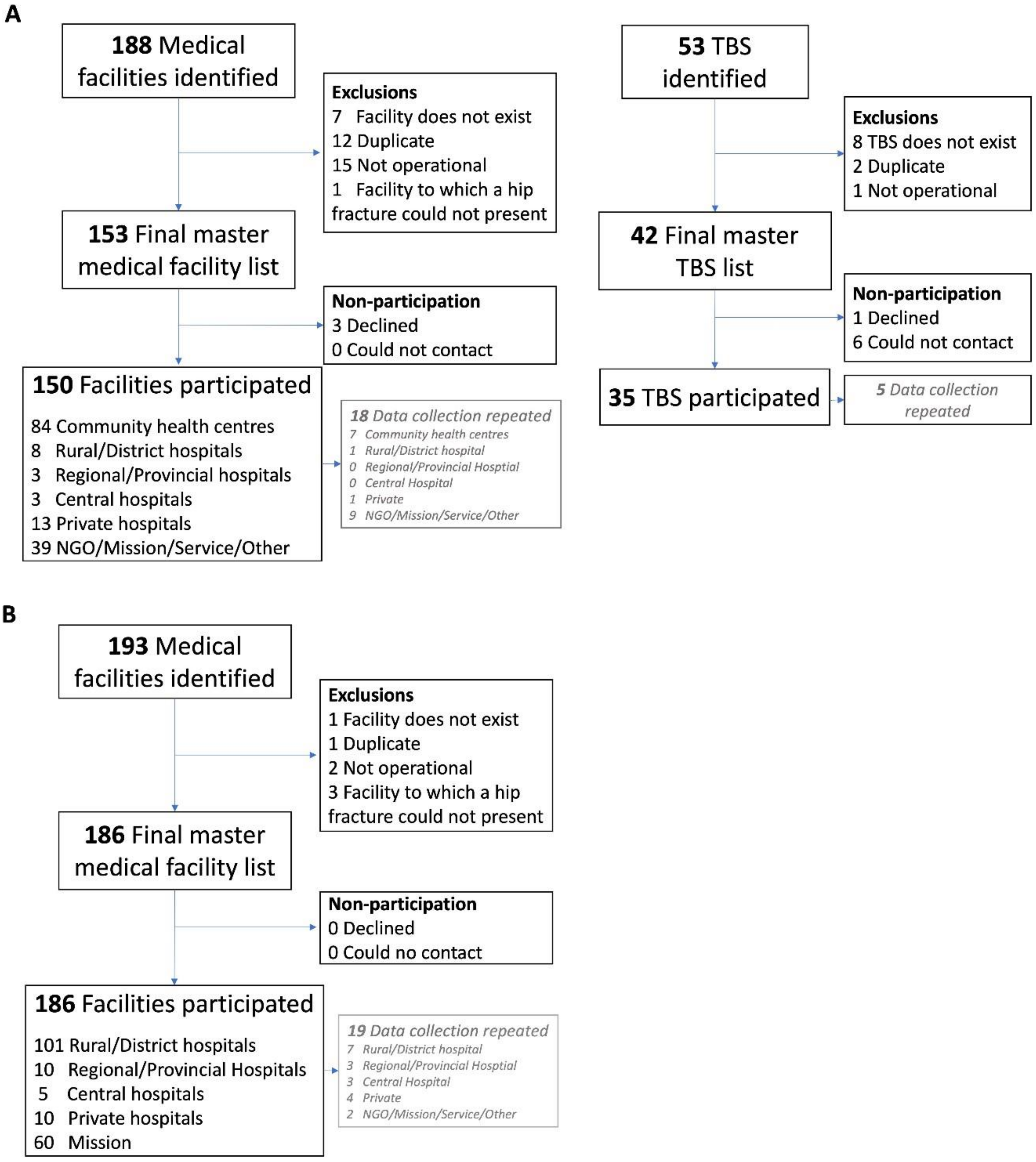

Supplementary Figure 1: Consort diagrams – A) The Gambia, B) Zimbabwe

Supplementary Table 1: Service Availability - staffing

		The Gambia				Zimbabwe			
		Public	Private	Other	Total	Public	Private	Faith-based	Total
Doctors (total)	N	300	95	84	479	1318	682	200	2200
	N weighted	267.4	84.7	74.9	427.0	1198.1	619.9	181.8	1999.8
	Density (per 100,000)	21.5	6.8	6.0	34.3	14.2	7.4	2.2	23.8
General Surgeons	N	9	13	7	29	30	98	13	141
	N weighted	7.9	11.4	6.2	25.5	18.5	60.3	8.0	86.7
	Density (per 100,000)	0.6	0.9	0.5	2.0	0.2	0.7	0.1	1.0
Orthopaedic Surgeons	N	6	5	1	12	27	36	7	70
	N weighted	5.3	4.4	0.9	10.5	16.6	22.1	4.3	43.1
	Density (per 100,000)	0.4	0.4	0.1	0.8	0.2	0.3	0.1	0.5
Anaesthetists	N	13	9	4	26	35	119	7	161
	N weighted	11.4	7.9	3.5	22.8	21.5	73.2	4.3	99.0
	Density (per 100,000)	0.9	0.6	0.3	1.8	0.3	0.9	0.1	1.2
Internal Medicine	N	28	31	15	74	15	57	13	85
	N weighted	24.6	27.2	13.2	65.0	9.1	34.5	7.9	51.4
	Density (per 100,000)	2.0	2.2	1.1	5.2	0.1	0.4	0.1	0.6
Geriatricians	N	0	0	0	0	0	0	0	0
	N weighted	0.0	0.0	0.0	0.0	0.0	0.0	0.0	0.0
	Density (per 100,000)	0.0	0.0	0.0	0.0	0.0	0.0	0.0	0.0
Rheumatologists	N	0	0	0	0	0	2	0	2
	N weighted	0.0	0.0	0.0	0.0	0.0	1.2	0.0	1.2
	Density (per 100,000)	0.0	0.0	0.0	0.0	0.0	0.0	0.0	0.0
Radiologists	N	3	3	2	8	5	7	0	12
	N weighted	2.6	2.6	1.8	7.0	3.0	4.2	0.0	7.3
	Density (per 100,000)	0.2	0.2	0.1	0.6	0.0	0.1	0.0	0.1
Specialist doctors	N	83	41	41	165	146	474	40	660
(ignore)	N weighted	72.9	36.0	36.0	145.0	88.3	286.8	24.2	399.3
	Density (per 100,000)	5.9	2.9	2.9	11.6	1.0	3.4	0.3	4.7
Junior doctors	N	115	44	39	198	333	13	52	398
	N weighted	113.0	43.3	38.3	194.6	332.7	13.0	52.0	397.6
	Density (per 100,000)	9.1	3.5	3.1	15.6	4.0	0.2	0.6	4.7
General Nurses	N	1205	102	178	1485	9800	519	1369	11688
	N weighted	1184.5	100.3	175.0	1459.7	9790.9	518.5	1367.7	11677.1
	Density (per 100,000)	95.0	8.0	14.0	117.1	116.3	6.2	16.3	138.7
Orthopaedic -trained nurses	N	0	4	3	7	25	1	0	26
	N weighted	0.0	3.9	2.9	6.9	25.0	1.0	0.0	26.0
	Density (per 100,000)	0.0	0.3	0.2	0.6	0.3	0.0	0.0	0.3
Nurse aids/attendants	N	783	72	176	1031	2218	258	994	3470
	N weighted	769.7	70.8	173.0	1013.4	2215.9	257.8	993.1	3466.8
	Density (per 100,000)	61.7	5.7	13.9	81.3	26.3	3.1	11.8	41.2
Physiotherapists	N	14	4	2	20	105	13	24	142
	N weighted	13.8	3.9	2.0	19.7	104.9	13.0	24.0	141.9
	Density (per 100,000)	1.1	0.3	0.2	1.6	1.2	0.2	0.3	1.7
Occupational Therapists	N	1	2	0	3	51	3	7	61
	N weighted	1.0	2.0	0.0	2.9	51.0	3.0	7.0	60.9
	Density (per 100,000)	0.1	0.2	0.0	0.2	0.6	0.0	0.1	0.7
Nutritionists	N	1	0	1	2	59	3	16	78
	N weighted	1.0	0.0	1.0	2.0	58.9	3.0	16.0	77.9
	Density (per 100,000)	0.1	0.0	0.1	0.2	0.7	0.0	0.2	0.9
Radiographers	N	8	11	3	22	120	28	18	166
	N weighted	7.9	10.8	2.9	21.6	119.9	28.0	18.0	165.8
	Density (per 100,000)	0.6	0.9	0.2	1.7	1.4	0.3	0.2	2.0
Pharmacists	N	35	24	22	81	85	17	16	118
	N weighted	34.4	23.6	21.6	79.6	84.9	17.0	16.0	117.9
	Density (per 100,000)	2.8	1.9	1.7	6.4	1.0	0.2	0.2	1.4
Social Workers	N	17	5	25	47	20	0	4	24
	N weighted	16.7	4.9	24.6	46.2	20.0	0.0	4.0	24.0
	Density (per 100,000)	1.3	0.4	2.0	3.7	0.2	0.0	0.0	0.3

Supplementary Table 2: General, Surgical and Blood transfusion Service Readiness - All facilities

	The Gambia							Zimbabwe					
	Health centre/clinic	Rural/District Hospital	Regional/Provincial Hospital	Central Hospital	Private	Other	All Facilities	Rural/District Hospital	Regional/Provincial Hospital	Central Hospital	Private	Faith-based	All Facilities
N Facilities	84	8	3	3	13	39	150	101	10	5	10	60	186
**GENERAL READINESS (% of facilities with tracer items, unless otherwise specified)**													
**Basic amenity tracer items**													
Power	53.6%	53.6%	53.6%	53.6%	53.6%	53.6%	60.7%	35.6%	10.0%	60.0%	70.0%	33.3%	36.0%
Improved water source*	100.0%	100.0%	100.0%	100.0%	100.0%	100.0%	100.0%	97.0%	100.0%	100.0%	100.0%	98.3%	97.8%
Room with auditory and visual privacy for patient consultations	27.4%	27.4%	27.4%	27.4%	27.4%	27.4%	32.0%	88.1%	80.0%	100.0%	90.0%	86.7%	87.6%
Access to adequate sanitation facilities for clients	98.8%	98.8%	98.8%	98.8%	98.8%	98.8%	99.3%	99.0%	100.0%	100.0%	100.0%	100.0%	99.5%
Communication equipment (phone or short wave radio)	64.3%	64.3%	64.3%	64.3%	64.3%	64.3%	76.0%	92.1%	100.0%	100.0%	100.0%	86.7%	91.4%
Facility has access to computer with email/internet access	22.6%	22.6%	22.6%	22.6%	22.6%	22.6%	38.0%	71.3%	80.0%	100.0%	100.0%	78.3%	76.3%
Emergency transportation	73.8%	73.8%	73.8%	73.8%	73.8%	73.8%	73.3%	60.4%	100.0%	100.0%	90.0%	76.7%	70.4%
Facilities with all 7 Items - N(%)	1 (1.2%)	1 (12.5%)	0 (0.0%)	0 (0.0%)	6 (42.9%)	4 (10.5%)	12 (8.0%)	18 (17.8%)	1 (10.0%)	3 (60.0%)	6 (60.0%)	9 (15.0%)	37 (19.9%)
Mean % of 7 tracer items available across the basic amenity domain**	62.9%	83.9%	76.2%	76.2%	87.9%	69.6%	68.5%	77.7%	81.4%	94.3%	92.9%	80.0%	79.9%
**Basic equipment tracer items**													
Adult scale	92.9%	100.0%	100.0%	100.0%	100.0%	97.4%	95.3%	93.1%	80.0%	100.0%	100.0%	98.3%	94.6%
Thermometer	98.8%	100.0%	100.0%	100.0%	100.0%	92.3%	97.3%	99.0%	100.0%	100.0%	100.0%	100.0%	99.5%
Stethoscope	98.8%	100.0%	100.0%	100.0%	100.0%	94.9%	98.0%	96.0%	90.0%	80.0%	100.0%	98.3%	96.2%
Blood pressure apparatus	98.8%	100.0%	100.0%	100.0%	100.0%	97.4%	98.7%	93.1%	90.0%	100.0%	100.0%	98.3%	95.2%
Light source	89.3%	100.0%	100.0%	100.0%	92.3%	89.7%	90.7%	92.1%	100.0%	100.0%	100.0%	96.7%	94.6%
Facilities with all 5 Items- N(%)	68 (81.0%)	8 (100.0%)	3 (100.0%)	3 (100.0%)	12 (85.7%)	31 (81.6%)	125 (83.3%)	78 (77.2%)	7 (70.0%)	4 (80.0%)	10 (100.0%)	56 (93.3%)	155 (83.3%)
Mean % of 5 tracer items available across the basic equipment domain**	95.7%	100.0%	100.0%	100.0%	98.5%	94.4%	96.0%	94.7%	92.0%	96.0%	100.0%	98.3%	96.0%
**Infection precautions tracer items**													
Safe final disposal of sharps and infectious waste	75.0%	75.0%	66.7%	100.0%	92.3%	84.6%	79.3%	74.3%	70.0%	20.0%	80.0%	60.0%	68.3%
Appropriate storage of sharps waste	100.0%	100.0%	100.0%	100.0%	92.3%	97.4%	98.7%	99.0%	90.0%	100.0%	100.0%	100.0%	98.9%
Appropriate storage of infectious waste	96.4%	100.0%	100.0%	66.7%	92.3%	87.2%	93.3%	90.1%	90.0%	100.0%	100.0%	93.3%	91.9%
Disinfectant	66.7%	87.5%	100.0%	66.7%	53.8%	71.8%	68.7%	97.0%	100.0%	100.0%	100.0%	96.7%	97.3%
Single use —standard disposable or auto-disable syringes	98.8%	100.0%	100.0%	100.0%	92.3%	92.3%	96.7%	99.0%	100.0%	100.0%	90.0%	100.0%	98.9%
Soap and running water or alcohol based hand rub	98.8%	87.5%	100.0%	100.0%	92.3%	100.0%	98.0%	100.0%	100.0%	100.0%	100.0%	100.0%	100.0%
Latex gloves	98.8%	100.0%	100.0%	100.0%	92.3%	97.4%	98.0%	100.0%	100.0%	100.0%	100.0%	100.0%	100.0%
Guidelines for standard precautions	94.0%	100.0%	100.0%	100.0%	84.6%	79.5%	90.0%	98.0%	100.0%	100.0%	100.0%	100.0%	98.9%

Facilities with all 8 Items- N(%)	43 (51.2%)	4 (50.0%)	2 (66.7%)	2 (66.7%)	5 (38.5%)	16 (41.0%)	72 (48.0%)	60 (59.4%)	5 (50.0%)	1 (20.0%)	8 (80.0%)	32 (53.3%)	106 (57.0%)
Mean % of 8 tracer items available across the infection precaution domain**	91.1%	93.8%	95.8%	91.7%	86.5%	88.8%	90.3%	94.7%	93.8%	90.0%	96.2%	93.8%	94.3%
**Diagnostic capacity tracer items**													
Haemoglobin testing	0.0%	75.0%	33.3%	66.7%	61.5%	10.3%	14.0%	42.6%	90.0%	100.0%	80.0%	56.7%	53.2%
HIV testing	53.6%	100.0%	66.7%	66.7%	69.2%	56.4%	58.7%	100.0%	100.0%	100.0%	90.0%	98.3%	98.9%
Facilities with both Items- N(%)	0 (0.0%)	6 (75.0%)	1 (33.3%)	2 (66.7%)	7 (53.8%)	4 (10.3%)	20 (13.3%)	43 (42.6%)	9 (90.0%)	5 (100.0%)	7 (70.0%)	33 (55.0%)	97 (52.2%)
Mean % of 2 tracer items available across the diagnostic capacity domain**	26.8%	87.5%	50.0%	66.7%	65.4%	33.3%	36.3%	71.3%	95.0%	100.0%	85.0%	77.5%	76.1%
**Overall mean General Readiness Score**	68.9%	91.3%	80.5%	83.6%	83.4%	71.4%	72.5%	84.6%	90.5%	95.1%	93.5%	87.4%	86.6%
**SURGICAL SERVICES (selected tracer items)**													
Facility provides surgical services	6 (7.1%)	5 (62.5%)	1 (33.3%)	2 (66.7%)	9 (69.2%)	8 (20.5%)	31 (20.7%)	60 (59.4%)	10 (100.0%)	5 (100.0%)	10 (100.0%)	44 (73.3%)	129 (69.4%)
**Staff and guidelines**													
Guidelines for IMEESC	66.7%	40.0%	100.0%	50.0%	66.7%	50.0%	58.1%	40.0%	70.0%	80.0%	70.0%	25.0%	41.1%
Staff trained in IMEESC	16.7%	60.0%	100.0%	50.0%	44.4%	25.0%	38.7%	23.3%	20.0%	60.0%	40.0%	13.6%	22.5%
**Equipment**													
Oxygen	0.0%	60.0%	100.0%	100.0%	44.4%	12.5%	35.5%	43.3%	80.0%	100.0%	100.0%	38.6%	51.2%
**Medicines and commodities**													
Skin disinfectant	100.0%	100.0%	100.0%	100.0%	100.0%	87.5%	96.8%	95.0%	100.0%	100.0%	100.0%	97.7%	96.9%
Sutures (any type)	100.0%	100.0%	100.0%	100.0%	100.0%	100.0%	100.0%	90.0%	90.0%	100.0%	100.0%	88.6%	90.7%
Halothane (inhalation)	0.0%	40.0%	100.0%	100.0%	77.8%	50.0%	51.6%	53.3%	90.0%	60.0%	70.0%	50.0%	56.6%
Bupivacaine (injection)	0.0%	80.0%	100.0%	0.0%	88.9%	50.0%	54.8%	70.0%	70.0%	60.0%	90.0%	72.7%	72.1%
Epinephrine (injection)	16.7%	40.0%	100.0%	100.0%	77.8%	37.5%	51.6%	61.7%	80.0%	60.0%	80.0%	70.5%	67.4%
Ephedrine (injection)	33.3%	40.0%	100.0%	100.0%	66.7%	37.5%	51.6%	33.3%	70.0%	60.0%	100.0%	43.2%	45.7%
Lidocaine (1% or 2% injectable)	100.0%	80.0%	100.0%	50.0%	77.8%	87.5%	83.9%	83.3%	70.0%	60.0%	80.0%	72.7%	77.5%
Splints for extremities	16.7%	40.0%	100.0%	50.0%	77.8%	25.0%	45.2%	61.7%	100.0%	80.0%	90.0%	68.2%	69.8%
Material for cast	33.3%	80.0%	100.0%	50.0%	77.8%	37.5%	58.1%	63.3%	90.0%	100.0%	100.0%	88.6%	78.3%
**BLOOD TRANSFUSION SERVICES (selected tracer items)**													
Facility offers transfusion services	0 (0.0%)	6 (75.0%)	1 (33.3%)	2 (66.7%)	8 (61.5%)	4 (10.3%)	21 (14.0%)	45 (44.6%)	10 (100.0%)	5 (100.0%)	10 (100.0%)	36 (60.0%)	106 (57.0%)
Blood storage refrigerator	NA	100.0%	100.0%	100.0%	100.0%	75.0%	95.2%	97.8%	100.0%	100.0%	100.0%	94.4%	97.2%
Blood supply sufficiency	NA	33.3%	0.0%	100.0%	75.0%	50.0%	57.1%	35.6%	60.0%	100.0%	60.0%	52.8%	49.1%
Blood supply safety	NA	100.0%	100.0%	100.0%	100.0%	75.0%	95.2%	100.0%	100.0%	100.0%	100.0%	97.2%	99.1%

* Definitions for safe water sources promoted by UNICEF: Piped, public tap, standpipe, tubewell/borehole, protected dug well, protected spring, rain water

** Known as the domain score in WHO SARA methodology

IMEESC Integrated management for emergency and surgical care. NA: Not available. Other = NGO/faith-based/service/research

Note: Adapted General Readiness - disposal of sharps and infectious waste combined, and essential medicines and most diagnostic capacity domains not included as focus of survey was on fracture care. Mean domain scores not calculated for surgical or blood transfusion readiness due to the limited number of variables included.

Supplementary Table 3: Fracture Service Readiness - All facilities

	The Gambia							Zimbabwe					
**Fracture service-specific indicators**	**Health centre/clinic**	**Rural/District Hospital**	**Regional/Provincial Hospital**	**Central Hospital**	**Private**	**Other**	**Total**	**Rural/District Hospital**	**Regional/Provincial Hospital**	**Central Hospital**	**Private**	**Faith-based**	**Total**
**Beds** - Median (range)													
Beds per facility	7.5 (0-28)	52 (0-132)	6 (0-200)	100 (8-456)	13 (0-40)	6 (0-42)	8 (0-456)	35 (0-183)	167.5 (50-394)	500 (140-1200)	59 (20-146)	59 (4-350)	50 (0-1200)
T&O beds per facility	0 (0-27)	0 (0-30)	1 (0-17)	0 (0-28)	0 (0-12)	0 (0-14)	0 (0-30)	0 (0-52)	17 (0-50)	60 (40-92)	7.5 (0-27)	0 (0-30)	0 (0-92)
**Staff***													
Total doctors per facility - Median (range)	0 (0-2.7)	3.1 (0.9-8.9)	0 (0-13.4)	9.8 (4.5-196.1)	4.5 (0-16.9)	0 (0-16.9)	0 (0-196.1)	0.9 (0-15.6)	15.6 (5.5-34.9)	188 (78.9-285.3)	1.8 (0-315.5)	1.8 (0-79.8)	1.8 (0-315.5)
Facilities with doctors (%)	10.7%	100.0%	33.3%	100.0%	92.3%	46.2%	34.0%	56.4%	100.0%	100.0%	60.0%	78.3%	67.2%
General or T&O Surgeons per facility - Median (range)	0 (0-0)	0 (0-1.8)	1.8 (1.8-1.8)	0.9 (0-7)	1.3 (0-2.6)	0 (0-1.8)	0 (0-7)	0 (0-1.8)	1.2 (0-2.5)	3.7 (2.5-4.9)	3.7 (0-43.1)	0 (0-10.5)	0 (0-43.1)
Facilities with general or T&O surgeons (%)	0.0%	37.5%	33.3%	66.7%	69.2%	15.4%	14.0%	5.9%	70.0%	100.0%	40.0%	5.0%	13.4%
General or T&O Nurses per facility - Median (range)	2.9 (0-87.5)	19.7 (8.8-118.9)	16.7 (0-60)	108.1 (8.8-257.5)	4.9 (0-27.5)	2.9 (0-24.6)	2.9 (0-257.5)	22 (0-144.9)	191.8 (50-321.7)	1090 (520.5-1137.9)	44 (1-147.9)	17.5 (2-90.9)	27 (0-1137.9)
Facilities with general or T&O nurses (%)	97.6%	100.0%	66.7%	100.0%	92.3%	97.4%	96.7%	99.0%	100.0%	100.0%	100.0%	100.0%	99.5%
Physiotherapists per facility - Median (range)	0 (0-2.7)	3.1 (0.9-8.9)	0 (0-13.4)	9.8 (4.5-196.1)	4.5 (0-16.9)	0 (0-16.9)	0 (0-196.1)	0 (0-5)	2 (0-7)	8 (1-18)	1 (0-6)	0 (0-9)	0 (0-18)
Facilities with physiotherapists (%)	0.0%	25.0%	33.3%	33.3%	30.8%	5.1%	6.7%	19.8%	80.0%	100.0%	60.0%	20.0%	27.4%
**Imaging**** - N (%)													
Digital/non-digital radiography	0 (0.0%)	1 (12.5%)	1 (33.3%)	1 (33.3%)	5 (38.5%)	2 (5.1%)	10 (6.7%)	18 (17.8%)	9 (90.0%)	5 (100.0%)	6 (60.0%)	18 (30.0%)	56 (30.1%)
MRI (magnetic resonance imaging)	0 (0.0%)	0 (0.0%)	0 (0.0%)	1 (33.3%)	0 (0.0%)	0 (0.0%)	1 (0.7%)	0 (0.0%)	0 (0.0%)	0 (0.0%)	1 (10.0%)	1 (1.7%)	2 (1.1%)
CT scan (computer tomography)	0 (0.0%)	0 (0.0%)	0 (0.0%)	1 (33.3%)	0 (0.0%)	0 (0.0%)	1 (0.7%)	0 (0.0%)	2 (20.0%)	2 (40.0%)	4 (40.0%)	1 (1.7%)	9 (4.8%)
**Fracture Treatment** - N (%)													
Splints (for limb fractures)	17 (20.2%)	3 (37.5%)	1 (33.3%)	1 (33.3%)	8 (61.5%)	8 (20.5%)	38 (25.3%)	55 (54.5%)	10 (100.0%)	4 (80.0%)	9 (90.0%)	39 (65.0%)	117 (62.9%)
Slings (for arm fractures)	30 (35.7%)	2 (25.0%)	1 (33.3%)	0 (0.0%)	7 (53.8%)	11 (28.2%)	51 (34.0%)	60 (59.4%)	8 (80.0%)	5 (100.0%)	10 (100.0%)	44 (73.3%)	127 (68.3%)
Plaster of Paris	9 (10.7%)	4 (50.0%)	1 (33.3%)	1 (33.3%)	8 (61.5%)	6 (15.4%)	29 (19.3%)	45 (44.6%)	9 (90.0%)	5 (100.0%)	10 (100.0%)	43 (71.7%)	112 (60.2%)
Lower limb traction	1 (1.2%)	3 (37.5%)	1 (33.3%)	2 (66.7%)	3 (23.1%)	2 (5.1%)	12 (8.0%)	34 (33.7%)	9 (90.0%)	5 (100.0%)	9 (90.0%)	28 (46.7%)	85 (45.7%)
Walking aids (e.g. crutches)	15 (17.9%)	4 (50.0%)	2 (66.7%)	1 (33.3%)	8 (61.5%)	20 (51.3%)	50 (33.3%)	47 (46.5%)	8 (80.0%)	5 (100.0%)	10 (100.0%)	42 (70.0%)	112 (60.2%)
None of those available	33 (39.3%)	1 (12.5%)	1 (33.3%)	1 (33.3%)	2 (15.4%)	13 (33.3%)	51 (34.0%)	23 (22.8%)	0 (0.0%)	0 (0.0%)	0 (0.0%)	7 (11.7%)	30 (16.1%)
**Medicines***** - N (%)													
Paracetamol	83 (98.8%)	8 (100.0%)	3 (100.0%)	3 (100.0%)	13 (100.0%)	38 (97.4%)	148 (98.7%)	72 (71.3%)	8 (80.0%)	5 (100.0%)	10 (100.0%)	55 (91.7%)	150 (80.6%)

Codeine	4 (4.8%)	1 (12.5%)	0 (0.0%)	1 (33.3%)	6 (46.2%)	10 (25.6%)	22 (14.7%)	10 (9.9%)	2 (20.0%)	1 (20.0%)	9 (90.0%)	9 (15.0%)	31 (16.7%)
Morphine	4 (4.8%)	4 (50.0%)	1 (33.3%)	2 (66.7%)	5 (38.5%)	8 (20.5%)	24 (16.0%)	26 (25.7%)	3 (30.0%)	2 (40.0%)	10 (100.0%)	13 (21.7%)	54 (29.0%)
Alendronic Acid	3 (3.6%)	1 (12.5%)	1 (33.3%)	0 (0.0%)	1 (7.7%)	1 (2.6%)	7 (4.7%)	3 (3.0%)	0 (0.0%)	0 (0.0%)	5 (50.0%)	1 (1.7%)	9 (4.8%)
Zoledronate	1 (1.2%)	1 (12.5%)	0 (0.0%)	0 (0.0%)	0 (0.0%)	2 (5.1%)	4 (2.7%)	2 (2.0%)	0 (0.0%)	0 (0.0%)	4 (40.0%)	0 (0.0%)	6 (3.2%)
Calcium supplement tablets	13 (15.5%)	3 (37.5%)	1 (33.3%)	2 (66.7%)	8 (61.5%)	10 (25.6%)	37 (24.7%)	10 (9.9%)	1 (10.0%)	1 (20.0%)	8 (80.0%)	8 (13.3%)	28 (15.1%)
Vitamin D supplements	14 (16.7%)	2 (25.0%)	1 (33.3%)	1 (33.3%)	8 (61.5%)	13 (33.3%)	39 (26.0%)	12 (11.9%)	1 (10.0%)	1 (20.0%)	8 (80.0%)	8 (13.3%)	30 (16.1%)
Normal saline IV solution	76 (90.5%)	8 (100.0%)	3 (100.0%)	3 (100.0%)	12 (92.3%)	34 (87.2%)	136 (90.7%)	94 (93.1%)	9 (90.0%)	4 (80.0%)	10 (100.0%)	57 (95.0%)	174 (93.5%)
Ringers lactate/Hartmann's IV solution	49 (58.3%)	7 (87.5%)	3 (100.0%)	2 (66.7%)	10 (76.9%)	29 (74.4%)	100 (66.7%)	96 (95.0%)	9 (90.0%)	4 (80.0%)	10 (100.0%)	59 (98.3%)	178 (95.7%)
% dextrose IV solution	64 (76.2%)	6 (75.0%)	3 (100.0%)	3 (100.0%)	11 (84.6%)	29 (74.4%)	116 (77.3%)	78 (77.2%)	7 (70.0%)	5 (100.0%)	10 (100.0%)	46 (76.7%)	146 (78.5%)
Skin disinfectant	71 (84.5%)	8 (100.0%)	3 (100.0%)	3 (100.0%)	12 (92.3%)	30 (76.9%)	127 (84.7%)	96 (95.0%)	10 (100.0%)	5 (100.0%)	9 (90.0%)	55 (91.7%)	175 (94.1%)
Gowns	66 (78.6%)	7 (87.5%)	3 (100.0%)	2 (66.7%)	12 (92.3%)	29 (74.4%)	119 (79.3%)	100 (99.0%)	10 (100.0%)	5 (100.0%)	10 (100.0%)	60 (100.0%)	185 (99.5%)
Eye protection (goggles or face shields)	41 (48.8%)	6 (75.0%)	2 (66.7%)	1 (33.3%)	10 (76.9%)	19 (48.7%)	79 (52.7%)	99 (98.0%)	9 (90.0%)	5 (100.0%)	10 (100.0%)	58 (96.7%)	181 (97.3%)
Medical (surgical or procedural) masks	66 (78.6%)	7 (87.5%)	3 (100.0%)	3 (100.0%)	10 (76.9%)	27 (69.2%)	116 (77.3%)	98 (97.0%)	9 (90.0%)	5 (100.0%)	10 (100.0%)	60 (100.0%)	182 (97.8%)
Absorbable suture material	69 (82.1%)	7 (87.5%)	3 (100.0%)	2 (66.7%)	11 (84.6%)	29 (74.4%)	121 (80.7%)	89 (88.1%)	8 (80.0%)	5 (100.0%)	10 (100.0%)	54 (90.0%)	166 (89.2%)
Non-absorbable suture material	73 (86.9%)	7 (87.5%)	3 (100.0%)	2 (66.7%)	12 (92.3%)	28 (71.8%)	125 (83.3%)	82 (81.2%)	8 (80.0%)	5 (100.0%)	10 (100.0%)	52 (86.7%)	157 (84.4%)

*Adjusted for part time workers. **Available and functional and there is a staff member trained/qualified to use it. *** At least one available and in date. Other = NGO/faith-based/mission/service/research

Supplementary Table 4: Fracture Service Readiness by Rural/Urban catchment

	The Gambia				Zimbabwe			
	Rural	Urban	Mixed	Total	Rural	Urban	Mixed	Total
**N facilities**	100	49	1	150	117	28	41	186
**Fracture service-specific indicators**								
**Number of inpatient beds** - Median (range)								
Adult, non-maternity beds per facility	8.5 (0-200)	8 (0-456)	19 (19-19)	8 (0-456)	40 (0-240)	100 (0-394)	80 (0-1200)	50 (0-1200)
T&O beds per facility	0 (0-30)	0 (0-27)	0 (0-0)	0 (0-30)	0 (0-28)	5.5 (0-50)	0 (0-92)	0 (0-92)
**Staff***								
Total doctors per facility - Median (range)	0 (0-13.4)	1.8 (0-196.1)	4.5 (4.5-4.5)	0 (0-196.1)	0.9 (0-5.5)	3.7 (0-315.5)	3.7 (0-285.3)	1.8 (0-315.5)
Facilities with doctors (%)	17.0%	67.3%	100.0%	34.0%	54.7%	92.9%	85.4%	67.2%
General or T&O Surgeons per facility - Median (range)	0 (0-1.8)	0 (0-7)	0.9 (0.9-0.9)	0 (0-7)	0 (0-1.2)	0 (0-43.1)	0 (0-4.9)	0 (0-43.1)
Facilities with general or T&O surgeons (%)	5.0%	30.6%	100.0%	14.0%	3.4%	42.9%	22.0%	13.4%
General or T&O Nurses per facility - Median (range)	2.9 (0-64.9)	6.9 (0-257.5)	4.9 (4.9-4.9)	2.9 (0-257.5)	15 (0-99.9)	56.9 (1-321.7)	52 (2-1137.9)	27 (0-1137.9)
Facilities with general or T&O nurses (%)	97.0%	95.9%	100.0%	96.7%	99.1%	100.0%	100.0%	99.5%
Physiotherapists per facility - Median (range)	0.0 (0.0-2.9)	0.0 (0.0-8.8)	1.0 (1.0-1.0)	0.0 (0.0-8.8)	0 (0-5)	1 (0-9)	0 (0-18)	0 (0-18)
Facilities with physiotherapists (%)	4.0%	10.2%	100.0%	6.7%	14.5%	57.1%	43.9%	27.4%
**Imaging**** - N (%)								
Digital/non-digital radiography	3 (3.0%)	7 (14.3%)	0 (0.0%)	10 (6.7%)	23 (19.7%)	15 (53.6%)	18 (43.9%)	56 (30.1%)
MRI (magnetic resonance imaging)	0 (0.0%)	1 (2.0%)	0 (0.0%)	1 (0.7%)	0 (0.0%)	2 (7.1%)	0 (0.0%)	2 (1.1%)
CT scan (computer tomography)	0 (0.0%)	1 (2.0%)	0 (0.0%)	1 (0.7%)	0 (0.0%)	7 (25.0%)	2 (4.9%)	9 (4.8%)
**Fracture Treatment** - N (%)								
Splints (for limb fractures)	24 (24.0%)	13 (26.5%)	1 (100.0%)	38 (25.3%)	71 (60.7%)	20 (71.4%)	26 (63.4%)	117 (62.9%)
Slings (for arm fractures)	36 (36.0%)	14 (28.6%)	1 (100.0%)	51 (34.0%)	76 (65.0%)	21 (75.0%)	30 (73.2%)	127 (68.3%)
Plaster of Paris	15 (15.0%)	13 (26.5%)	1 (100.0%)	29 (19.3%)	60 (51.3%)	19 (67.9%)	33 (80.5%)	112 (60.2%)
Lower limb traction	4 (4.0%)	8 (16.3%)	0 (0.0%)	12 (8.0%)	1 (0.9%)	0 (0.0%)	1 (1.7%)	2 (1.1%)
Walking aids (e.g. crutches)	29 (29.0%)	20 (40.8%)	1 (100.0%)	50 (33.3%)	61 (52.1%)	21 (75.0%)	30 (73.2%)	112 (60.2%)
None of those available	31 (31.0%)	20 (40.8%)	0 (0.0%)	51 (34.0%)	24 (20.5%)	1 (3.6%)	5 (12.2%)	30 (16.1%)
**Medicines***** - N (%)								
Paracetamol	99 (99.0%)	48 (98.0%)	1 (100.0%)	148 (98.7%)	92 (78.6%)	26 (92.9%)	32 (78.0%)	150 (80.6%)
Codeine	11 (11.0%)	10 (20.4%)	1 (100.0%)	22 (14.7%)	12 (10.3%)	10 (35.7%)	9 (22.0%)	31 (16.7%)
Morphine	11 (11.0%)	12 (24.5%)	1 (100.0%)	24 (16.0%)	23 (19.7%)	15 (53.6%)	16 (39.0%)	54 (29.0%)
Oral alendronate	4 (4.0%)	3 (6.1%)	0 (0.0%)	7 (4.7%)	3 (2.6%)	3 (10.7%)	3 (7.3%)	9 (4.8%)
IV zoledronate	1 (1.0%)	3 (6.1%)	0 (0.0%)	4 (2.7%)	2 (1.7%)	3 (10.7%)	1 (2.4%)	6 (3.2%)
Calcium supplement tablets	21 (21.0%)	15 (30.6%)	1 (100.0%)	37 (24.7%)	11 (9.4%)	10 (35.7%)	7 (17.1%)	28 (15.1%)

Vitamin D supplements	19 (19.0%)	19 (38.8%)	1 (100.0%)	39 (26.0%)	13 (11.1%)	10 (35.7%)	7 (17.1%)	30 (16.1%)
Normal saline IV solution	91 (91.0%)	44 (89.8%)	1 (100.0%)	136 (90.7%)	111 (94.9%)	26 (92.9%)	37 (90.2%)	174 (93.5%)
Ringers lactate/Hartmann's IV solution	63 (63.0%)	36 (73.5%)	1 (100.0%)	100 (66.7%)	112 (95.7%)	27 (96.4%)	39 (95.1%)	178 (95.7%)
% dextrose IV solution	76 (76.0%)	39 (79.6%)	1 (100.0%)	116 (77.3%)	90 (76.9%)	23 (82.1%)	33 (80.5%)	146 (78.5%)
Skin disinfectant	85 (85.0%)	41 (83.7%)	1 (100.0%)	127 (84.7%)	111 (94.9%)	24 (85.7%)	40 (97.6%)	175 (94.1%)
Gowns	79 (79.0%)	39 (79.6%)	1 (100.0%)	119 (79.3%)	116 (99.1%)	28 (100.0%)	41 (100.0%)	185 (99.5%)
Eye protection (goggles or face shields)	52 (52.0%)	26 (53.1%)	1 (100.0%)	79 (52.7%)	113 (96.6%)	28 (100.0%)	40 (97.6%)	181 (97.3%)
Medical (surgical or procedural) masks	84 (84.0%)	31 (63.3%)	1 (100.0%)	116 (77.3%)	115 (98.3%)	27 (96.4%)	40 (97.6%)	182 (97.8%)
Absorbable suture material	85 (85.0%)	35 (71.4%)	1 (100.0%)	121 (80.7%)	101 (86.3%)	28 (100.0%)	37 (90.2%)	166 (89.2%)
Non-absorbable suture material	87 (87.0%)	37 (75.5%)	1 (100.0%)	125 (83.3%)	94 (80.3%)	28 (100.0%)	35 (85.4%)	157 (84.4%)

*Adjusted for part time workers. **Available and functional and there is a staff member trained/qualified to use it. *** At least one available and in date
Rural = rural and semi-rural catchment only, Urban = urban and semi-urban catchment only, Mixed = urban and rural catchment
IV=intravenous. T&O= Trauma and orthopaedic

Supplementary Table 5: Hip Fracture-specific Readiness - additional items

	The Gambia	Zimbabwe
N Facilities that can diagnose or treat a hip fracture	10	107
Facility has clinical guidelines on osteoporosis management	3 (30.0%)	26 (24.3%)
Facility has clinical guideline on the following for management of a hip fracture:		
Pain management	9 (90.0%)	42 (39.3%)
Diagnostic investigations	8 (80.0%)	40 (37.4%)
Referral for care	7 (70.0%)	50 (46.7%)
None of the above	1 (10.0%)	54 (50.5%)
Days per week facility if open to receive people with suspected hip fractures - median (range)	7 (7-7)	7 (5-7)
Hours per day facility open to receive people with suspected hip fractures? - median (range)	24 (24-24)	24 (7-24)
Staff members present in the facility or on call over 24 hours (including weekends and on public holidays)		
General surgeon	8 (80.0%)	41 (38.3%)
Orthopaedic/ trauma surgeon	6 (60.0%)	21 (19.6%)
Orthopaedic trained nurse	4 (40.0%)	12 (11.2%)
Anaesthetist	9 (90.0%)	58 (54.2%)
Operating theatre assistants	7 (70.0%)	64 (59.8%)
Radiologist	8 (80.0%)	16 (15.0%)
Radiographers	5 (50.0%)	52 (48.6%)
Laboratory technicians	10 (100.0%)	78 (72.9%)
None of the above	0 (0.0%)	11 (10.3%)
No. of HF presentations (last 4 weeks)	2.2, 1 (0-10)	1.7, 0 (0-20)
N low impact	56.7, 75 (0-100)	27, 0 (0-100)
Total HFs	22	183
What percentage of all the people with hip fractures had been referred to your hospital from another health facility?	15.7, 0.5 (0-80)	39.6, 40 (0-100)
Over the last 4 weeks, what percentage of all the people with hip fractures had been referred to your hospital from another health facility?	12.9, 0 (0-90)	13, 0 (0-100)
First contact following hip fracture		
Family	9 (90.0%)	93 (86.9%)
Friends/neighbours	8 (80.0%)	46 (43.0%)
Hospital	4 (40.0%)	64 (59.8%)
Health Centre/Clinic	2 (20.0%)	80 (74.8%)
Community Health worker/nurse	2 (20.0%)	26 (24.3%)
Pharmacy/chemist/dispensary	1 (10.0%)	0 (0.0%)
Community based organisation (CBO) or non-governmental organisation (NGO)	0 (0.0%)	1 (0.9%)
Traditional healers/bonesetters	6 (60.0%)	6 (5.6%)
Religious leader/church	0 (0.0%)	8 (7.5%)
Private doctor/clinic	0 (0.0%)	12 (11.2%)
Other (specify)	0 (0.0%)	1 (0.9%)
If other:		Any person who is nearby (1)
Transport to facility		
Ambulance	0 (0.0)	33 (30.8)
Private transport	5 (50.0)	57 (53.3)
Public transport	5 (50.0)	11 (10.3)
Other (specify)	0 (0.0)	5 (4.7)
Do not know	0 (0.0)	1 (0.9)
If other:		Scotchcart (3), hired car or scotchcart (1), Animal drawn vehicle (1)
Average time to reach the facility (hours) - median (range)	24 (1-720)	6 (0-168)
Has a person with a suspected hip fracture usually received pain medication before they arrive at your facility?		
No	2 (20.0%)	19 (17.8%)
Simple painkillers (e.g. paracetamol)	6 (60.0%)	95 (88.8%)
Oral opiates	0 (0.0%)	8 (7.5%)
Injected opiates	0 (0.0%)	9 (8.4%)
Other (specify below)	0 (0.0%)	0 (0.0%)
Do not know	2 (20.0%)	0 (0.0%)
If other:		
Who does HF assessment		

Nurse	8 (80.0%)	95 (88.8%)
Clinical Officer	2 (20.0%)	28 (26.2%)
General doctor	8 (80.0%)	82 (76.6%)
Emergency department doctor	4 (40.0%)	18 (16.8%)
General surgical doctor	4 (40.0%)	2 (1.9%)
Orthopaedic doctor	5 (50.0%)	16 (15.0%)
Geriatric medicine doctor	NA	NA
Internal medicine doctor	2 (20.0%)	1 (0.9%)
Anaesthetist	0 (0.0%)	8 (7.5%)
Other (specify	0 (0.0%)	7 (6.5%)
If other:		physician (1), Rehab technician (5), physiotherapists and rehab technician (1)
Pain relief offered		
Simple painkillers such as paracetamol	8 (80.0%)	85 (79.4%)
Oral opiates	2 (20.0%)	38 (35.5%)
Intramuscular injection of opiates	8 (80.0%)	74 (69.2%)
Intravenous injection of opiates	4 (40.0%)	17 (15.9%)
Nerve block (e.g. of the femoral nerve)	0 (0.0%)	3 (2.8%)
Average wait time in hours from presentation to admission - median (range)	1 (0-48)	1 (0-12)
Average waiting time in hours from arrival at your facility to onward referral for hip fracture care - median (range)	0.5 (0-6)	2 (0-24)

Supplementary Table 6: Hip fracture surgical readiness

	The Gambia	Zimbabwe Public	Zimbabwe Private*
**N facilities that can perform hip fracture surgery**	**n=4**	**n=9**	**n=8**
Who routinely assesses the patient on the ward before surgery for a hip fracture - N(%)			
Clinical Officer	2 (50.0%)	0 (0.0%)	0 (0.0%)
General surgeon	2 (50.0%)	2 (22.2%)	5 (62.5%)
Orthopaedic surgeon	4 (100.0%)	9 (100.0%)	8 (100.0%)
Geriatric medicine doctor	0 (0.0%)	0 (0.0%)	0 (0.0%)
Internal medicine doctor	2 (50.0%)	0 (0.0%)	3 (37.5%)
Anaesthetist	1 (25.0%)	6 (66.7%)	7 (87.5%)
Other (specify)	0 (0.0%)	0 (0.0%)	1 (12.5%)
If other:			*Physiotherapist (1)*
Cognition routinely assessed (Y - 4AT) - N(%)			
10 question Abbreviated Mini-mental Test (AMT)	0 (0.0%)	3 (33.3%)	3 (37.5%)
4AT	2 (50.0%)	0 (0.0%)	0 (0.0%)
No	2 (50.0%)	5 (55.6%)	5 (62.5%)
Other	0 (0.0%)	1 (11.1%)	0 (0.0%)
If other:		*they do assessments without a checklist (1)*	
Average time from presentation to surgery (days) - median (range)	8 (2-48)	14 (0-60)	2 (1-3)
Percentage of patients with hip fracture that receive surgery - median (range)	58% (25-70%)	75% (4-100%)	99% (90-100%)
Importance of various factors on deciding to operate (1 to 10 with 1 being "not at all important" and 10 being "extremely important") - median (range)			
Patient's health?	9.0 (7.0-10.0)	10.0 (8.0-10.0)	10.0 (10.0-10.0)
Patient's age?	4.5 (2.0-10.0)	8.0 (2.0-10.0)	10.0 (5.0-10.0)
Patient's wishes?	9.0 (5.0-10.0)	10.0 (5.0-10.0)	10.0 (10.0-10.0)
Family's wishes?	8.5 (3.0-10.0)	7.0 (2.0-8.0)	9.0 (8.0-10.0)
Patient's ability to pay for the operation?	8.0 (5.0-10.0)	5.0 (1.0-10.0)	10.0 (10.0-10.0)
Availability of equipment?	9.5 (5.0-10.0)	10.0 (8.0-10.0)	10.0 (10.0-10.0)
Availability of surgical expertise?	10.0 (8.0-10.0)	10.0 (10.0-10.0)	10.0 (10.0-10.0)
Availability of anaesthetic expertise?	10.0 (8.0-10.0)	10.0 (10.0-10.0)	10.0 (10.0-10.0)
Are there any other factors, if so please specify these factor/s:	*Patients willingness to sign the consent form (1), Alternative treatment (1)*	*where they stay and ability to come for reviews (1)*	*Premorbid function of the patient (1)*
Important of other factors	1.0 (1.0-10.0)	7.0, 7.0 (7.0-7.0)	7.0, 7.0 (7.0-7.0)
Anaesthesia, percentage of hip fracture patients - median (range):			
having surgery under general anaesthesia	3.0% (0-75%)	30.0% (10-100%)	32.5% (5-90%)
having surgery under spinal anaesthesia	72.5% (10-99%)	70% (0-90%)	67.5% (10-95%)
that get a regional nerve block (e.g. of the femoral nerve) as part of their anaesthetic	0% (0-30%)	0% (0-90%)	5% (0-90%)
Anaesthesia for hip fracture surgery usually given by - N(%):			
A fully qualified anaesthetist	2 (50.0%)	7 (77.8%)	8 (100.0%)
An anaesthetist in training	0 (0.0%)	1 (11.1%)	0 (0.0%)
An anaesthetic assistant			
Other (specify)	2 (50.0%)	1 (11.1%)	0 (0.0%)
	Nurse Anaesthetist (2)	*Nurse Anaesthetist (1)*	
Hip fracture fixation prostheses available - N(%)			
Total hip replacement (THR)	3 (75.0%)	2 (22.2%)	3 (37.5%)
Hip hemi-arthroplasty	3 (75.0%)	6 (66.7%)	3 (37.5%)
Intramedullary (IM) nail	2 (50.0%)	4 (44.4%)	3 (37.5%)
Cannulated screws	4 (100.0%)	2 (22.2%)	3 (37.5%)
Sliding hip screw	4 (100.0%)	2 (22.2%)	2 (25.0%)
None of those	0 (0.0%)	3 (33.3%)	5 (62.5%)
**Hospital's preferred surgical option for:**			
For patients with an undisplaced intra-capsular fracture - N(%)			
Screw fixation	1 (25.0%)	2 (22.2%)	2 (25.0%)
Sliding hip screw fixation	0 (0.0%)	4 (44.4%)	2 (25.0%)
Hemi-arthroplasty cemented	3 (75.0%)	0 (0.0%)	0 (0.0%)
Hemi-arthroplasty uncemented	0 (0.0%)	2 (22.2%)	1 (12.5%)
Total hip replacement (THR)	0 (0.0%)	0 (0.0%)	1 (12.5%)
No standardisation	0 (0.0%)	1 (11.1%)	1 (12.5%)
Other (specify)	0 (0.0%)	0 (0.0%)	0 (0.0%)
Do not know	0 (0.0%)	0 (0.0%)	1 (12.5%)

If No standardisation:	NA	*Varies with patient's age, condition and radiography results (1)*	*The choice depends on premorbid function, cognitive function and ability to withstand the operation (1)*
If Other:	Not stated	NA	NA
For patients with a displaced intra-capsular fracture - N(%)			
Screw fixation	1 (25.0%)	0 (0.0%)	0 (0.0%)
Sliding hip screw fixation	0 (0%)	0 (0%)	0 (0%)
Hemi-arthroplasty cemented	3 (75.0%)	1 (11.1%)	1 (12.5%)
Hemi-arthroplasty uncemented	0 (0.0%)	0 (0.0%)	1 (12.5%)
Total hip replacement (THR)	0 (0.0%)	5 (55.6%)	3 (37.5%)
No standardisation	0 (0.0%)	2 (22.2%)	2 (25.0%)
Other (specify)	0 (0.0%)	1 (11.1%)	0 (0.0%)
Do not know	0 (0.0%)	0 (0.0%)	1 (12.5%)
If No standardisation:	NA	*Depends on patients age. The young ones gets fixation and the old ones replacement surgery (1)*<br>*varies from patient to patient depending on patient's age, condition and radiography results (1)*	*The choice of procedure depends with the premorbid status of patient, cognitive function and patient's ability to withstand the operation (1)*<br>*This varies between patients due to other factors like age and ability to withstand the operation (1)*
If Other:	NA	*depends on patient's age. young patients hip screw, old patients arthroplasty (1)*	NA
Patients with good mobility before hip fracture routinely offered a total hip replacement - N(%)			
Yes, cemented	2 (50.0%)	0 (0.0%)	1 (12.5%)
Yes, uncemented	0 (0.0%)	3 (33.3%)	1 (12.5%)
Yes, either cemented or uncemented (i.e. according to surgeon's preference)	0 (0.0%)	3 (33.3%)	6 (75.0%)
No	2 (50.0%)	3 (33.3%)	0 (0.0%)
Do not know	0 ()	0 ()	0 ()
For patients with a two-part extra-capsular fracture - N(%)			
IM (intramedullary) nail fixation	0 (0.0%)	1 (11.1%)	0 (0.0%)
Sliding hip screw fixation	3 (75.0%)	2 (22.2%)	4 (50.0%)
Either IM nail fixation or sliding hip screw fixation (i.e. according to surgeon's preference)	1 (25.0%)	4 (44.4%)	4 (50.0%)
Other (specify)	0 (0.0%)	1 (11.1%)	0 (0.0%)
Do not know	0 (0.0%)	1 (11.1%)	0 (0.0%)
If Other:	NA	*patients are referred to a central hospital (1)*	NA
For patients with a complex (more than two-part) extra-capsular fracture - N(%)			
IM (intramedullary) nail fixation	0 (0.0%)	4 (44.4%)	0 (0.0%)
Sliding hip screw fixation	3 (75.0%)	0 (0.0%)	3 (37.5%)
Either IM nail fixation or sliding hip screw fixation (i.e. according to surgeon's preference)	1 (25.0%)	3 (33.3%)	5 (62.5%)
Other (specify)	0 (0.0%)	1 (11.1%)	0 (0.0%)
Do not know	0 (0.0%)	1 (11.1%)	0 (0.0%)
If Other:	NA	*patients are referred to a central hospital (1)*	NA
For patients with a reverse oblique (lateral wall) extra-capsular fracture - N(%)			
IM (intramedullary) nail fixation	1 (25.0%)	5 (55.6%)	3 (37.5%)
Sliding hip screw fixation	2 (50.0%)	0 (0.0%)	1 (12.5%)
Either IM nail fixation or sliding hip screw fixation (i.e. according to surgeon's preference)	1 (25.0%)	3 (33.3%)	4 (50.0%)
Do not know	0 (0.0%)	1 (11.1%)	0 (0.0%)
Wards patients go to after hip fracture surgery - N(%)			
General ward	0 (0.0%)	2 (22.2%)	2 (25.0%)
Surgical ward	0 (0.0%)	4 (44.4%)	2 (25.0%)
Geriatric ward	0 (0.0%)	0 (0.0%)	0 (0.0%)
Specialist orthopaedic ward	1 (25.0%)	4 (44.4%)	1 (12.5%)
Other (specify below)	3 (75.0%)	1 (11.1%)	5 (62.5%)
If Other:	*ICU (2), Private rooms (1)*	*HDU and ICU (1)*	*HDU or ICU (5)*
Doctors who are routinely involved in the care of patients with hip fractures whilst in the facility - N(%):			

Anaesthetists	3 (75.0%)	5 (55.6%)	8 (100.0%)
Orthopaedic surgeons	4 (100.0%)	9 (100.0%)	8 (100.0%)
Geriatricians	0 (0.0%)	1 (11.1%)	1 (12.5%)
General internal medics	3 (75.0%)	1 (11.1%)	3 (37.5%)
Palliative care doctors	1 (25.0%)	0 (0.0%)	0 (0.0%)
Rehabilitation doctors	1 (25.0%)	2 (22.2%)	1 (12.5%)
Other (specify below)	1 (25.0%)	0 (0.0%)	4 (50.0%)
If Other:	*General surgeon (1)*	*NA*	*Physicians (2)*
Staff routinely involved in the care of patients with hip fractures whilst in the facility - N(%)			
General nurses	4 (100.0%)	9 (100.0%)	8 (100.0%)
Specialist orthopaedic nurses	3 (75.0%)	7 (77.8%)	3 (37.5%)
Physiotherapists	3 (75.0%)	9 (100.0%)	8 (100.0%)
Occupational therapists	1 (25.0%)	5 (55.6%)	2 (25.0%)
Dietitians	0 (0.0%)	6 (66.7%)	4 (50.0%)
Pharmacists	1 (25.0%)	7 (77.8%)	8 (100.0%)
Social workers	0 (0.0%)	7 (77.8%)	2 (25.0%)
Case managers	0 (0.0%)	2 (22.2%)	1 (12.5%)
Percentage of patients with hip fractures develop the following complications - median (range)			
Pressure sores	2.5% (0-5%)	10% (0-40%)	1% (0-5%)
DVT or PE	8% (2-48%)	14% (0-60%)	2% (1-3%)
Post-operative wound infection	1.5% (0-5%)	5% (0-15%)	1% (0-5%)
Other post-operative wound infections, including pneumonia and urinary infection	3.5% (0-5%)	4.8%, 2% (0-20%)	1.5% (0-5%)
Percentage of patients routinely allowed to put their full weight on their injured leg in the first 24 hours after surgery - mean (range)	43.8% (0-95%)	33.7% (0-90%)	33.0% (0-99%)
N days after surgery patients allowed to put their full weight on their operated leg after - mean (range):			
Arthroplasty	1.0 (0-2)	21.9 (2-99)	11.3 (1-70)
An internal fixation of an intracapsular hip fracture	5.8 (0-21)	30.9 (3-99)	11.4 (1-70)
An internal fixation of an extracapsular hip fracture	1.2 (0-2)	31.7 (3-99)	11.4 (1-70)
If you would like to provide more information on weight bearing post-surgery, please do so here	NA	NA	*Surgeon indicated that there are unique patient-specific factors they consider before putting patients on full weight. Generally it will be painful to put patients on full weight immediately post-surgery so we do it gradually (1)*
Days after surgery routinely start mobilising patients - mean (range)	1.5 (1-3)	6.0 (1-30)	1.1 (1-2)
Who helps the patients to mobilise - N(%)			
General Nurse	2 (50.0%)	3 (33.3%)	5 (62.5%)
Orthopaedic nurse	2 (50.0%)	3 (33.3%)	0 (0.0%)
Physiotherapist	3 (75.0%)	9 (100.0%)	8 (100.0%)
Occupational therapist	1 (25.0%)	1 (11.1%)	1 (12.5%)
Family	0 (0.0%)	2 (22.2%)	0 (0.0%)
Equipment routinely have available to help mobilise patients - N(%)			
Walking sticks	2 (50.0%)	4 (44.4%)	3 (37.5%)
Crutches	3 (75.0%)	7 (77.8%)	7 (87.5%)
Walking (Zimmer) frame	4 (100.0%)	8 (88.9%)	8 (100.0%)
Pulpi frame	0 (0.0%)	0 (0.0%)	1 (12.5%)
Sara StedyTM	0 (0.0%)	0 (0.0%)	1 (12.5%)
Wheelchair	3 (75.0%)	8 (88.9%)	6 (75.0%)
Hoist	0 (0.0%)	0 (0.0%)	1 (12.5%)
What equipment are you able to provide for patients at discharge to take home - N(%): Walking sticks			
Available at no cost to patient	1 (25.0%)	0 (0.0%)	0 (0.0%)
Available to patient if they pay	0 (0.0%)	1 (11.1%)	3 (37.5%)
Not available	3 (75.0%)	8 (88.9%)	5 (62.5%)
Crutches			
Available at no cost to patient	2 (50.0%)	0 (0.0%)	0 (0.0%)
Available to patient if they pay	2 (50.0%)	5 (55.6%)	3 (37.5%)
Not available	0 (0.0%)	4 (44.4%)	5 (62.5%)
Walking (Zimmer) frame			
Available at no cost to patient	3 (75.0%)	0 (0.0%)	0 (0.0%)

Available to patient if they pay	1 (25.0%)	5 (55.6%)	3 (37.5%)
Not available	0 (0.0%)	4 (44.4%)	5 (62.5%)
Wheelchair			
Available at no cost to patient	1 (25.0%)	0 (0.0%)	0 (0.0%)
Available to patient if they pay	1 (25.0%)	4 (44.4%)	3 (37.5%)
Not available	2 (50.0%)	5 (55.6%)	5 (62.5%)
Hoist			
Available at no cost to patient	0 (0.0%)	0 (0.0%)	0 (0.0%)
Available to patient if they pay	0 (0.0%)	1 (11.1%)	1 (12.5%)
Not available	4 (100.0%)	8 (88.9%)	7 (87.5%)
Average length of stay in days in those operated - mean (range)	3.2 (2-5)	23.6 (5-60)	5.2 (3-7)
Average length of stay in days of those non-operated - mean (range)	1.2 (0-3)	35.8 (7-90)	17.5 (0-42)
Medication prescribed for osteoporosis before discharge, if indicated - N(%)			
Calcium and vitamin D supplements	3 (75.0%)	8 (88.9%)	6 (75.0%)
Alendronate (oral tablets)	0 (0.0%)	2 (22.2%)	0 (0.0%)
Risedronate (oral tablets)	0 (0.0%)	0 (0.0%)	0 (0.0%)
Ibandronic acid (oral tablets)	0 (0.0%)	0 (0.0%)	0 (0.0%)
Zoledronate (intravenous)	0 (0.0%)	1 (11.1%)	4 (50.0%)
Hormone replacement therapy (for women)	0 (0.0%)	2 (22.2%)	1 (12.5%)
Raloxifine (oral tablets)	0 (0.0%)	0 (0.0%)	0 (0.0%)
Strontium ranelate (oral sachets)	0 (0.0%)	0 (0.0%)	0 (0.0%)
Teriparatide (subcutaneous injections)	0 (0.0%)	0 (0.0%)	0 (0.0%)
Investigations used to identify people who needs medications for osteoporosis			
X-ray	2 (50.0%)	7 (77.8%)	3 (37.5%)
DXA scan (of hip and lumbar spine)	0 (0.0%)	3 (33.3%)	5 (62.5%)
DXA VFA scan (vertebral fracture assessment)	0 (0.0%)	3 (33.3%)	5 (62.5%)
CT-scan	0 (0.0%)	3 (33.3%)	5 (62.5%)
MRI	0 (0.0%)	3 (33.3%)	5 (62.5%)
Blood tests for bone turnover markers	0 (0.0%)	3 (33.3%)	5 (62.5%)
Fracture risk assessment tool (e.g. FRAX)	0 (0.0%)	3 (33.3%)	5 (62.5%)
Other	0 (0.0%)	3 (33.3%)	5 (62.5%)
None	0 (0.0%)	3 (33.3%)	5 (62.5%)
DXA scanner available and functional			
Available and functional	0 (0.0%)	0 (0.0%)	1 (12.5%)
Available but non-functional	0 (0.0%)	0 (0.0%)	0 (0.0%)
Available but functionality unknown	0 (0.0%)	0 (0.0%)	0 (0.0%)
Not available	4 (100.0%)	9 (100.0%)	7 (87.5%)
For hip fracture patients, do you have:			
A written treatment plan for care before hospital (e.g. ambulance care)	0 (0.0%)	2 (22.2%)	0 (0.0%)
Standardised admission paperwork (e.g. hip fracture admission proforma)	1 (25.0%)	3 (33.3%)	1 (12.5%)
Protocol/ policy for pain management before surgery	1 (25.0%)	4 (44.4%)	2 (25.0%)
Protocol/ policy for pain management after surgery	2 (50.0%)	4 (44.4%)	2 (25.0%)
Standardised assessment of readiness to be discharged	0 (0.0%)	2 (22.2%)	1 (12.5%)
Written treatment plan for after discharge	3 (75.0%)	4 (44.4%)	1 (12.5%)
Local guideline for osteoporosis management	0 (0.0%)	2 (22.2%)	1 (12.5%)
None of the above	1 (25.0%)	5 (55.6%)	6 (75.0%)
Data on patients with a hip fracture after they have been discharged routinely collected	1 (25.0%)	3 (33.3%)	4 (50.0%)
What data do you routinely collect after discharge?			
Death	0 (0.0%)	2 (22.2%)	2 (25.0%)
Cause of death	0 (0.0%)	2 (22.2%)	2 (25.0%)
Re-admission for hip fracture/surgery related problem	1 (25.0%)	3 (33.3%)	4 (50.0%)
Re-operation	1 (25.0%)	3 (33.3%)	4 (50.0%)

*Includes one faith-based facility

Supplementary Table 7: Traditional bone-setter availability and readiness (The Gambia)

	**Total**	**Rural/ Semi Rural**	**Urban/ Peri Urban**			
**Catchment**	35	17 (48.6%)	23 (65.7%)			
TBS Training and work pattern						
**Trained to be a TBS by:**	**A bone setter from my family**	**A local bone setter**	**I trained myself**			
	32 (91.4%)	1 (2.9%)	2 (5.7%)			
**Days per week usually work as a bone-setter**	**≥5 days**	**<5 days**				
	24 (68.6%)	11 (31.4%)				
	**Mean**	**Range**				
**Years worked as a bone-setter**	26.1	(2-71)				
**How many people work with the TBS**	1.7	(0-3)				
Fracture treatment		**Frequency of presentation**			**Suggest the patient has an X-ray**	**Able to set**
**Injuries**	**Never**	**Rare**	**Sometimes**	**Common**		
Fingers/hands	0 (0.0%)	1 (2.9%)	5 (14.3%)	29 (82.9%)	12 (34.3%)	34 (97.1%)
Arms (skin intact)	0 (0.0%)	2 (5.7%)	14 (40.0%)	19 (54.3%)	10 (28.6%)	27 (77.1%)
Arms (skin broken)	1 (2.9%)	5 (14.3%)	6 (17.1%)	23 (65.7%)	19 (54.3%)	29 (82.9%)
Hip (top quarter of thigh bone/femur)	7 (20.0%)	7 (20.0%)	11 (31.4%)	10 (28.6%)	24 (68.6%)	18 (51.4%)
Legs (excluding hips, skin intact)	6 (17.1%)	5 (14.3%)	11 (31.4%)	13 (37.1%)	18 (51.4%)	29 (82.9%)
Legs (excluding hips, skin broken)	6 (17.1%)	4 (11.4%)	13 (37.1%)	12 (34.3%)	22 (62.9%)	23 (65.7%)
Toes/feet	0 (0.0%)	1 (2.9%)	9 (25.7%)	25 (71.4%)	7 (20.0%)	34 (97.1%)
Face/nose	21 (60.0%)	12 (34.3%)	1 (2.9%)	1 (2.9%)	0 (0.0%)	2 (5.7%)
Neck	17 (48.6%)	13 (37.1%)	4 (11.4%)	1 (2.9%)	8 (22.9%)	11 (31.4%)
Spine	13 (37.1%)	15 (42.9%)	5 (14.3%)	2 (5.7%)	9 (25.7%)	10 (28.6%)
**Pain relief used when treating patients with fractures**	**Never**	**Sometimes**	**Often**			
Paracetamol	26 (74.3%)	8 (22.9%)	1 (2.9%)			
Ibuprofen/Diclofenac/Naproxen	33 (94.3%)	1 (2.9%)	1 (2.9%)			
Codeine	35 (100.0%)	0 (0.0%)	0 (0.0%)			
Morphine	35 (100.0%)	0 (0.0%)	0 (0.0%)			
Traditional medication and/or herbs	14 (40.0%)	10 (28.6%)	11 (31.4%)			
Other	None					
	None					

Referral for fractures cannot set	To another bone setter	To a clinical facility	No
	13 (37.1%)	18 (51.4%)	10 (28.6%)

Records kept of fractures treated	Number only	Number and type	No
	0 (0.0%)	4 (11.4%)	31 (88.6%)

Management of a hip fracture?	Splinting	Manipulation	Traction	Herbal/traditional medicines	Hospital referral	Not at all
	7 (20.0%)	5 (14.3%)	2 (5.7%)	20 (57.1%)	5 (14.3%)	11 (31.4%)

	Median	Range
Number of fractures in last 4 weeks	5	0-35
Number of fractures seen in last year	25	0-350
Number of hip fractures in last 4 weeks	0	0-4
Number of hip fracture seen in last year	0	0-30
Number of hip fractures confirmed by x-ray	0	0-10
Percent of hip fractures referred for X-ray	0%	0-100%
Percent of hip fractures referred for fracture management	0%	0-100%

Equipment available for the treatment of patients with fractures	Splints	Slings	Plaster of Paris	Traction	Walking sticks	Crutches	Wheelchair
	11 (31.4%)	7 (20.0%)	10 (28.6%)	2 (5.7%)	8 (22.9%)	2 (5.7%)	1 (2.9%)

Infrastructure	Centrally supplied electricity	Light source available	Landline phone available	mobile phone available	Internet (today)	Clean running water available	Functioning flush toilet
	22 (75.9%)	31 (88.6%)	0 (0.0%)	34 (97.1%)	1 (2.9%)	9 (25.7%)	3 (8.6%)

	Mean	Range
Average time to reach the facility (hours)	55.7	0-576

Transport	For visiting patients	Access to in order to help patients reach TBS
Ambulance	NA	0 (0.0%)
Motorbike	10 (28.6%)	13 (37.1%)
Private car	3 (8.6%)	20 (57.1%)
Private 4WD	0 (0.0%)	0 (0.0%)
Taxi car	13 (37.1%)	21 (60.0%)
Public bus	2 (5.7%)	1 (2.9%)
Bicycle	10 (28.6%)	3 (8.6%)
Ox cart	0 (0.0%)	5 (14.3%)
Donkey cart	3 (8.6%)	12 (34.3%)
I don't visit patients	10 (28.6%)	NA
None	NA	8 (22.9%)

Sterilisation and sanitation		
**Final disposal of medical waste (e.g. bandages)**		
Open burning, flat ground, no protection	7 (20.0%)	
Open burning, pit or protected ground	6 (17.1%)	
Dump without burning, flat ground, open pit-no protection	5 (14.3%)	
Dump without burning, ground or pit with protection (includes burying)	5 (14.3%)	
Stored in covered container	3 (8.6%)	
Stored in other protected environment	9 (25.7%)	
Stored unprotected	0 (0.0%)	
	**Available**	**Not available**
**Processing of equipment for reuse**		
Non-electric pot with cover for boiling/steam	7 (20.0%)	28 (80.0%)
Heat source for cleaning non-electric equipment	0 (0.0%)	35 (100.0%)
	**Available**	**Not available**
**Resources/supplies used for infection control**		
Clean running water (piped, bucket with tap, or pour pitcher)	15 (42.9%)	20 (57.1%)
Handwashing soap, liquid soap	13 (34.3%)	23 (65.7%)
Alcohol-based hand rub	0 (0.0%)	35 (100.0%)
Disposable latex gloves	0 (0.0%)	35 (100.0%)
Waste receptacle (pedal bin) with lid and plastic bin liners	0 (0.0%)	35 (100.0%)
Sharps container ("safety box")	0 (0.0%)	35 (100.0%)
Environmental disinfectant (e.g. chlorine, alcohol)	0 (0.0%)	35 (100.0%)

Supplementary Table 8: Interrater Agreement

	Agreement	Expected agreement	Kappa	SE	Z	Prob>Z
**Medical facilities - Zimbabwe and The Gambia (N repeats = 37, 11.0%)**						
Does your facility have electricity?	97.2%	87.0%	0.79	0.163	4.83	<0.001
Patients with hip fractures can present to this facility	86.1%	74.1%	0.46	0.166	2.80	0.003
This facility has an X-ray machine	97.2%	54.6%	0.94	0.166	5.64	<0.001
How many radiographers work in this facility	94.4%	51.7%	0.89	0.092	9.60	<0.001
This facility provides surgical treatment for a hip fracture	94.4%	65.3%	0.84	0.165	5.11	<0.001
**TBS (N repeats= 5, 15.3%)**						
Does your workplace have electricity?	80.0%	80.0%	0.00	0.000	.	.
Patients with hip fractures can present to this facility	100.0%	68.0%	1.00	0.447	2.24	0.013
Who was the main person who trained you to become a bone setter?	80.0%	52.0%	0.58	0.298	1.96	0.025
How many days in the week do you usually work as a bone setter?	100.0%	68.0%	1.00	0.447	2.24	0.013
Are you able to set hip (top quarter of thigh bone/femur) fractures?	100.0%	68.0%	1.00	0.447	2.24	0.013

Supplementary Information S1 Hospital service questionnaire

**SECTION 1: Identification**		
**Facility Identification**		
1.01	Data collection Start date:	[_][_]/[_][_]/[_][_] *(day/month/year)*
1.02	Country	The Gambia (1)<br>Zimbabwe (2)<br>South Africa (3)
1.03	Method of data collection	Face-to-face researcher administered questionnaire (1)<br>Virtual researcher administered questionnaire (2)<br>Online survey completed by respondent (3)<br>Paper form sent to facility (4)
1.03b	Researcher ID:<br>*(only to be asked if 1.03=1 or 1.03=2)*	________
We would like to know the location (latitude and longitude) of your facility. Please select from the three options below which method you would prefer to use for entering this.		
1.04	Method for reporting facility latitude C longitude:	(1) Enter latitude C longitude using maps.me (option for study team doing face to face data collection)<br>(2) Send location using WhatsApp (remote data collection - Android phone and internet connection needed)<br>(2) Enter latitude C longitude using google maps (remote data collection - computer, tablet or iphone needed)<br><br>*If 1.04=(2), continue to next question*<br>*If 1.04=(1 or 3), skip to 1.0c*
If 1.04=2:<br>To share the location of the facility with the study team via WhatsApp on your Android mobile phone, please follow these instructions:<br>a) Turn on wifi or mobile data and location (GPS) in your phone settings.<br>Swipe down from the top of your screen and tap the location symbol - when it turns blue it is switched on<br>b) Open WhatsApp and begin a new message to Study Team<br>*c)* Press the paperclip button, next to where you'd type a new message<br>Click 'Location' from the options (you may be prompted to allow WhatsApp to access your location, please allow this. You can change settings back afterwards).<br>d) By default your current location will be selected. If you are currently located at the facility (this is the preference), then click the 'Send your current location' button, then immediately send *another message with the ID number and name of the facility*<br>e) If you are not currently located at your facility but you know where it is on a map, press the button in the top left-hand corner (four corners of a square inside a circle), and drag the map to the location of your facility. You may need to zoom in or out to find the right place. Once the pin is in the right place, push the 'Send this location' button then *immediately send another message with the ID number and name of your facility*		
1.05	If 1.04=2<br>Once the location has been sent, please confirm this by clicking the 'Now' button (to the right) to enter the date C time of sending the facility location over WhatsApp	DD/MM/YYYY : HH:MM
If 1.04=1:<br>Instructions for entering Mapsme coordinates (study team doing in-person data collection, no internet connection needed):<br>a) Turn on location (GPS) in your device<br>b) Open the maps.me app<br>c) Press the blue arrow in the bottom right corner to navigate to your current location<br>d) Tap on the location on the map; the latitude and longitude coordinates will display (you may need to scroll down a little). These will be two numbers specified to over 5 decimal places<br>e) You can copy and paste this into the REDCap form by tapping and holding the number<br><br>Notes:<br>• The Maps.me App and The Gambia map within in it must be downloaded onto device in advance<br>• Tapping on the GPS coordinates displays them in different formats. Only copy the decimal degrees format e.g. 41.40338, 2.17403 and not degrees, minutes and seconds format e.g. 41°24'12.2"N 2°10'26.5"E		
If 1.04=3<br>Instructions for entering coordinates from Google maps (computer, tablet or iphone needed):<br>a) Turn on wifi or data mobile data, and location (GPS) in your phone, tablet or browser settings<br>b) Open the Google maps app or website<br>c) Either select your current location (if at a facility, the preference)<br>d) Or find and click to drop a pin where the facility is located<br>e) Please then click on the pin and the details that appear should include latitude and longitude co-ordinates. These will be two numbers inside two brackets, specified to over 5 decimal places. You will need to write these down and copy them into the questionnaire manually		
1.06a	Please enter facility's *latitude* (the first number from the coordinates):<br><br>*Please enter at least FOUR numbers after the decimal point. Take care to enter a negative sign ('-') if present:*	_ _ . _ _ _ _<br><br>Valid simple standard ranges: 13 to 14 (Gambia), -15 to -23 (Zimbabwe) and -22 to -35 (South Africa)

1.06b	Please enter facility's *longitude* (the second number from the coordinates:<br><br>*Please enter at least FOUR numbers after the decimal point. Take care to enter a negative sign ('-') if present:*		_ _ . _ _ _ _<br><br>Valid simple standard ranges: -13 to -17 (Gambia), 25 to 34 (Zimbabwe) and 16 to 33 (South Africa)
**Respondent Details**			
1.07	Name of main respondent completing questionnaire:<br>Enter First/Given Name followed by Surname/Family Name		
1.08	Main respondent's position in facility:		
1.09a	Main respondent's primary telephone number:<br>Include area code if applicable		
1.09b	Main respondent's secondary telephone number:<br>Include area code if applicable		
1.10a	Main respondent's primary email address:		
1.10b	Main respondent's secondary email address (optional):		
**MODULE 1: SERVICE AVAILABILITY**			
**SECTION 2: Staffing**			
How many staff with each of the following qualifications are currently assigned to, employed by, or seconded to this facility: (For the overall count, please count each staff member only once, based on their highest technical or professional qualification)			
2.01		Total number of doctors? (including junior, senior and specialist doctors)<br>Please enter 0 if none	[_][_][_]
2.02		How many of these doctors work part-time?<br>Please enter 0 if none	[_][_][_]
2.03a - 2.03g	(Several options may apply for each doctor)	How many of these doctors are specialists in:<br>(a.) general surgery? Please enter 0 if none<br>(b.) orthopaedic and trauma surgery? Please enter 0 if none<br>(c.) anaesthesia? Please enter 0 if none<br>(d.) general internal medicine? Please enter 0 if none<br>(e.) geriatric medicine? Please enter 0 if none<br>(f.) rheumatology? Please enter 0 if none<br>(g.) radiology? Please enter 0 if none	 [_][_][_]<br>[_][_][_]<br>[_][_][_]<br>[_][_][_]<br>[_][_][_]<br>[_][_][_]<br>[_][_][_]
2.04		TOTAL NUMBER of specialist doctors?<br>This is total number of doctors (people) that have at least one specialty training rather than the total number of specialties Please enter 0 if none	[_][_][_]
2.05		How many of these specialist doctors work part-time? Please enter 0 if none	[_][_][_]
2.06a - 2.06j	(Several options may apply)	Moving on from specialist doctors and thinking about other staff<br>How many of the staff working in this facility are:<br>(a.) clinical or medical officers? (also known as junior doctors) Please enter 0 if none<br>(b.) general nurses? Please enter 0 if none<br>(c.) orthopaedic trained nurses? Please enter 0 if none<br>(d.) healthcare assistants (not trained to nurse level)? These may be called nurse aids/assistants Please enter 0 if none<br>(e.) physiotherapists? Please enter 0 if none<br>(f.) occupational therapists? Please enter 0 if none<br>(g.) nutritionists/dieticians? Please enter 0 if none<br>(h.) radiographers? Please enter 0 if none<br>(i.) pharmacists? Please enter 0 if none<br>(j.) social workers? Please enter 0 if none	 [_][_][_]<br>[_][_][_]<br>[_][_][_]<br>[_][_][_]<br>[_][_][_]<br>[_][_][_]<br>[_][_][_]<br>[_][_][_]<br>[_][_][_]<br>[_][_][_]
2.07		TOTAL NUMBER of these members of staff:<br>For the total, each member of staff should be counted once only, even if they work in several disciplines. Please enter 0 if none	[_][_][_]
2.08		How many of these work part-time? Please enter 0 if none	[_][_][_]
**SECTION 3: Inpatient bed availability**			
3.01	How many overnight beds for adults does this facility have in total (excluding delivery beds in maternity)?		[_][_][_][_]
3.02	How many of the overnight beds are dedicated orthopaedic/trauma care beds for adults?		[_][_][_]
**MODULE 2: SERVICE READINESS (GENERAL)**			
**SECTION 4: Infrastructure**			
**Power Supply**			
4.01	Does your facility have electricity (from any source e.g. electricity grid, generator, solar) including for stand-alone devices (Expanded Programme on Immunisation [EPI] cold chain)?	Yes<br>No	1<br>0
Facilities with electricity only (If Q4.01=2, SKIP to Q4.0S)			

4.02	What is the electricity used for in this facility? Tick all that apply	Stand-alone electric medical devices/appliances<br>Electric lighting (excluding torches)<br>Communications (e.g. telephone, internet)<br>Other<br>Please specify other use	1<br>2<br>3<br>4
4.03	What is the facility's main source of electricity?	Central supply of electricity (e.g. national or community grid)<br>Generator/charged battery storage system<br>Solar system<br>Other<br>Please specify other source	1<br>2<br>3<br>4
4.04	Other than the main or primary source, does the facility have a secondary or backup source of electricity?	No secondary source<br>Central supply of electricity (e.g. national or community grid)<br>Generator charged battery storage system<br>Solar system<br>Other<br>Please specify other backup source	1<br>2<br>3<br>4<br>5
4.05	During the past 7 days, were there any interruptions to your main electricity supply when the facility was open?	Electricity always available without interruptions<br>Electricity often available (interruptions of less than two hours per day)<br>Electricity sometimes available (frequent or prolonged interruptions of more than two hours per day)<br>There was no electricity	1<br>2<br>3<br>4
If facility has a generator or battery storage system (charged by the grid) (Q4.03=2 and/or Q4.04=3), continue with Q4.0c. ***If not,*** *SKIP to Q4.08.*			
4.06	Is the generator or battery storage system functional?	Yes<br>No<br>Do not know	1<br>2<br>9
4.07	Is there fuel for the generator or a charged battery available today?	Yes<br>No<br>Do not know	1<br>2<br>9
If facility has a solar system (Q4.03=3 and/or Q4.04=4), continue with Q4.08. ***If not,*** *SKIP to Q4.0S.*			
4.08	Is the solar system functioning?	Yes<br>No<br>Do not know.	1<br>2<br>9
**Communications**			
4.09	Does this facility have a functioning landline telephone that is available to make calls outside the facility at all times that patient services are offered?<br>(If the facility is open for 24 hours, this service needs to be available for 24 hours).	Yes<br>No	1<br>0
4.10	Does this facility own a functioning mobile phone or a private mobile phone that is supported by the facility? The mobile phone can be privately owned but it should be functioning and supported by the facility.	Yes<br>No	1<br>0
4.11	Does this facility have a functioning shortwave radio to make calls?	Yes<br>No	1<br>0
4.12	Does this facility have a functioning computer?	Yes<br>No	1<br>0
4.13	When was it last possible to have access to email or internet from a facility-owned device within this facility?<br>A privately owned device counts, as long as the facility supports it by providing internet access.	Never<br>Today<br>Less than 1 month ago<br>At least 1 month ago<br>Do not know	1<br>2<br>3<br>4<br>9
**Ambulance/Emergency transport**			
4.14	Does this facility have a functional vehicle (e.g. an ambulance) for emergency transportation of patients, that is stationed at this facility or operates from this facility?	Yes<br>No	1<br>0
4.15	Does this facility have access to another vehicle for emergency transport of patients that is stationed at another facility or that operates from another facility nearby?	Yes<br>No	1<br>0
4.16	Have you got fuel for your ambulance/emergency vehicle today?<br>*If the facility has no access to any vehicles (Q4.14 AND Q4.15=2), SKIP to Q4.17*	Yes<br>No<br>Do not know	1<br>2<br>9

**Water**				
4.17	What is currently the most commonly used source of water for the facility?	Piped into/onto facility<br>Public tap/standpie<br>Tubewell/borehole<br>Dug well<br>Spring<br>Rainwater collection<br>Bottled water<br>Delivered by cart/truck<br>Surface water<br>Other<br>Please specify other source	1<br>2<br>3<br>4<br>5<br>6<br>7<br>8<br>9<br>10	*If the source is dug well (04) or spring (05), complete Q4.18, otherwise SKIP to Q4.1S*

4.18	Is the water source protected?	Yes<br>No<br>Do not know	1<br>2<br>9
**Basic client amenities**			
4.19	On average, how many hours per day is this facility open?		[_][_] hours
4.20	Is there a room with auditory and visual privacy available for patient consultations?	Auditory privacy only<br>Visual privacy only<br>Both auditory and visual privacy<br>No privacy	1<br>2<br>3<br>4
4.21	Is there a toilet on the premises in functioning condition that is accessible for general outpatient client use?<br>(if multiple toilets are available, consider the most modern type)	Flush toilet<br>Ventilated improved pit latrine (VIP)<br>Pit latrine with slab<br>Pit latrine without slab/ open pit<br>Composting toilet<br>Bucket<br>Hanging toilet/hanging latrine<br>No facilities on premises/bush/field	1<br>2<br>3<br>4<br>5<br>6<br>7<br>8
**Infection control**			
4.22	Does this facility have any guidelines on standard precautions for infection prevention?	Yes<br>No	1<br>0
**Healthcare waste management**			
4.23	We would like to ask you a few questions about waste management practices for medical waste, such as needles or blades and also for medical waste other than sharps, such as used bandages.<br>How does this facility finally dispose of medical waste?<br>Indicate all that apply	Burn incinerator: chamber industrial (800-1000 degrees C)<br>Burn incinerator: chamber drum/brick<br>Open burning: flat ground, no protection.<br>Open burning: pit or protected ground<br>Dump without burning: flat ground, open pit-no protection<br>Dump without burning: ground or pit with protection.<br>Remove offsite: stored in covered container<br>Remove offsite: stored in other protected environment<br>Remove offsite: stored unprotected<br>Other<br>Please specify other method used	1<br>2<br>3<br>4<br>5<br>6<br>7<br>8<br>9<br>10
**If incinerator used for waste disposal (Q4.23=01 or 02), continue with Q4.24<br>If not, SKIP to Q4.26**			
4.24	Is the incinerator functional today?	Yes<br>No<br>Don't know	1<br>2<br>9
4.25	Is fuel for the incinerator available today?	Yes<br>No<br>Don't know	1<br>2<br>9
**Processing of equipment for reuse**			
Are the following items for processing reusable equipment available and functional in this facility today?			
4.26a	Electric autoclave (pressure and wet heat)	Available and functional<br>Available but non-functional<br>Available but functionality unknown<br>Availability and functionality unknown<br>Not available	1<br>2<br>3<br>4<br>5
4.26b	If 4.26a=1-4, please indicate whether the availability/functionality in previous question was reported or observed<br>(*question only to be answered if 1.03=1*)		1, reported<br>2, observed
4.27a	Non-electric autoclave	Available and functional<br>Available but non-functional<br>Available but functionality unknown<br>Not available	1<br>2<br>3<br>4
4.27b	If 4.27a=1-4, please indicate whether the availability/functionality in previous question was reported or observed<br>(*question only to be answered if 1.03=1*)		1, reported<br>2, observed
4.28a	Electric dry heat sterilizer	Available and functional<br>Available but non-functional<br>Available but functionality unknown<br>Not available	1<br>2<br>3<br>4
4.28b	If 4.28a=1-4, please indicate whether the availability/functionality in previous question was reported or observed<br>(*question only to be answered if 1.03=1*)		1, reported<br>2, observed
4.29a	Electric boiler or steamer (no pressure)	Available and functional<br>Available but non-functional<br>Available but functionality unknown<br>Not available	1<br>2<br>3<br>4
4.29b	If 4.29a=1-4, please indicate whether the availability/functionality in previous question was reported or observed<br>(*question only to be answered if 1.03=1*)		1, reported<br>2, observed
4.30a	Non-electric pot with cover for boiling/steam	Available and functional<br>Availability and functionality unknown<br>Not available	1<br>2<br>3

4.30b	If 4.30a=1-4, please indicate whether the availability/functionality in previous question was reported or observed<br>(*question only to be answered if 1.03=1*)		1, reported<br>2, observed
4.31	Heat source for non-electric equipment<br>Only needs to be answered if non-electric equipment is used for sterilisation (4.27==1-4 OR 4.30==1-2)	Available and functional<br>Available but non-functional<br>Available but functionality unknown<br>Not available	1<br>2<br>3<br>4
**Supervision**			
4.32	When was the last time this facility received a supervision visit from the higher level (District Health Management Team or other)?	This month<br>In the last 3 months<br>More than 3 months ago<br>Do not know	1<br>2<br>3<br>9
**Section 5: General equipment**			
	Are the following equipment and supplies used in the provision of patient services available and functional in this facility today?		
5.01a	Adult weighing scales	Available and functional<br>Available but non-functional<br>Available but functionality unknown<br>Availability and functionality unknown<br>Not available	1<br>2<br>3<br>4<br>5
5.01b	If 5.01a=1-4, please indicate whether the availability/functionality in previous question was reported or observed<br>(*question only to be answered if 1.03=1*)		1, reported<br>2, observed
5.02a	Light source (a torch is acceptable)	Available and functional<br>Available but non-functional<br>Available but functionality unknown<br>Availability and functionality unknown<br>Not available	1<br>2<br>3<br>4<br>5
5.02b	If 5.02a=1-4, please indicate whether the availability/functionality in previous question was reported or observed<br>(*question only to be answered if 1.03=1*)		1, reported<br>2, observed
5.03a	Thermometer	Available and functional<br>Available but non-functional<br>Available but functionality unknown<br>Availability and functionality unknown<br>Not available	1<br>2<br>3<br>4<br>5
5.03b	If 5.03a=1-4, please indicate whether the availability/functionality in previous question was reported or observed<br>(*question only to be answered if 1.03=1*)		1, reported<br>2, observed
5.04a	Stethoscope	Available and functional<br>Available but non-functional<br>Available but functionality unknown<br>Availability and functionality unknown<br>Not available	1<br>2<br>3<br>4<br>5
5.04b	If 5.04a=1-4, please indicate whether the availability/functionality in previous question was reported or observed<br>(*question only to be answered if 1.03=1*)		1, reported<br>2, observed
5.05a	Blood pressure equipment (digital or manual sphygmomanometer with stethoscope)	Available and functional<br>Available but non-functional<br>Available but functionality unknown<br>Availability and functionality unknown<br>Not available	1<br>2<br>3<br>4<br>5
5.05b	If 5.05a=1-4, please indicate whether the availability/functionality in previous question was reported or observed<br>(*question only to be answered if 1.03=1*)		1, reported<br>2, observed
5.06a	Oxygen concentrators	Available and functional<br>Available but non-functional<br>Available but functionality unknown<br>Availability and functionality unknown<br>Not available	1<br>2<br>3<br>4<br>5
5.05b	If 5.05a=1-4, please indicate whether the availability/functionality in previous question was reported or observed<br>(*question only to be answered if 1.03=1*)		1, reported<br>2, observed
5.07a	Oxygen cylinders	Available and functional<br>Available but non-functional<br>Available but functionality unknown<br>Availability and functionality unknown<br>Not available	1<br>2<br>3<br>4<br>5
5.07b	If 5.07a=1-4, please indicate whether the availability/functionality in previous question was reported or observed<br>(*question only to be answered if 1.03=1*)		1, reported<br>2, observed
5.08a	Central oxygen supply	Available and functional	1

		Available but non-functional<br>Available but functionality unknown<br>Availability and functionality unknown<br>Not available	2<br>3<br>4<br>5
5.08b	If 5.08a=1-4, please indicate whether the availability/functionality in previous question was reported or observed<br>(*question only to be answered if 1.03=1*)		1, reported<br>2, observed
5.09a	Flowmeter for oxygen therapy (with humidification)	Available and functional<br>Available but non-functional<br>Available but functionality unknown<br>Availability and functionality unknown<br>Not available	1<br>2<br>3<br>4<br>5
5.09b	If 5.09a=1-4, please indicate whether the availability/functionality in previous question was reported or observed<br>(*question only to be answered if 1.03=1*)		1, reported<br>2, observed
5.10	At any time during the last three months has oxygen been unavailable for any reason?	Yes<br>No	1<br>0
**Infection Control Precautions**			
	Are the following resources/supplies used for infection control available in this facility today?		
5.11a	Clean running water (piped, bucket with tap, or pour pitcher)	1, Available<br>2, Availability unknown<br>3, Not available	
5.11b	If 5.11a=1-2, please indicate whether this was reported or observed<br>(*question only to be answered if 1.03=1*)		1, reported<br>2, observed
5.12a	Handwashing soap and/or liquid soap	1, Available<br>2, Availability unknown<br>3, Not available	
5.12b	If 5.12a=1-2, please indicate whether this was reported or observed<br>(*question only to be answered if 1.03=1*)		1, reported<br>2, observed
5.13a	Alcohol based hand rub	1, Available<br>2, Availability unknown<br>3, Not available	
5.13b	If 5.13a=1-2, please indicate whether this was reported or observed<br>(*question only to be answered if 1.03=1*)		1, reported<br>2, observed
5.14a	Disposable latex gloves	1, Available<br>2, Availability unknown<br>3, Not available	
5.14b	If 5.14a=1-2, please indicate whether this was reported or observed<br>(*question only to be answered if 1.03=1*)		1, reported<br>2, observed
5.15a	Waste receptacle (pedal bin) with lid and plastic bin liners	1, Available<br>2, Availability unknown<br>3, Not available	
5.15b	If 5.15a=1-2, please indicate whether this was reported or observed<br>(*question only to be answered if 1.03=1*)		1, reported<br>2, observed
5.16a	Sharps container (“safety box”)	1, Available<br>2, Availability unknown<br>3, Not available	
5.16b	If 5.16a=1-2, please indicate whether this was reported or observed<br>(*question only to be answered if 1.03=1*)		1, reported<br>2, observed
5.17a	Environmental disinfectant (e.g. chlorine, alcohol)	1, Available<br>2, Availability unknown<br>3, Not available	
5.17b	If 5.17a=1-2, please indicate whether this was reported or observed<br>(*question only to be answered if 1.03=1*)		1, reported<br>2, observed
5.18a	Disposable syringes with disposable needles	1, Available<br>2, Availability unknown<br>3, Not available	
5.18b	If 5.18a=1-2, please indicate whether this was reported or observed<br>(*question only to be answered if 1.03=1*)		1, reported<br>2, observed
**SECTION 6: Available Services**			
**Surgery**			

6.01	Does this facility offer any surgical services?<br>*If it does not offer surgical services (2), SKIP Qc.02-Qc.0c and continue with Qc.07*	Yes<br>No	1<br>0
	Are the following surgical equipment and supplies available and functional in this facility today.		
6.02a	Suture material (any type)	Available and functional<br>Available but non-functional<br>Available but functionality unknown<br>Availability and functionality unknown<br>Not available	1<br>2<br>3<br>4<br>5
6.02b	If 6.02a=1-4, please indicate whether the availability/functionality in previous question was reported or observed<br>(*question only to be answered if 1.03=1*)		1, reported<br>2, observed
6.03a	Skin disinfectant Examples: Povidone-Iodine, Chlorhexidine, alcohol-based solutions	Available and functional<br>Available but non-functional<br>Available but functionality unknown<br>Availability and functionality unknown<br>Not available	1<br>2<br>3<br>4<br>5
6.03a	If 6.03a=1-4, please indicate whether the availability/functionality in previous question was reported or observed<br>(*question only to be answered if 1.03=1*)		1, reported<br>2, observed
Anaesthetic drugs: Can be applied by inhalation or injection. The following list contains generic names (name of the actual substance) that is usually different from a brand (marketing) name.			
6.04a<br>6.04b	Anaesthetic drugs:<br>• Halothane (inhalation)<br>• Bupivacaine (injection)<br>• Lidocaine 5% (heavy spinal solution)<br>• Epinephrine (injection)<br>• Ephedrine (injection)	1, Available<br>2, Availability unknown<br>3, Not available<br><br>For each item that is available or unknown, indicate if this was reported or observed:<br>1, reported<br>2, observed	
6.05a	Do you have materials on Integrated Management of Emergency and Essential Surgical Care (IMEESC) (e.g. best practices, protocols, etc.) available in this facility today?	Yes<br>No	1<br>0
6.05b	If 6.05a=1, please indicate whether the availability of IMEESC materials was reported or observed<br>(*question only to be answered if 1.03=1*)		1, reported<br>2, observed
6.06	Have you or any provider(s) of basic surgical services received any training in IMEESC in the last two years?	Yes<br>No	1<br>0
**HIV counselling and testing**			
6.07	Does this facility offer HIV counselling and testing services?	Yes<br>No	1<br>0
**Blood transfusion**			
6.08	Does this facility offer blood transfusion services?<br>*If the facility does not offer transfusion services (2), SKIP to Qc.17*	Yes<br>No	1<br>0
6.09	Have there been any interruptions in blood availability during the past 3 months?	Yes<br>No	1<br>0
6.10	Does this facility obtain blood from a national or regional blood centre?	Yes<br>No	1<br>0
6.11	Is blood screened for infectious diseases prior to transfusion?<br>*If no blood screening offered (2), SKIP Qc.12 – Qc.15 and continue with Qc.1c*	Yes<br>No	1<br>0

		ALWAYS	SOMETIMES	NEVER
6.12 – 6.15	Is the blood that is transfused in the facility ALWAYS, SOMETIMES or NEVER screened for the following infections:			
	HIV	1	2	3
	Syphilis	1	2	3
	Hepatitis B	1	2	3
	Hepatitis C	1	2	3

6.16a	Does the blood transfusion service have a refrigerator available and functional for the storage of blood?	Available and functional<br>Available but non-functional<br>Available but functionality unknown<br>Availability and functionality unknown<br>Not available	1<br>2<br>3<br>4<br>5
6.16b	If 6.16a=1-4, please indicate whether the availability/functionality reported in previous question was reported or observed<br>(*question only to be answered if 1.03=1*)		1, reported<br>2, observed
6.17	Does the facility conduct haemoglobin testing onsite or offsite?	Yes, onsite<br>Yes, offsite<br>Neither	1<br>2<br>3

Radiography			
	Which imaging techniques are available in this facility?<br>*If any are available, ask to see them.*		
6.18a	Digital/non-digital radiography	Available and functional and there is a staff member trained/qualified to use it<br>Available and functional but there is not a staff member trained or qualified to use it<br>Available but non-functional<br>Available but functionality unknown<br>Availability and functionality unknown<br>Not available	1<br>2<br>3<br>4<br>5<br>6
6.18b	If 6.18a=1-5, please indicate whether the availability/functionality in previous question was reported or observed (*question only to be answered if 1.03=1*)		1, reported<br>2, observed
6.19a	CT scan (computer tomography)	Available and functional and there is a staff member trained/qualified to use it<br>Available and functional but there is not a staff member trained or qualified to use it<br>Available but non-functional<br>Available but functionality unknown<br>Not available	1<br>2<br>3<br>4<br>5
6.19b	If 6.19a=1-5, please indicate whether the availability/functionality in previous question was reported or observed (*question only to be answered if 1.03=1*)		1, reported<br>2, observed
6.20a	MRI (magnetic resonance imaging)	Available and functional and there is a staff member trained/qualified to use it<br>Available and functional but there is not a staff member trained or qualified to use it<br>Available but non-functional<br>Available but functionality unknown<br>Availability and functionality unknown<br>Not available	1<br>2<br>3<br>4<br>5<br>6
6.20b	If 6.20a=1-5, please indicate whether the availability/functionality in previous question was reported or observed (*question only to be answered if 1.03=1*)		1, reported<br>2, observed
6.21a	Fluoroscopy	Available and functional and there is a staff member trained/qualified to use it<br>Available and functional but there is not a staff member trained or qualified to use it<br>Available but non-functional<br>Available but functionality unknown<br>Availability and functionality unknown<br>Not available	1<br>2<br>3<br>4<br>5<br>6
6.21b	If 6.21a=1-5, please indicate whether the availability/functionality in previous question was reported or observed (*question only to be answered if 1.03=1*)		1, reported<br>2, observed

SECTION 7: Medication and Commodities						
Check the dates of the stored product						
	Does this facility stock the following medicines/equipment?(check the date of the stored product)<br><br>The brand (marketing) name may be different from the generic names used in this list. However, the generic name should be used under ingredients listed on the drug container.	Availability				
		Yes, at least one available and in date	Yes, but all out of date	Not today	Availability unknown	Never
7.01	Paracetamol	1	2	3	4	5
7.02	Codeine	1	2	3	4	5
7.03	Morphine	1	2	3	4	5
7.04	Alendronic Acid	1	2	3	4	5
7.05	Zoledronate	1	2	3	4	5
7.06	Calcium supplement tablets	1	2	3	4	5
7.07	Vitamin D supplements	1	2	3	4	5
7.08	Normal saline IV solution	1	2	3	4	5
7.09	Ringers lactate IV solution (Also known as Hartmann's solution)	1	2	3	4	5
7.10	5% dextrose IV solution	1	2	3	4	5
7.11	Skin disinfectant	1	2	3	4	5
7.12	Gowns	1	2	3	4	5
7.13	Eye protection (goggles or face shields)	1	2	3	4	5
7.14	Medical (surgical or procedural) masks	1	2	3	4	5
7.15	Absorbable suture material	1	2	3	4	5
7.16	Non-absorbable suture material	1	2	3	4	5
Supply Chain						

7.18a<br>7.18b	Who is the principal person responsible for managing the ordering of medical supplies at this facility?	Nurse<br>Clinical/Medical Officer<br>Pharmacy technician<br>Pharmacist<br>Medical assistant<br>Other<br>If 'Other'(above), please specify:			1<br>2<br>3<br>4<br>5<br>6
7.19<br>7.20<br>7.21a<br>7.21b	Which of the following mechanisms is used to determine this facility's resupply quantities?	Yes	No	Don't know	
	The facility itself (pull distribution system)<br>A higher-level facility (push distribution system)<br>Other (specify below)	1<br>1<br>1	2<br>2<br>2	3<br>3<br>3	
	Please specify an 'other' systems (above):				
7.22a<br>7.22b	How are this facility's resupply quantities determined?	Formula (any calculation)<br>Don't know<br>Other means (specify below)			1<br>2<br>3
	If 'other' (above), please specify:				
7.23a<br>7.23b	What is the main source of your routine pharmaceutical commodity supplies? i.e who is the direct supplier to your facility?	Formula (any calculation)<br>Joint medical stores<br>NGO/donors<br>Private sources<br>Other			1<br>2<br>3<br>4<br>5
	If 'other' (above), please specify:				
7.24a<br>7.24b<br>7.24c	For the most recent order, how long did it take between ordering and receiving products? Provide the number of months, weeks and days taken (if less than 1 month, enter 0 months but enter number of weeks and days. If less than 1 week enter 0 months and 0 weeks and just enter number of days)	[__][__]months[__][__]weeks[__]days			
**SECTION 8: Fracture care**					
8.01a	Which of the following are available for the treatment of patients with fractures? Tick all that apply<br>Splints (for limb fractures)<br>Slings (for arm fractures)<br>Plaster of Paris<br>Lower limb traction<br>Walking aids (e.g. crutches)<br>None of those available				 1<br>2<br>3<br>4<br>5<br>6
8.01b	If 8.01a=1-5, please indicate whether availability in previous question was reported or observed (*question only to be answered if 1.03=1*)				1, reported<br>2, observed
8.02	Do you have clinical guidelines on osteoporosis management?				1 Yes<br>0 No
8.03	Does this facility offer care for people with suspected hip fractures?<br>Select one option only<br>Diagnostic investigation and treatment<br>Diagnostic investigation and referral only<br>Referral only<br>If Q8.03= 1 or 2, eligible to complete hip fracture specific readiness questionnaire				 <br>1<br>2<br>3
8.04	If patients get referred for hip fracture care, where do you routinely refer them to? Please provide facility name and address				
8.05	Regarding management of suspected hip fracture patients, do you have a clinical guideline on (select all that apply):<br>Pain management<br>Diagnostic investigations<br>Referral for care<br>None of the above				 <br>1<br>2<br>3<br>4
**You have now completed all of the questions in this survey. Thank you for your participation. If you have any comments to the survey in general or to specific questions, please tell us in section G.**					
**SECTION G: Researcher's/respondent's observations**					
9.01	General comments				
9.02	Comments on specific questions				
9.03	Data Collection: End date	[_][_]/[_][_]/[_][_] (*day/month/year*)			

Supplementary Information S2 Fracture service questionnaire

**SECTION 1: Identification**		
**Facility**		
1.01	Data collection start date	[_][_]/[_][_]/[_][_] (day/month/year)
1.02	Country<br>The Gambia<br>Zimbabwe<br>South Africa	 1<br>2<br>3
	Is this form being completed by an Orthopaedic surgeon?<br>Yes	 1
1.03	Method of data collection<br>Face-to-face researcher administered questionnaire<br>Virtual researcher administered questionnaire<br>Online survey completed by respondent<br>Paper form sent to facility (4)	 1<br>2<br>3
1.03b	Researcher ID<br>*(only to be asked if 1.03=1 or 1.03=2)*	__________
**Respondent**		
1.04	Name of main respondent completing questionnaire	
1.05	Main respondent's position in facility	
1.06a	Main respondent's primary telephone number: (Include area code if applicable)	
1.06b	Main respondent's secondary telephone number (optional): (Include area code if applicable)	
1.07a	Main respondent's primary email address:	
1.07b	Main respondent's secondary email address (optional):	
**HIP FRACTURE SERVICE**		
**SECTION 2: General Information**		
2.01	Does this facility have clinical guidelines on Osteoporosis management?<br>Yes<br>No	 1<br>0
2.02	Regarding management of suspected hip fracture patients, does this facility have a clinical guideline on: (select all that apply)<br>Pain management<br>Diagnostic investigations<br>Referral for care<br>None of the above	 1<br>2<br>3<br>4
2.03	Does this facility offer care for people with suspected hip fractures?<br>Diagnostic investigation and treatment<br>Diagnostic investigation and referral only<br>Referral only	 1<br>2<br>3
If the facility offers diagnostic investigation and referral only (Q2.03=2) SKIP to Q2.05.<br>If Q2.03=3 END the questionnaire.		
2.04	Does this facility provide: Select all that apply<br>Non-surgical treatment (e.g. traction) for a hip fracture<br>Surgical treatment for a hip fracture	 1<br>2
2.05	How many days per week is this facility open to receive people with suspected hip fractures?	[__] day(s)
2.06	On the days when the facility is open, how many hours per day is this facility open to receive people with suspected hip fractures?	[__][__] hours
2.07	Which of the following staff members are present in the facility or on call over 24 hours (including weekends and on public holidays)?<br>Select all that apply<br>General surgeon<br>Orthopaedic/ trauma surgeon<br>Orthopaedic trained nurse<br>Anaesthetist<br>Operating theatre assistants<br>Radiologist<br>Radiographers<br>Laboratory technicians<br>None of the above	 <br><br>1<br>2<br>3<br>4<br>5<br>6<br>7<br>8<br>9
The following questions relate to people with a hip fracture who attend your facility for care.<br>Please use written records if available to answer Q2.08 to Q2.1S. If not, use estimates. Please indicate whether the figures are based on records or estimates , when prompted.		
2.08a<br>2.08b	How many people with a hip fracture attended this facility in 2020?	[__][__][__]
2.08b	Please indicate if this is based on records or estimates:<br>Records<br>Estimates	 1<br>2
2.08c	How many people with a hip fracture attended this facility in 2022?<br>(Only asked for repeated facilities)	[__][__][__]
2.08d	Please indicate if this is based on records or estimates:<br>Records<br>Estimates	 1<br>2
2.09a	Of those hip fractures in 2020, what percentage were caused by a low impact injury (such as falling over)?	[__][__][__]%

2.09b	Please indicate if this is based on records or estimates:	Records<br>Estimates	1<br>2
2.09c	Of those hip fractures in 2022, what percentage were caused by a low impact injury (such as falling over)?<br>(Only asked for repeated facilities)		[__][__][__]%
2.09d	Please indicate if this is based on records or estimates:	Records<br>Estimates	1<br>2
2.10a	<u>In 2020,</u> what percentage of all the people with hip fractures had been referred to your hospital from another health facility?		[__][__][__]%
2.10b	Please indicate if this is based on records or estimates:	Records<br>Estimates	1<br>2
2.10c	<u>In 2022,</u> what percentage of all the people with hip fractures had been referred to your hospital from another health facility?<br>(Only asked for repeated facilities)		[__][__][__]%
2.10d	Please indicate if this is based on records or estimates:	Records<br>Estimates	1<br>2
2.11a	Over the last 4 weeks, how many people with a hip fracture have attended this facility?		[__][__][__]
2.11b	Please indicate if this is based on records or estimates:	Records<br>Estimates	1<br>2
2.12a	Of those hip fractures in the last 4 weeks, what percentage were caused by a low impact injury (such as falling over)?		[__][__][__]%
2.12b	Please indicate if this is based on records or estimates:	Records<br>Estimates	1<br>2
2.13a	Over the last 4 weeks, what percentage of all the people with hip fractures had been referred to your hospital from another health facility?		[__][__][__]%
2.13b	Please indicate if this is based on records or estimates	Records<br>Estimates	1<br>2
**SECTION 3: Pathways of hip fracture care**			
**Before arrival to your health facility**			
3.01	Whom would a patient most commonly contact for help if they injure their hip and cannot bear weight? Select up to 3 responses	Family<br>Friends/neighbours<br>Hospital<br>Health Centre/Clinic<br>Community Health worker/nurse<br>Pharmacy/chemist/Dispensary<br>Community based organisation (CBO) or non-governmental organisation (NGO)<br>Traditional healers/bonesetters<br>Religious leader/church<br>Private doctor/clinic<br>Other (specify)<br>Do not know	1<br>2<br>3<br>4<br>5<br>6<br>7<br>8<br>9<br>10<br>11<br>12
		Please specify 'other'	
3.02	How would a person with a suspected hip fracture most commonly be transported to your facility?	Ambulance<br>Private transport<br>Public transport<br>Other (specify)<br>Do not know	1<br>2<br>3<br>4<br>5
		Please specify 'other'	
3.03	On average, how long do you estimate it takes for a patient with a suspected hip fracture to reach your facility after the injury has occurred: Please answer in either days or hours (below)<br>Number of days: enter 0 if less than one day, and provide answer in hours (below)<br>Number of hours: enter 0 if wishing to provide answer only in days		[__][__]days<br>[__][__]hours
3.04	Has a person with a suspected hip fracture usually received pain medication before they arrive at your facility? If so, which one(s)? (several options may apply)	No<br>Simple painkillers (e.g. paracetamol)<br>Oral opiates<br>Injected opiates<br>Other (specify below)<br>Do not know	1<br>2<br>3<br>4<br>5<br>6
		Please specify 'other' pain medication (reported above)	

Within your health facility			
3.05	On arrival at your facility, who is the person with a suspected hip fracture assessed by? (several may apply)		
	Nurse		1
	Clinical Officer		2
	General doctor		3
	Emergency department doctor		4
	General surgical doctor		5
	Orthopaedic doctor		6
	Geriatric medicine doctor		7
	Internal medicine doctor		8
	Anaesthetist		9
	Other (specify)		10
	Please specify 'other' (as reported above)		
3.06	On arrival at your facility, what pain relief is a person with a suspected hip fracture offered? (several may apply)		
	Simple painkillers such as paracetamol		1
	Oral opiates		2
	Intramuscular injection of opiates		3
	Intravenous injection of opiates		4
	Nerve block (e.g. of the femoral nerve)		5
	Other (specify below)		6
	Please specify 'other'		
3.07	Which is the first imaging technique offered to a person with suspected hip fracture? (several may apply)		
	Anterior-posterior (AP) radiograph of the pelvis		1
	AP radiograph of the hip		2
	Lateral radiograph of the hip		3
	Other (specify below)		4
	Please specify 'other' (as reported above)		
3.08	Is traction (skin or skeletal) routinely applied to the injured leg?		
	Yes		1
	No		0
3.09	What is the average waiting time in hours from arrival at your facility to admission to the ward? If less than 1 hour, fill in 00		[__][__] hours
	Skip this question if clinical facility does not admit patients with hip fractures (Q2.03=2)		
3.10	What is the average waiting time in hours from arrival at your facility to onward referral for hip fracture care? If less than 1 hour, fill in 00		[__][__] hours
	Skip this question if clinical facility admits patients with hip fractures (Q2.03=1)		
	Do you have guidelines on the following: Please send a copy of available guidelines to study team		Has a copy of the guideline sent/given to the study team?
3.11	Osteoporosis management?	1 Yes/ 0 No	1 Yes / 0 No
3.12	Management of suspected hip fracture patients regarding pain management?	1 Yes/ 0 No	1 Yes/ 0 No
3.13	Management of suspected hip fracture patients regarding diagnostic investigations?	1 Yes/ 0 No	1 Yes/ 0 No
3.14	Management of suspected hip fracture patients regarding referral for care?	1 Yes/ 0 No	1 Yes/ 0 No
3.15	None of the above guidelines?	We don't have any of the above guidelines	---------
	Copies of guidelines should be emailed to local research assistant		
Complete this section only if facility provides surgical treatment for hip fractures *(Q2.04=2, otherwise SKIP to Q4.04*			
3.16	Who routinely assesses the patient on the ward before surgery for a hip fracture? (several may apply)		
	Clinical Officer		1
	General surgeon		2
	Orthopaedic surgeon		3
	Geriatric medicine doctor		4
	Internal medicine doctor		5
	Anaesthetist		6
	Other (specify)		7
	If 'other' (above), please specify		
3.17a	Do patients with a hip fracture have a routine assessment of their cognitive function before surgery?		
	Yes, 10 question Abbreviated Mini-mental Test (AMT)		1
	Yes, 4AT		2
	No		3
	Other		4
3.17b	Please specify type of 'other' (reported above)		
3.18	What is the average time in days between presenting to hospital and performing surgery for a hip fracture? If less than 1, enter 00		[__][__] days
3.19	What percentage of patients with hip fractures receive surgery in your facility?		[__][__][__]%
	How important are the following factors when deciding whether a patient will receive surgery for a hip fracture:For each factor, use a scale from 1 to 10 with 1 being "not at all important" and 10 being "extremely important"		
3.20	Patient's health?		[__][__]
3.21	Patient's age?		[__][__]

3.22<br>3.23<br>3.24<br>3.25<br>3.26<br>3.27<br>3.28a<br>3.28b	Patient's wishes?<br>Family's wishes?<br>Patient's ability to pay for the operation?<br>Availability of equipment<br>Availability of surgical expertise?<br>Availability of anaesthetic expertise?<br>Are there any other factors, if so please specify these factor/s: specify factors, or leave blank if there are no others<br>If there are other factor/s, please specify their importance	[__][__]<br>[__][__]<br>[__][__]<br>[__][__]<br>[__][__]<br>[__][__]<br>[__][__]
3.29	What percentage of patients who receive surgery for their hip fracture has the operation done under general anaesthesia? percentage	[__][__][__]%
3.30	What percentage of patients who receive surgery for their hip fracture has the operation done under spinal anaesthesia? percentage	[__][__][__]%
3.31	What percentage of patients gets a regional nerve block (e.g. of the femoral nerve) as part of their anaesthetic?? percentage	[__][__][__]%
3.32	Who usually gives the anaesthesia for hip fracture surgery in your facility?<br>A fully qualified anaesthetist<br>An anaesthetist in training<br>An anaesthetic assistant<br>Other (specify)	 1<br>2<br>3<br>4
	Please specify 'other' (as selected above)	
3.33	Which of the following hip fracture fixation prostheses are available in hospital stock for use in your hospital today? (several may apply)<br>Total hip replacement (THR)<br>Hip hemi-arthroplasty<br>IM (intramedullary) nail<br>Cannulated screws<br>Sliding hip screw<br>None of those available	 1<br>2<br>3<br>4<br>5<br>6
3.34	For patients with an undisplaced intra-capsular fracture, what is your hospital's preferred surgical option<br>Screw fixation<br>Sliding hip screw fixation<br>Hemi-arthroplasty cemented<br>Hemi-arthroplasty uncemented<br>Total hip replacement (THR)<br>No standardisation<br>Other (specify)<br>Do not know	 1<br>2<br>3<br>4<br>5<br>6<br>7<br>8
	No standardisation – further detail	
	Please specify 'other' (as selected above)	
3.35	For patients with a displaced intra-capsular fracture, what is your hospital's preferred surgical option<br>Screw fixation<br>Sliding hip screw fixation<br>Hemi-arthroplasty cemented<br>Hemi-arthroplasty uncemented<br>Total hip replacement (THR)<br>No standardisation<br>Other (specify)<br>Do not know	 1<br>2<br>3<br>4<br>5<br>6<br>7<br>8
	No standardisation – further detail	
	Please specify 'other' (as selected above)	
3.36	Are patients with good mobility before hip fracture routinely offered a total hip replacement?<br>Yes, cemented<br>Yes, uncemented<br>Yes, either cemented or uncemented (i.e. according to surgeon's preference)<br>No<br>Do not know	 1<br>2<br>3<br><br>4<br>5
3.37	For patients with a two-part extra-capsular fracture, what is your hospital's preferred surgical option:<br>IM (intramedullary) nail fixation<br>Sliding hip screw fixation<br>Either IM nail fixation or sliding hip screw fixation (i.e. according to surgeon's preference)<br>Other (specify)<br>Do not know	 1<br>2<br>3<br><br>4<br>5
	Please specify 'other' (as selected above)	
3.38	For patients with a complex (more than two-part) extra-capsular fracture, what is your hospital's preferred surgical option:<br>IM (intramedullary) nail fixation<br>Sliding hip screw fixation<br>Either IM nail fixation or sliding hip screw fixation (i.e. according to surgeon's preference)<br>Other (specify)<br>Do not know	 1<br>2<br>3<br>4<br>5
	Please specify 'other' (as selected above)	

3.39	For patients with a reverse oblique (lateral wall) extra-capsular fracture, what is your hospital's preferred surgical option:<br>IM (intramedullary) nail fixation<br>Sliding hip screw fixation<br>Either nail fixation or sliding hip screw fixation (i.e. according to surgeon's preference)<br>Other (specify)<br>Do not know	 1<br>2<br>3<br>4<br>5
	Please specify 'other' (as selected above)	
3.40	What wards do patients go to after hip fracture surgery? (indicate all that apply)<br>General ward<br>Surgical ward<br>Geriatric ward<br>Specialist orthopaedic ward<br>Other (specify below)	 1<br>2<br>3<br>4<br>5
	Please specify 'other' (as selected above)	
3.41	Which doctors are routinely involved in the care of patients with hip fractures whilst in your facility: (indicate all that apply)<br>Anaesthetists<br>Orthopaedic surgeons<br>Geriatricians<br>General internal medics<br>Palliative care doctors<br>Rehabilitation doctors<br>Other (specify below)	 1<br>2<br>3<br>4<br>5<br>6<br>7
	Please specify 'other' (as selected above)	
3.42	Which of the following staff are routinely involved in the care of patients with hip fractures whilst in your facility: (indicate all that apply)<br>General nurses<br>Specialist orthopaedic nurses<br>Physiotherapists<br>Occupational therapists<br>Dietitians<br>Pharmacists<br>Social workers<br>Case managers<br>Other (specify below)	 1<br>2<br>3<br>4<br>5<br>6<br>7<br>8<br>9
	Please specify 'other' (as selected above)	
	What percentage of patients with hip fractures develop the following complications whilst they are in your facility:	
3.43	Pressure sores	[__][__][__]%
3.44	a DVT or PE	[__][__][__]%
3.45	post-operative wound infection	[__][__][__]%
3.46	Other post-operative wound infections, including pneumonia and urinary infection	[__][__][__]%
3.47	What percentage of patients in your facility are routinely allowed to put their full weight on their injured leg in the first 24 hours after surgery? Fill in 999 if you do not know	[__][__][__]%
 <br>3.48<br>3.49<br><br>3.50	How many days after surgery are patients allowed to put their full weight on their operated leg?<br>After arthroplasty Fill in 99 if not used in your facility<br>After an internal fixation of an intracapsular hip fracture Fill in 99 if not used in your facility<br>After an internal fixation of an extracapsular hip fracture Fill in 99 if not used in your facility	 <br>[__][__]days<br>[__][__]days<br>[__][__]days
3.51	If you would like to provide more information on weight bearing post-surgery, please do so here: Fill in 99 if not used in your facility	
3.52	How many days after surgery do you routinely start mobilising patients? Fill in 99 if not used in your facility	[__][__]days
3.53	Who helps the patients to mobilise?<br>(select all that apply)<br>General Nurse<br>Orthopaedic nurse<br>Physiotherapist<br>Occupational therapist<br>Family<br>Other (specify)	 <br>1<br>2<br>3<br>4<br>5<br>6
	please specify 'other' (as answered above):	

3.54a	What equipment do you routinely have available to help mobilise patients? (select all that apply)<br>Walking sticks<br>Crutches<br>Walking (Zimmer) frame<br>Pulpit frame<br>Sara Stedy™<br>Wheelchair<br>Hoist<br>Other (specify)	 <br>1<br>2<br>3<br>4<br>5<br>6<br>7<br>8
3.54b	please specify 'other' (as answered above):	
 <br>3.55<br>3.56<br>3.57<br>3.58<br>3.59<br>3.60	What equipment are you able to provide for patients at discharge to take home:<br><br>Walking sticks<br>Crutches<br>Walking (Zimmer) frame<br>Wheelchair<br>Hoist<br>Other	 Response options:<br>1 Available at no cost to patient<br>2 Available to patient if they pay<br>3 Not available
	please specify 'other' equipment (as answered above)	
3.61	On average, how many days do people with a hip fracture who have an operation stay in your facility?	[__][__][__] days
3.62	On average, how many days do people with a hip fracture who DO NOT have an operation stay in your facility?	[__][__][__] days
3.63	If indicated, what medication do you prescribe for osteoporosis before discharge from your facility? (indicate all that apply)<br>Calcium and vitamin D supplements<br>Alendronic acid (oral tablets)<br>Risedronate (oral tablets)<br>Ibandronic acid (oral tablets)<br>Zoledronate (intravenous)<br>Hormone replacement therapy (for women)<br>Raloxifine (oral tablets)<br>Strontium ranelate (oral sachets)<br>Teriparatide (subcutaneous injections)	 <br>1<br>2<br>3<br>4<br>5<br>6<br>7<br>8<br>9
3.64	Which of the following investigations do you use to identify people who need these medications for osteoporosis? (indicate all that apply)<br>X-ray<br>DXA scan (of hip and lumbar spine)<br>DXA VFA scan (vertebral fracture assessment)<br>CT-scan<br>MRI<br>Blood tests for bone turnover markers<br>Fracture risk assessment tool (e.g. FRAX)<br>Other (specify)<br>None	 <br>1<br>2<br>3<br>4<br>5<br>6<br>7<br>8<br>9
3.65	Is a DXA scanner available in this facility?<br>Available and functional<br>Available but non-functional<br>Available but functionality unknown<br>Not available	 1<br>2<br>3<br>4
**SECTION 4: Guidelines and protocols for hip fracture care**		
Please send copies of guidelines/protocols to study team if available		
4.01	For hip fracture patients, do you have:<br>(indicate all that apply, and please send the study team a copy of guidelines/protocols if available)<br><br>1 A written treatment plan for care before hospital (e.g. ambulance care)<br>2 Standardised admission paperwork (e.g. hip fracture admission proforma)<br>3 Protocol/ policy for pain management before surgery<br>4 Protocol/ policy for pain management after surgery<br>5 Standardised assessment of readiness to be discharged<br>6 Written treatment plan for after discharge<br>7 Local guideline for osteoporosis management<br>8 None of the above	Copy sent to study team if available<br><br><br>(a) 1 Yes/ 0 No<br>(b) 1 Yes/ 0No<br>(c) 1 Yes/ 0 No<br>(d) 1 Yes/ 0 No<br>(e) 1 Yes/ 0 No<br>(f) 1 Yes/ 0 No<br>(g) 1 Yes/ 0 No
	Copies of guidelines should be emailed to local research assistant	
4.02	Do you routinely collect data on patients with a hip fracture after they have been discharged?<br>Yes<br>No<br>If not (4.02=2), SKIP to 4.04	 1<br>0
4.03a	What data do you routinely collect after discharge? (indicate all that apply)<br>Death<br>Cause of death<br>Re-admission for hip fracture/surgery related problem<br>Re-operation	 1<br>2<br>3<br>4

4.03b		Other (specify)	5
Please specify 'other' (reported above)			
4.04	Data collection: End date	[_][_]/[_][_]/[_][_] (*day/month/year)*	
You have now completed all the questions of this survey. Thank you very much for your participation.			

Supplementary Information S3 TBS questionnaire

**SECTION 1: IDENTIFICATION**		
1.01	Data collection start date<br>*Full Date must be entered*	[_][_]/[_][_]/[_][_] (*day/month/year*)
1.02	Country<br>The Gambia	1
1.03a	Method of data collection<br>Face-to-face researcher-administered questionnaire<br>Virtual researcher-administered questionnaire<br>Online survey completed by respondent	1<br>2<br>3
1.03b	Interviewer<br>*(only to be asked if 1.03a=1 or 1.03a=2)*	
**Geographic Coordinates**		
We would like to know the location (latitude and longitude) of your facility. Please select from the two options below which method you would prefer to use for entering these geographical coordinates.		
1.04	Method for reporting facility latitude C longitude	(1) Enter latitude C longitude using maps.me (option for study team doing face to face data collection)<br>(2) Send location using WhatsApp (remote data collection - Android phone and internet connection needed)<br>(3) Enter latitude C longitude using google maps (remote data collection - computer, tablet or iphone needed)<br>*If 1.04=(1), continue to next question, If 1.04=(2), skip to 1.0c*

If 1.04=2:
To share the location of the facility with the study team via WhatsApp on your Android mobile phone, please follow these instructions:

f) Turn on wifi or mobile data and location (GPS) in your phone settings
Swipe down from the top of your screen and tap the location symbol – when it turns blue it is switched on
g) Open WhatsApp and begin a new message to Study Team
h) Press the paperclip button, next to where you'd type a new message
Click 'Location' from the options *(you may be prompted to allow WhatsApp to access your location, please allow this. You can change settings back afterwards).*
i) By default your current location will be selected. If you are currently located at the facility (this is the preference), then click the 'Send your current location' button, then immediately send another message with the ID number and name of the facility
j) If you are not currently located at your facility but you know where it is on a map, press the button in the top left-hand corner (four corners of a square inside a circle), and drag the map to the location of your facility. You may need to zoom in or out to find the right place. Once the pin is in the right place, push the 'Send this location' button then immediately send another message with the ID number and name of your facility

1.05	If 1.04=2<br>for confirmation purposes, please enter date and time of sending the facility location<br>*please click the 'Now' button (to the right) to enter the date & time of sending the facility location over WhatsApp*	DD/MM/YYYY : HH:MM

If 1.04=1:
Instructions for sending Mapsme coordinates (study team doing in-person data collection, no internet connection needed):

f) Turn on location (GPS) in your device
g) Open the maps.me app
h) Press the blue arrow in the bottom right corner to navigate to your current location
i) Tap on the location on the map; the latitude and longitude coordinates will display (you may need to scroll down a little). These will be two numbers specified to over 5 decimal places
j) You can copy and paste this into the REDCap form by tapping and holding the number

Notes:

- the Maps.me App and The Gambia map within in it must be downloaded onto device in advance

Tapping on the GPS coordinates displays them in different formats. Only copy the decimal degrees format e.g. 41.40338, 2.17403 and not degrees, minutes and seconds format e.g. 41°24'12.2"N 2°10'26.5"E

If 1.04=3
Instructions for sending coordinates using Google maps (computer, tablet or iphone needed):

f) Turn on wifi or data mobile data, and location (GPS) in your phone, tablet or browser settings
g) Open the Google maps app or website
h) Either select your current location (if at a facility, the preference)
i) Or find and click to drop a pin where the facility is located
j) Please then click on the pin and the details that appear should include latitude and longitude co-ordinates. These will be two numbers inside two brackets, specified to over 5 decimal places. You will need to write these down and copy them into the questionnaire manually

1.06a	Please enter facility's latitude (the first number from the coordinates):<br><br>Please enter at least FOUR numbers after the decimal point. Take care to enter a negative sign ('-') if present:	_ _ . _ _ _ _<br><br>*Valid simple standard ranges: 13 to 14 (Gambia),*
1.06b	Please enter facility's longitude (the second number from the coordinates):	_ _ . _ _ _ _

	Please enter at least FOUR numbers after the decimal point. Take care to enter a negative sign ('-') if present:	*Valid simple standard ranges: -13 to -17 (Gambia)*	
**SECTION 2: Background and workplace**			
2.01	How many years have you worked as a bone setter?	[_][_] *(years)*<br>*00 if less than 1 year*	
2.02a	Who was the main person who trained you to become a bone setter?	Another bone setter from my family<br>Another local bone setter<br>Another bone setter further away<br>I trained myself<br>Other	1<br>2<br>3<br>4<br>5
2.02b	Other (specify)		
2.03	In which regions have you worked as a bone setter?	Lower River Region (LRR)<br>Central River Region (CRR)<br>North Bank Region (NBR)<br>Upper River Region (URR)<br>West Coast Region (WCR)<br>Banjul City Council (BCC)<br>Kanifing Municipal Council (KMC)	1<br>2<br>3<br>4<br>5<br>6<br>7
2.04	How many days in the week do you usually work as a bone setter?	Less than 1 day<br>1 day<br>2 days<br>3 days<br>4 days<br>5 days<br>6 days<br>7 days	0<br>1<br>2<br>3<br>4<br>5<br>6<br>7
2.05	Where do you mainly work from as a bone setter?	From my home<br>From a separate work facility<br>From the homes of clients who I visit	1<br>2<br>3
2.06	If working from home or in a separate facility, do you have beds where patients can stay overnight if needed?		1Yes<br>0 No
2.08	How many people work with you?	*If the bone setter works alone, skip to Q.3.01*	[_][_]
2.09a	How many of the people you work with are?	Fully trained independent bone setters<br>Trainee bone setters<br>Assistants<br>Others	0/1/2/3/4<br>0/1/2/3/4<br>0/1/2/3/4<br>0/1/2/3/4
2.09b Others (specify)			
**SECTION 3: Fracture care**			
	What kind of injuries commonly present to you?		
3.01	Fingers/hands	Never/Rare/Sometimes/Common	1/2/3/4
3.02	Arms (skin intact)	Never/Rare/Sometimes/Common	1/2/3/4
3.03	Arms (skin broken)	Never/Rare/Sometimes/Common	1/2/3/4
3.04	Hip (top quarter of thigh bone/femur)	Never/Rare/Sometimes/Common	1/2/3/4
3.05	Legs (excluding hips, skin intact)	Never/Rare/Sometimes/Common	1/2/3/4
3.06	Legs (excluding hips, skin broken)	Never/Rare/Sometimes/Common	1/2/3/4
3.07	Toes/feet	Never/Rare/Sometimes/Common	1/2/3/4
3.08	Face/nose	Never/Rare/Sometimes/Common	1/2/3/4
3.09	Neck	Never/Rare/Sometimes/Common	1/2/3/4
3.10	Spine	Never/Rare/Sometimes/Common	1/2/3/4
3.11a	Other	Never/Rare/Sometimes/Common	1/2/3/4
3.11b	Other Specify		
3.12a	For what kind of injuries do you suggest the patient has an X-ray? (Several may apply) *If Other, please Other Specify must not be blank*	Fingers/hands<br>Arms (skin intact)<br>Arms (skin broken)<br>Hip (top quarter of thigh bone/femur)<br>Legs (excluding hips, skin intact)<br>Legs (excluding hips, skin broken)<br>Toes/feet<br>Face/nose<br>Neck<br>Spine<br>Other	1<br>2<br>3<br>4<br>5<br>6<br>7<br>8<br>9<br>10<br>11
3.12b		Other (specify)	

3.13a	What kind of fractures are you able to set? (Several may apply)<br>*If Other, please Other Specify must not be blank*		
	Fingers/hands		1
	Arms (skin intact)		2
	Arms (skin broken)		3
	Hip (top quarter of thigh bone/femur)		4
	Legs (excluding hips, skin intact)		5
	Legs (excluding hips, skin broken)		6
	Toes/feet		7
	Face/nose		8
	Neck		9
	Spine		10
	Other		11
3.13b	Other specify		
	Which of the following do you use for pain relief when treating patients with fractures?		
3.14	Paracetamol	Never/Rare/Sometimes/Often	1/2/3/4
3.15	Ibuprofen/Diclofenac/Naproxen	Never/Rare/Sometimes/Often	1/2/3/4
3.16	Codeine	Never/Rare/Sometimes/Often	1/2/3/4
3.17	Morphine	Never/Rare/Sometimes/Often	1/2/3/4
3.18	Traditional medication and/or herbs	Never/Rare/Sometimes/Often	1/2/3/4
3.19	Alcohol	Never/Rare/Sometimes/Often	1/2/3/4
3.20	None	Never/Rare/Sometimes/Often	1/2/3/4
3.21a<br>3.21b	Other<br>Other (specify)	Never/Rare/Sometimes/Often	1/2/3/4
3.22	If you use traditional medication and/or herbs, please specify which ones		Show if 3.18 = 2/3/4
3.23	Do you refer patients onwards if they have a fracture that you cannot set? (Several options may apply)		
	Yes, to another bone setter		1
	Yes, to a clinical facility (provide details)		2
	No		3
xx_warning1	WARNING: please check responses. If 'No' is ticked alongside any of the other options then please amend		Show if 3.23 =3 and 1 or 2
3.24	Do you keep records of the number and type of fractures that you treat?<br>Yes, number only<br>Yes, number and type<br>No		 <br>1<br>2<br>3
3.25a<br>3.25b	How many fractures did you treat over the last 4 weeks<br>Please indicate if this is based on records or estimates:		[] [] []<br>1 Recorded<br>2 Estimated
3.25c<br>3.25d	How many of those were hip fractures?<br>Please indicate if this is based on records or estimates:		[] [] []<br>1 Recorded<br>2 Estimated
3.26a<br>3.26b	How many fractures did you treat in the last year<br>Please indicate if this is based on records or estimates:		[] [] []<br>1 Recorded<br>2 Estimated
3.26c<br>3.26d	How many were hip fractures?<br>Please indicate if this is based on records or estimates:		[] [] []<br>1 Recorded<br>2 Estimated
3.27a<br>3.27b	How many of these hip fractures were confirmed (to your knowledge) by Xray<br>Please indicate if this is based on records or estimates		[] [] []<br>1 Recorded<br>2 Estimated
**Hip Fractures**			
3.31a	Before arrival with you, whom would a patient most commonly contact for help if they injure their hip and cannot bear weight?<br>*If Other, please Other Specify must not be blank*	Family/friends/neighbours	1
		Hospital	2
		Health Centre/Clinic	3
		Community Health worker/nurse	4
		Pharmacy/chemist/Dispensary	5
		Community-based organisation (CBO) or non-governmental organisation (NGO)	6
		Another traditional bone setters	7
		Religious leader/church/mosque	8
		Private doctor/clinic	9
		Other	10

3.231b	Do not know<br>Other specify	11
3.32a<br><br><br><br>3.32b	How long does it usually take for a patient with a suspected hip fracture to reach you after the injury has occurred? (in Days) If they usually present on the same day of injury, then please enter zero.<br>*IN DAYS*<br>How long does it usually take for a patient with a suspected hip fracture to reach you after the injury has occurred? (in HOURS)<br>*In HOURS*	[_][_]days<br><br>[_][_]hours
3.33a<br><br><br><br><br><br><br><br>3.33b	How do you manage a hip fracture? (Several may apply)<br>*If Other, please Other Specify must not be blank*<br>Splinting<br>Manipulation<br>Traction<br>Herbal and/or traditional medicines<br>Hospital referral<br>Other Not at all<br>Other specify	 <br>1<br>2<br>3<br>4<br>5<br>6<br>7
xx_warning3	WARNING: please check responses. If 'none of those available' is ticked alongside any of the other options then please amend.	Show the field ONLY if:<br>[f04_s03_v01_33a_mhf(7)] = '1' AND ([f04_s03_v01_33a_mhf(2)] = '1' OR [f04_s03_v01_33a_mhf(2)] = '1' OR [f04_s03_v01_33a_mhf(3)] = '1' OR [f04_s03_v01_33a_mhf(4)] = '1' OR [f04_s03_v01_33a_mhf(5)] = '1' OR [f04_s03_v01_33a_mhf(6)] = '1')
3.34	What percentage of hip fractures do you refer to a hospital or clinic for an X-ray? *0 - 100%*	[_][_][_]%
3.35	What percentage of hip fractures do you refer to a hospital or clinic for fracture management? *0 - 100%*	[_][_][_]%

Fracture equipment

	Is any of the following equipment available for the treatment of patients with fractures:	Available observed	Available reported only	Not available
3.36	**Splints**	1	2	3
3.37	**Slings**	1	2	3
3.38	**Plaster of Paris**	1	2	3
3.39	**Traction**	1	2	3
3.40	**Walking sticks**	1	2	3
3.41	**Crutches**	1	2	3
3.42	**Wheelchair**	1	2	3

SECTION 4: Infrastructure

Power supply

4.01	Does your workplace have electricity from any source (e.g. electricity grid, generator, solar, or other)?	Yes<br>No	1<br>0

If No skip 3 questions (to Q.4.05)

4.02a<br><br><br><br><br><br>4.02b	What is your workplace's main source of electricity?<br>*If Other, please Other Specify must not be blank*<br>Central supply of electricity (e.g. national or community grid)<br>Generator/charged battery storage system<br>Solar system<br>Other<br>Other (specify)	 <br>1<br>2<br>3<br>4
4.03a<br><br><br><br><br><br><br><br><br>4.03b	Other than the main source, does your workplace have a secondary or backup source of electricity?<br>*If yes please do not skip next question*<br>*If YES:* What is the secondary source of electricity?<br>*If Other, please Other Specify must not be blank*<br>No secondary source<br>Central supply of electricity (e.g. national or community grid)<br>Generator/charged battery storage system<br>Solar system<br>Other<br>Other (*specify)*	1 Yes<br>0 No<br><br><br><br>1<br>2<br>3<br>4<br>5
4.04	During the past 7 days, were there any interruptions to the main electricity supply in your workplace?<br>Always available without interruptions<br>Often available (interruptions of less than two hours per day)<br>Sometimes available (frequent or prolonged interruptions of more than two hours per day)<br>There was no electricity	 <br>1<br>2<br>3<br>4

4.05	Do you have a light source available and functioning **today**? (torch acceptable)	Available and functional<br>Available and non-functional<br>Available but functionality unknown<br>Not available	1 [__]<br>2 [__]<br>3 [__]<br>4 [__]
**Communications**			
4.06	Does your workplace have a functioning landline telephone that is available to make calls outside the facility at all times that patient services are offered?	Yes<br>No	1<br>0
4.07	Do you have a functioning mobile phone which you use for work?	Yes<br>No	1<br>0
4.08	If Yes;<br>Do you have credit on your mobile phone today?	Yes<br>No	1<br>0
4.09	If Yes<br>Do you use WhatsApp on your mobile phone to communicate about work? (indicate all that apply)	Yes with patients<br>Yes with families of patients<br>Yes with my colleagues<br>Yes with other traditional bone setters<br>Yes with hospitals and/or clinics<br>No	1<br>2<br>3<br>4<br>5<br>6
4.10	Do you have a functioning computer (includes electronic tablet) which you use for work?	Yes<br>No	1<br>0
4.11	When was it last possible for you to have access to email or internet from your phone or work computer (or electronic tablet)?	Never<br>Today<br>Less than 1 month ago<br>At least 1 month ago<br>Do not know	1<br>2<br>3<br>4<br>9
**Transport**			
4.12a<br><br>4.12b	What transport do you use for visiting patients? (indicate all that apply)<br>*If Other, please Other Specify must not be blank*	Motorbike<br>Private car<br>Private 4WD<br>Taxi car<br>Public bus<br>Bicycle<br>Ox cart<br>Donkey cart<br>Other<br>I don't visit patients<br>Other (specify)	1<br>2<br>3<br>4<br>5<br>6<br>7<br>8<br>9<br>10
4.13a<br><br>4.13b	What transport do you have access to, in order to help patients reach your workplace? (indicate all that apply)<br>*If Other, please Other Specify must not be blank*	Ambulance<br>Motorbike<br>Private car<br>Private 4WD<br>Taxi car<br>Public bus<br>Bicycle<br>Ox cart<br>Donkey cart<br>Other<br>None<br>Other (specify)	1<br>2<br>3<br>4<br>5<br>6<br>7<br>8<br>9<br>10<br>11
**Water**			
4.14a	What is the most commonly used source of water at your workplace?<br>*If Other, please Other Specify must not be blank*	Piped into/onto facility<br>Public tap/standpipe<br>Tubewell/borehole<br>Dug well<br>Spring<br>Rainwater collection<br>Bottled water<br>Delivered by cart/truck<br>Surface water	1<br>2<br>3<br>4<br>5<br>6<br>7<br>8<br>9

4.14b		River<br>Other<br>Other (*specify*)	10<br>11
**Basic client amenities**			
4.15	How many hours each day is this workplace usually open so that patients can present?	[_][_]	
4.16	Do you have a place that is private where you can see patients, that can't be seen or over-heard?	Can't be heard, but can be seen<br>Can't be seen, but can be heard<br>Can't be seen or heard<br>No privacy	1<br>2<br>3<br>4
4.17a	Is there a functioning toilet on the premises that is accessible for patient use?<br>IF YES: What type of toilet?	Yes<br>No	1<br>0
4.17b	*If multiple toilets are available, consider the most modern type*	Flush toilet<br>Ventilated improved pit latrine (VIP)<br>Pit latrine with slab<br>Pit latrine without slab/ open pit<br>Composting toilet<br>Bucket<br>Hanging toilet/hanging latrine<br>No facilities on premises/bush/field	1<br>2<br>3<br>4<br>5<br>6<br>7<br>8
**Healthcare waste management**			
4.18a	How does your workplace finally dispose of medical waste (e.g. bandages)?	Open burning, Flat ground, no protection<br>Open burning, Pit or protected ground<br>Dump without burning, Flat ground, open pit-no protection<br>Dump without burning, Ground or pit with protection (includes burying)<br>Stored in covered container<br>Stored in other protected environment<br>Stored unprotected<br>Other	1<br>2<br>3<br>4<br>5<br>6<br>7<br>8
4.18b	***Probe to arrive at correct response, several options may apply.***	Other (*specify*)	
**Processing of equipment for reuse**			
	Please tell me if the following items for processing reusable equipment are available and functional TODAY		
4.19	Non-electric pot with cover for boiling/steam	Available<br>Not available	1 [__]<br>2 [__]
4.20	Heat source for cleaning non-electric equipment	Available and functional<br>Available and non-functional<br>Available but functionality unknown<br>Not available	1 [__]<br>2 [__]<br>3 [__]<br>4 [__]

	Please tell me if the following resources/supplies used for infection control are available in this facility TODAY<br>*Ask to see the items*	**Observed**	**Reported only**	**Not available**
4.21	Clean running water (piped, bucket with tap, or pour pitcher)	1	2	3
4.22	Handwashing soap, liquid soap	1	2	3
4.23	Alcohol-based hand rub	1	2	3
4.24	Disposable latex gloves	1	2	3
4.25	Waste receptacle (pedal bin) with lid and plastic bin liners	1	2	3
4.26	Sharps container ("safety box")	1	2	3
4.27	Environmental disinfectant (e.g. chlorine, alcohol)	1	2	3

**SECTION 5: Interviewer's observations**			
5.01	**Interview end time** (use the 24 hour-clock) *Use the 24 hour-clock*	[__][__]:[__][__]	
5.02a	Interview completion codes<br>*(only if 1.03a=1 or 1.03a=2)*<br>*If Other, please Other Specify must not be blank*	Completed<br>Respondent unavailable<br>Refused<br>Partially completed<br>Other	1<br>2<br>3<br>4<br>5
5.02b		Other (*specify*)	
5.03	Comments about respondent:		
5.04	Any respondent's comments		

5.05	Researcher’s observations	
5.06	Date: [_][_]/[_][_]/[_][_] (*day/month/year)*	
5.07a	Name of researcher<br>*Please select from the list above*	Show the field ONLY if:<br>[f04_s01_v03a_methoddc] = '1' or [f04_s01_v03a_methoddc] = '2'

Form Status

Complete?	0 Incomplete<br>1 Unverified<br>2 Complete
